# Formulation of Metal–Organic Framework-Based Drug Carriers by Controlled Coordination of Methoxy PEG Phosphate: Boosting Colloidal Stability and Redispersibility

**DOI:** 10.1021/jacs.1c03943

**Published:** 2021-08-06

**Authors:** Xu Chen, Yunhui Zhuang, Nakul Rampal, Rachel Hewitt, Giorgio Divitini, Christopher A. O’Keefe, Xiewen Liu, Daniel J. Whitaker, John W. Wills, Ravin Jugdaohsingh, Jonathan J. Powell, Han Yu, Clare P. Grey, Oren A. Scherman, David Fairen-Jimenez

**Affiliations:** † The Adsorption & Advanced Materials Laboratory (A^2^ML), Department of Chemical Engineering & Biotechnology, University of Cambridge, Philippa Fawcett Drive, Cambridge CB3 0AS, United Kingdom; ‡ Electron Microscopy Group, Department of Materials Science and Metallurgy, University of Cambridge, 27 Charles Babbage Road, Cambridge CB3 0FS, United Kingdom; § Department of Chemistry, University of Cambridge, Lensfield Road, Cambridge CB2 1EW, United Kingdom; ∥ School of Chemical and Environmental Engineering, Shanghai Institute of Technology, No. 100 Haiquan Road, Shanghai 201418, P. R. China; ⊥ Biominerals Research Laboratory & Cellular Imaging and Analysis Facility, Department of Veterinary Medicine, University of Cambridge, Madingley Road, Cambridge CB3 0ES, United Kingdom

## Abstract

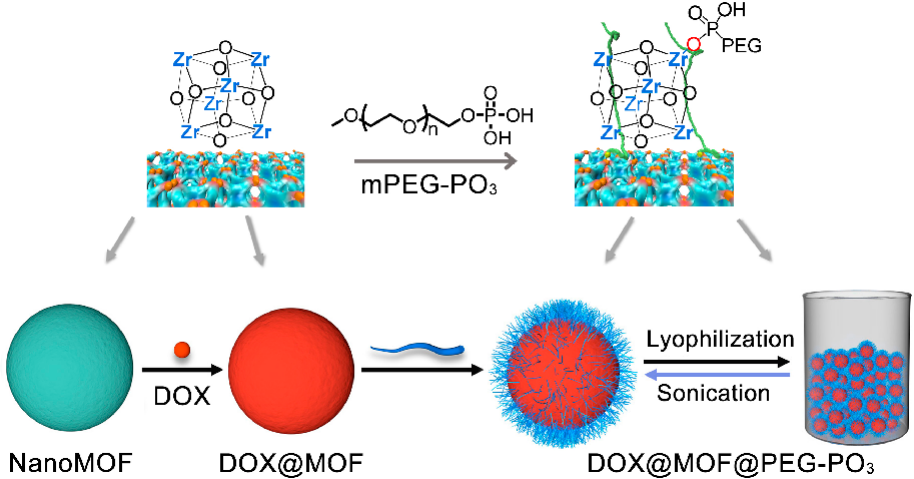

Metal–organic framework nanoparticles (nanoMOFs) have been widely studied in biomedical applications. Although substantial efforts have been devoted to the development of biocompatible approaches, the requirement of tedious synthetic steps, toxic reagents, and limitations on the shelf life of nanoparticles in solution are still significant barriers to their translation to clinical use. In this work, we propose a new postsynthetic modification of nanoMOFs with phosphate-functionalized methoxy polyethylene glycol (mPEG–PO_3_) groups which, when combined with lyophilization, leads to the formation of redispersible solid materials. This approach can serve as a facile and general formulation method for the storage of bare or drug-loaded nanoMOFs. The obtained PEGylated nanoMOFs show stable hydrodynamic diameters, improved colloidal stability, and delayed drug-release kinetics compared to their parent nanoMOFs. Ex situ characterization and computational studies reveal that PEGylation of PCN-222 proceeds in a two-step fashion. Most importantly, the lyophilized, PEGylated nanoMOFs can be completely redispersed in water, avoiding common aggregation issues that have limited the use of MOFs in the biomedical field to the wet form—a critical limitation for their translation to clinical use as these materials can now be stored as dried samples. The in vitro performance of the addition of mPEG–PO_3_ was confirmed by the improved intracellular stability and delayed drug-release capability, including lower cytotoxicity compared with that of the bare nanoMOFs. Furthermore, *z*-stack confocal microscopy images reveal the colocalization of bare and PEGylated nanoMOFs. This research highlights a facile PEGylation method with mPEG–PO_3_, providing new insights into the design of promising nanocarriers for drug delivery.

## Introduction

Metal–organic frameworks (MOFs) have shown great potential in a variety of applications such as gas storage and separation,^1,2^ sensing,^3^ catalysis,^4^ and drug delivery^5−7^ due to their well-defined, tunable structures and permanent porosities. Both the organic linkers and the inorganic nodes could provide attractive platforms to incorporate multiple functionalities onto the MOF’s internal or external surface.^8,9^ Of particular interest is the external surface functionalization of nanoMOFs, which has been extensively explored in biological systems for delivery, imaging, and therapeutic applications. Functionalization typically occurs via postsynthetic modifications, which can improve the colloidal stability and cellular uptake, control drug release, achieve targeted drug delivery, or prolong circulation time.^6^ To date, a number of covalent/coordinative modifications based on either linkers or nodes have been developed.^6,8−10^ For example, Wuttke and Lächelt et al. reported a coordinative binding approach using the high affinity of His-tags toward metal ions.^11^ We previously reported a click modulation strategy to functionalize UiO-66 with PEG moieties using copper(I) iodide as the catalyst.^12^ Horcajada et al. developed a graft-fast functionalization methodology based on aryl radicals to anchor specific molecules onto the external surface of nanoMOFs.^13^ In addition, due to the strong binding affinity between Zr and phosphate, Gu et al. proposed a unique way to protect a porphyrinic nanoMOF from being attacked by phosphate ions using phospholipid bilayers.^14^ Moreover, the groups of Mirkin et al.,^15^ Tan et al.,^16^ and Farha et al.^17^ reported strategies to modify nanoMOFs with phosphate-modified oligonucleotides/DNA. Despite the complexity in preparing modified oligonucleotides/peptides and the introduction of exotic metal species which, in some cases, give rise to long-term cytotoxicity and poor biodegradability, these strategies have proved effective in specific applications. Although great advances have been achieved, very few functionalization strategies have been performed under mild conditions, avoiding complicated synthetic methodologies, costly reagents, or additional toxic catalysts that are incompatible with the use of biological macromolecules (e.g., siRNA, proteins) or with the implementation of good manufacturing practices required for the translation of MOFs to clinical use (Supplementary Table S1).

External surface modification is often combined with biocompatible polymers.^6,8−10^ Among them, polyethylene glycol (PEG) is one of the most common coatings.^18^ Ever since the pioneering work reported by Horcajada and co-workers, which modified Fe(III)-based nanoMOFs through the coordination of monomethoxy-amino-PEG to the Fe ions of the MOF,^5^ modification with PEG bearing diverse terminal functional groups, including monovalent PEG carboxylates,^19^ methoxy PEG–folate,^20^ and PEG-tailed sulfonate,^21^ have been developed. Moreover, among the reported MOFs, Zr(IV)-based MOFs have attracted considerable attention because of their exceptional chemical stability and easy modification with the desired functionality.^22,23^ This has made them suitable candidates for biomedical applications.^24^ However, very little attention has been paid to the process of modification. Remarkably, and although this is generally ignored, in almost all cases, nanoMOFs-based drug carriers cannot be redispersed once dried^6^ and had to be kept in suspension in solution. Again, this limits their translation into the field of drug delivery, as nanoMOFs in solution—and especially those loaded with drugs—will show a limited shelf life arising from potential long-term aggregation and drug release. In view of the existing water-dispersible technologies based on the radical polymerization of vinylic groups,^13,25^ we aimed to develop a formulation strategy for nanoMOF-based carriers using green and mild conditions, which can be easily prepared and redispersed.

**Scheme 1:**
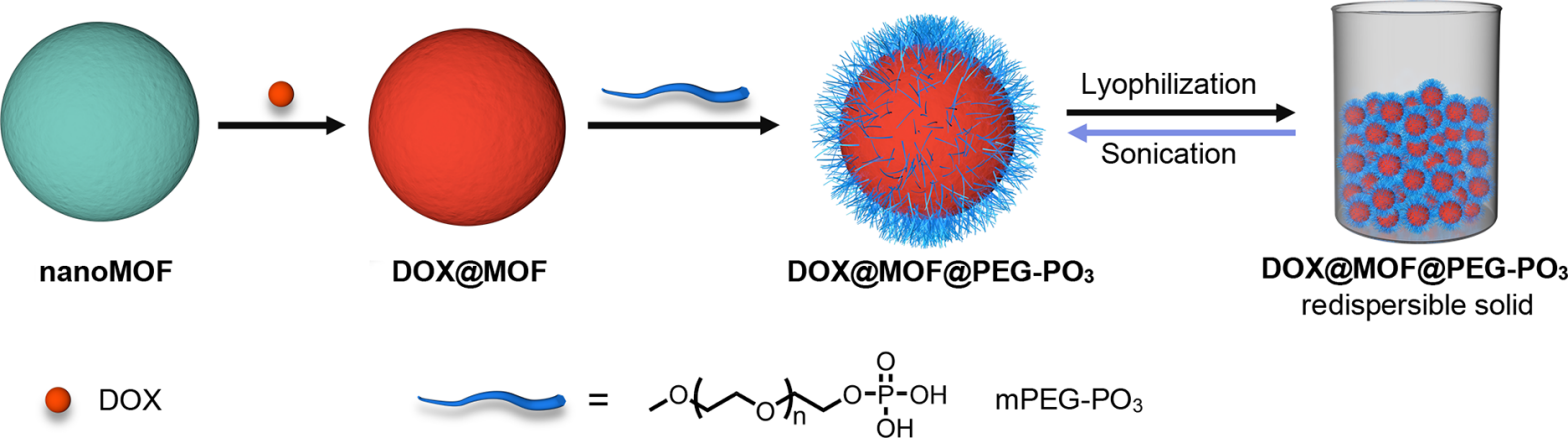
Schematic Illustration of the Synthesis of Redispersible Drug-Loaded NanoMOFs

Here, we report a general formulation strategy for the solidification of bare or drug-loaded Zr–MOFs through a simple PEGylation process using methoxy PEG phosphate (mPEG–PO_3_, *M*_n_ = 5 k)^26^ and lyophilization treatment. This strategy leads to the formation of a low-density solid material, which not only exhibits excellent water redispersibility at room temperature by mild sonication treatment and maintains the hydrodynamic diameter but also shows improved colloidal stability and delayed drug-release kinetics of the encapsulated model drug (Scheme 1). In particular, with nanosized PCN-222 as a model example, we systematically studied the PEGylation process with mPEG–PO_3_ using ex situ time-dependent techniques combined with molecular dynamics (MD) simulations. We also examined the generality and drug storage capabilities of our formulation strategy by successfully extending it to other nanosized Zr–MOFs, including UiO-66, MOF-808, NU-901, and PCN-128 using doxorubicin hydrochloride (DOX) as a model drug. As a proof-of-concept study, we selected HeLa cells and demonstrated that the PEGylated-nanoMOFs exhibited improved intracellular stability and delayed drug-release capability with less cytotoxicity compared with bare nanoMOFs. Furthermore, we also studied the colocalization of bare and PEGylated nanoMOFs through *z*-stack confocal microscopy imaging. Overall, we believe our findings solve a key problem in the evaluation of current MOF-based drug carriers, as most of them are based on freshly prepared materials. Indeed, the proposed materials and methods allow the development of MOF-based drug carriers with improved shelf lives, making them more desirable for pharmaceutical exploitation.

### Synthesis and Characterization of PCN-222 and PCN-222@PEG–PO_3_

**Figure 1:**
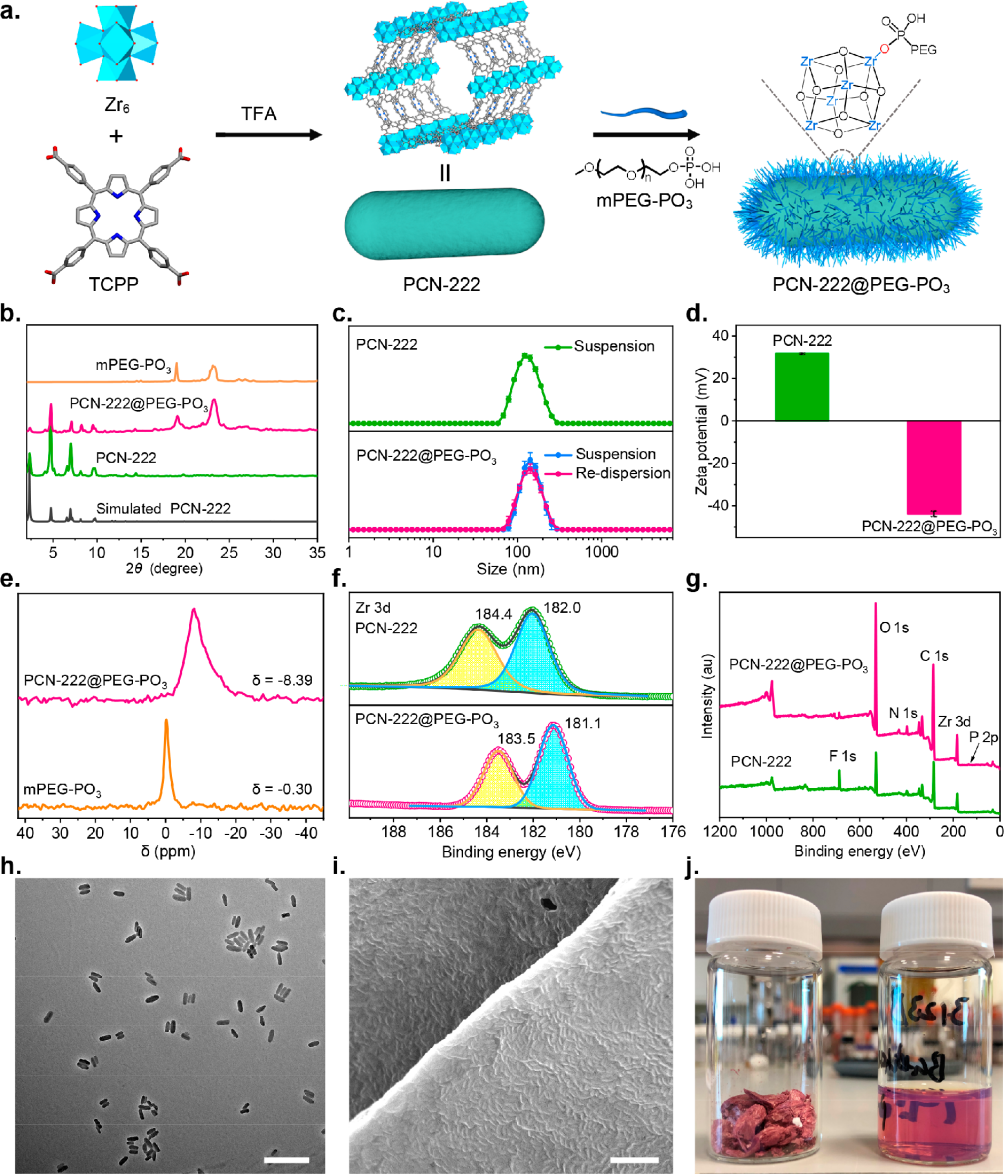
Characterization of PCN-222 and PCN-222@PEG–PO_3_. (a) Representation of the synthesis. (b) Simulated and experimental PXRD patterns. (c) Intensity-average diameter of a water suspension of PCN-222 (green line) and PCN-222@PEG–PO_3_ (blue line) and the redispersed solution of PCN-222@PEG–PO_3_ (pink line) in water (*n* = 3). (d) Zeta potential of water suspensions of PCN-222 and PCN-222@PEG–PO_3_. (e) ^31^P SSNMR spectra. (f)High-resolution Zr 3d spectra. (g) XPS survey spectra. (h) TEM image of drop-cast PCN-222@PEG–PO_3_. (i) SEM image of lyophilized PCN-222@PEG–PO_3_. (j) Photographs of lyophilized PCN-222@PEG–PO_3_ (left) and its redispersed suspension (right). Scale bar: 500 nm.

We first chose PCN-222 as a representative Zr–MOF, a material that has been studied extensively for biological applications due to its large porosity and extraordinary stability under harsh conditions. PCN-222 consists of Zr_6_ clusters with eight edges connected to the tetrakis(4-carboxyphenyl)porphyrin (TCPP) linkers featuring 1D micro- (triangular) and mesoporous (hexagonal) channels with diameters of 1.7 and 3.6 nm, respectively.^27^ Motivated by the success of a previous modulation strategy,^28^ we synthesized the Zr_6_ clusters first and introduced them to promote the synthesis of nanosized PCN-222, as heating the solution of TCPP with ZrCl_4_ tended to result in mixed phases.^29^ Briefly, we prepared nanosized PCN-222 through a solvothermal reaction between TCPP and Zr_6_ clusters using trifluoroacetic acid (TFA) as a modulator (Figure 1a). In this case, the particle size can be readily modulated by tuning the amount of TFA employed, an approach frequently used in the synthesis of other Zr–MOFs.^30^ With higher amounts of TFA, we isolated PCN-222 with an increased particle size (Figure S4, see Supporting Information for full details). For biomedical applications, particle size is crucial;^31^ thus, we chose PCN-222 with an average length of 117.8 ± 12.9 nm. Powder X-ray diffraction (PXRD) shows the presence of broad peaks that match the pattern predicted from the single-crystal structure, confirming the phase purity of PCN-222 (Figure 1b). Transmission electron microscopy (TEM) imaging shows that the PCN-222 nanoparticles have a rod-shaped morphology and good size uniformity (Figure S5a), whereas high-angle annular dark-field scanning transmission electron microscopy (HAADF-STEM) imaging confirms the existence of highly oriented mesopores (Figure S5b). Dynamic light scattering (DLS) of PCN-222 shows an average diameter and polydispersity index of around 118.0 nm and 0.104, respectively (Figure 1c). In turn, the zeta potential of around 31.7 mV (Figure 1d) suggests that the predominant end groups on the external surface are the metal units, which could facilitate the postsynthetic PEGylation with negatively charged mPEG–PO_3_.

We next prepared mPEG–PO_3_ from commercially available poly(ethylene glycol) methyl ether (*M*_n_ = 5 k) (Scheme S1). NMR spectroscopy and size exclusion chromatography with multiangle light scattering (SEC-MALS) confirm the formation and purity of mPEG–PO_3_ (Figures S1–S3). We performed the PEGylation of PCN-222 by mixing an aqueous suspension of the PCN-222 with an aqueous solution of mPEG–PO_3_ at room temperature for 16 h followed by centrifugation and dialysis. The final product was kept in an aqueous suspension. Importantly, we found that the drying method was critical for the morphology and characteristics of the final products. If the resulting suspension was centrifuged and exchanged with ethanol and then dried in air, a dense and dark-colored sample was obtained (Figure S6a). On the other hand, if the suspension was lyophilized, a brown material with low density was isolated (Figures 1j and S6b). We noted that the ambient-dried material was largely aggregated, but the lyophilized sample could be easily redispersed upon sonication, leading to a pink suspension (Figure 1j, Video S2), which shows the characteristic Tyndall effect with a passing red laser beam, revealing the colloidal nature of the redispersed suspension (Figures S22 and S23b). We denote this new material as PCN-222@PEG–PO_3_. Table S2 shows that the amount of incorporated mPEG–PO_3_ in PCN-222@PEG–PO_3_ is 32.9 wt %, estimated using inductively coupled plasma-optical emission spectroscopy (ICP-OES) by measuring the ratio of P to Zr.

Following the PEGylation process, we characterized the material to demonstrate the successful incorporation of mPEG–PO_3_. PXRD confirms the formation of a crystalline phase, where the first five peaks match both the parent and the simulated patterns well, confirming the maintained crystallinity after PEGylation. A broad new peak at around 2θ = 22.0° and two new peaks centered at 2θ = 19.0° and 23.2° in ambient-dried and lyophilized PCN-222@PEG–PO_3_ were found after PEGylation, which is due to the formation of amorphous and semicrystalline PEG in PCN-222@PEG–PO_3_ (Figures 1b and S24).^32,33^ However, the first peak became weaker in terms of intensity after PEGylation. Given that the channels of PCN-222 are large enough to accommodate linear mPEG molecules,^34^ we reason that this feature is probably related to the mPEG that infiltrated and partially occupied the mesoporous cavities. Fourier transform infrared (FT-IR) spectra provide further evidence for the existence of mPEG–PO_3_, where the appearance of two new bands at 2866 and 1088 cm^–1^ are attributed to the stretching vibrations of C–H and P–O, respectively, from the mPEG–PO_3_ molecules (Figure S28a).^35^ In particular, we observed a shift in the FT-IR absorption from 1095 to 1088 cm^–1^ for the P–O bonds in the PCN-222@PEG–PO_3_ compared to that of mPEG–PO_3_ (Figure S28b), indicating the presence of an interaction between the Zr_6_ cluster and the phosphate group, consistent with data in previous reports.^32,36^ TEM imaging of drop-cast PCN-222@PEG–PO_3_ clearly shows the well-preserved morphology and monodispersed particles of the parent MOF (Figure 1h). Moreover, HAADF-STEM imaging demonstrates the preservation of highly ordered mesopores after PEGylation (Figure S7). We also employed DLS to investigate the effect of PEGylation on the dispersity of PCN-222, obtaining a particle diameter of 129.7 ± 0.9 nm, which is slightly larger than that of the parent PCN-222 (Figure 1c). On the other hand, the polydispersity index was reduced to a value of 0.076 ± 0.004, which can be explained by the fact that the PEGylation process disperses the initial, existent small aggregates in the parent PCN-222 sample. Figure 1d shows that the zeta potential becomes negative, from 31.7 ± 0.4 mV for the parent PCN-222 to −43.9 ± 1.2 mV for PCN-222@PEG–PO_3_, attributed to the attachment of terminal phosphate groups on the external surface of the PCN-222 sample.^37^

We next performed ^31^P solid-state nuclear magnetic resonance (SSNMR) and X-ray photoelectron (XPS) spectroscopies to study the interaction between mPEG–PO_3_ and the framework. Figure 1e shows the chemical shift of the phosphorus resonance in solid-state PCN-222 and PCN-222@PEG–PO_3_. Free mPEG–PO_3_ features a sharp peak at around −0.30 ppm; after PEGylation, the peak becomes broadened and upshifts to −8.39 ppm. The broadened peak is due to the accumulation of the slightly different chemical shifts, which originate from the location of mPEG–PO_3_ on different binding sites of the Zr_6_ cluster since both the −OH and the −OH_2_ groups could provide anchoring sites.^8,38^ In addition, the shift in the phosphorus resonance suggests an interaction between the phosphate group of the mPEG–PO_3_ and the framework, similar to a previously reported oligonucleotide-functionalized MOF.^15^ The XPS survey and high-resolution scan of the P 2p spectra confirm the existence of P in PCN-222@PEG–PO_3_ (Figures 1g and S20), which presumably belongs to the P of incorporated mPEG–PO_3_. Furthermore, in the high-resolution Zr 3d spectra, two main peaks at 184.4 and 182.0 eV, ascribed to the 3d_3/2_ and 3d_5/2_ of Zr(IV) in parent PCN-222,^39^ shift to 183.5 and 181.1 eV, respectively (Figure 1f). The shift in binding energy could mainly be due to the change in the chemical environment that Zr atoms experience.^40^ In this case, replacement of the trifluoroacetate group by the relatively weak electronegative phosphate moiety in mPEG–PO_3_ causes a less electron-withdrawing effect on the Zr_6_ clusters, resulting in a negative shift of the XPS peaks for Zr.^41^ Similar results have been reported for another Mn-decorated Zr–MOF.^42^Figure 1g shows that the F 1s peak of parent PCN-222 at 688.2 eV is significantly weakened after PEGylation, suggesting that the amount of bound TFA decreases. Taken together, the shift of the chemical resonances^15,43^ and binding energies^36,44^ after PEGylation suggests the formation of the Zr–O–P coordination bonds.^45,46^

To better visualize the presence of mPEG–PO_3_ in PCN-222@PEG–PO_3_, we performed SEM on the dried samples. We clearly observe that nanoMOFs are tightly embedded within the matrix in the lyophilized PCN-222@PEG–PO_3_; we hypothesize that the matrix is formed by the surface-attached mPEG–PO_3_ molecules (Figures 1i and S8), which can act as spacers, thus physically isolating the individual PCN-222@PEG–PO_3_ particles. In contrast, SEM of ambient-dried PCN-222@PEG–PO_3_ reveals monodispersed particles randomly packed into centimeter-sized three-dimensional superstructures (Figure S9), where the matrix observed in the lyophilized samples vanishes. This suggests that in the ambient-dried samples, the mPEG–PO_3_ molecules closely adhere to the particles; similar isolated particles were found in previously reported strategies.^12,15,47^ In addition, the nanoMOFs’ external surface in the ambient-dried PCN-222@PEG–PO_3_ becomes rough compared to the parent PCN-222, as their external surface features become dominated by the bulk of their capping polymers, which form nanopapillae.^48^

**Figure 2:**
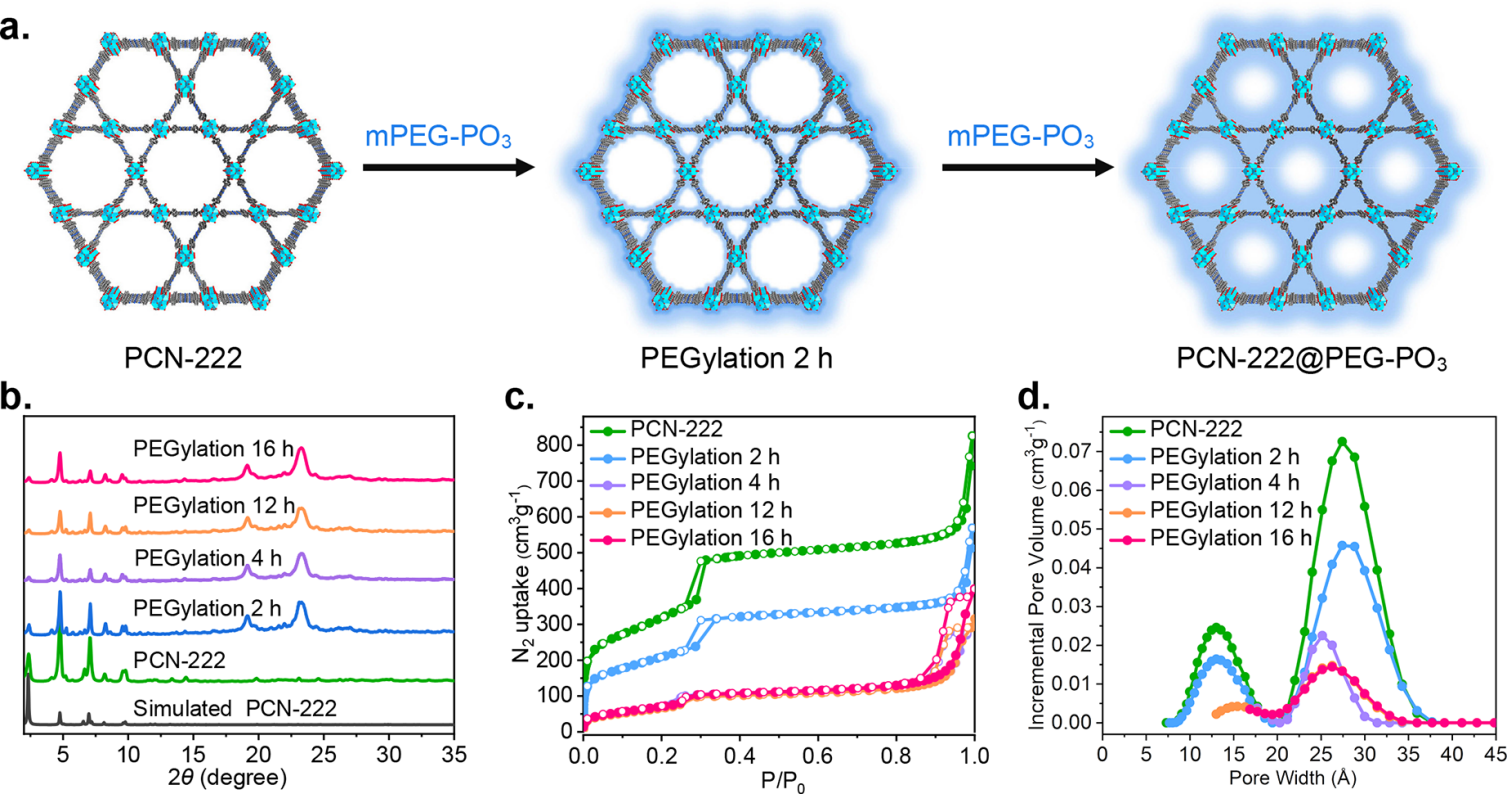
Time-dependent studies of PCN-222@PEG–PO_3_. (a) Proposed schematic of PCN-222@PEG–PO_3_ formation. Characterization recorded at different intervals of time (2, 4, 12, and 16 h). (b) Simulated and experimental PXRD patterns. (c) N_2_ isotherms at 77 K. (d) PSD obtained with the NLDFT method.

We next conducted N_2_ adsorption experiments at 77 K to investigate the potential impact of incorporating mPEG–PO_3_ into the internal porosity of PCN-222. As shown in Figure 2c, PCN-222 and PCN-222@PEG–PO_3_ adsorb 526 and 129 cm^3^ g^–1^ N_2_ at *P*/*P*_0_ = 0.8, respectively, with Brunauer–Emmett–Teller (BET) areas, analyzed using BETSI,^49^ decreasing from 1151 to 265 m^2^ g^–1^ (see Supporting Information, Section S4). However, the pure mPEG–PO_3_ adsorbs a nearly negligible amount of N_2_ (Figure S33). Assuming that all of the included mPEG–PO_3_ molecules are located at the external surface, the ideal N_2_ uptake at *P*/*P*_0_ = 0.8 would be 353 cm^3^ g^–1^ after subtracting the amount of mPEG–PO_3_ included (Table S3). The actual value obtained here, however, is only 36.5% of this ideal N_2_ uptake where the PEG chains do not affect the internal porosity of PCN-222. The discrepancy between the ideal value and the experimental one is most likely due to the infiltration of mPEG–PO_3_ molecules into the internal porosity of PCN-222 after performing PEGylation for 16 h. This is quantified further by the pore size distribution (PSD) obtained using the non-local density functional theory (NLDFT) method.^50^ We note that the calculated average pore size of the parent PCN-222 features two types of pores at 13 and 27 Å, consistent with reported results.^51^ The incremental pore volume of PCN-222 greatly reduces after performing PEGylation for 16 h (Figure 2d), which accounts for 33.5% of that of the parent PCN-222 after correction by subtracting the amount of mPEG–PO_3_ included. We also note that the calculated average pore size slightly decreased by 1.2 Å (Figure S36). Overall, this reveals a partial infiltration of the mPEG–PO_3_ chains, blocking the accessibility of the nitrogen toward the internal porosity. The results reveal that our method modifies the external surface with a partial sacrifice of its porosity.

### Time-Dependent PEGylation

Despite the fact that the use of phosphate-containing PEG for modification of nanoMOFs has been reported, examples are rare, and the process of PEGylation is still unclear.^26^ To better understand the PEGylation process, we next performed time-dependent studies on PCN-222 by quenching the PEGylation with centrifugation and dialysis at different reaction times and analyzing the intermediates with a number of ex situ spectroscopic and microscopic methods. We named these obtained PEGylated PCN-222 as PEGylation *x* h, where *x* is the reaction time (*x* = 2, 4, 12, and 16). Figures 2b and S28 show no significant change in their PXRD patterns and FT-IR spectra after 2 h. ICP-OES quantifies the amount of mPEG–PO_3_ at different PEGylation times by measuring the ratio of Zr to P (Table S2). The results show rapid incorporation of mPEG–PO_3_; the loading of mPEG–PO_3_ reaches 27.8 wt % after 2 h. With increased reaction time, the values reach a plateau at around ∼33 wt %. In addition, TGA profiles show a similar trend (Figure S26); we also note that PEGylated PCN-222 is less thermally stable than the parent PCN-222, which is probably due to the movement and decomposition of the attached PEG chains.^52^ We next examined their N_2_ adsorption isotherms to investigate the location of mPEG–PO_3_ at different reaction times. After 2 h, the N_2_ uptake at *P*/*P*_0_ = 0.8 decreases from 526 cm^3^ g^–1^ for the parent PCN-222 to 348 cm^3^ g^–1^ (Figure 2c), accounting for 91.6% of the ideal N_2_ uptake (Table S3) with a BET area of 756 m^2^ g^–1^. The effect of mPEG–PO_3_ over porosity after 2 h can be more easily observed after normalizing the N_2_ isotherms using the MOF mass only. As shown in Figures 2d and S34, N_2_ adsorption shows only a small decrease in uptake, while the shape of its PSD and the calculated average pore size match with that of the parent PCN-222 very well, with the incremental pore volume accounting for roughly 90% of that of the parent PCN-222. We attribute this to the fact that mPEG–PO_3_ completely blocks the porosity of few MOF particles and leaves the rest of the particles unaffected. Altogether, these results suggest that PEGylation mainly occurs on the surface at the early stage while not significantly affecting its internal porosity. Afterwards, with continuous PEGylation for 4 h, the amount of mPEG–PO_3_ increases up to 33.3 wt % (Table S2), the corresponding N_2_ uptakes at *P*/*P*_0_ = 0.8 and BET area decrease to 124 cm^3^ g^–1^ and 252 m^2^ g^–1^, respectively. The N_2_ uptakes at *P*/*P*_0_ = 0.8 obtained accounts for 35.3% of the ideal N_2_ uptake, and these values become constant even after prolonging the reaction times to 12 and 16 h (Table S3), indicating that PEGylation has reached an equilibrium with a large number of mPEG–PO_3_ molecules entering the porosity. We assume that the excess of mPEG–PO_3_ molecules starts to infiltrate into the channel after fully occupying the available binding sites at the external surface. In addition, the incremental pore volume decreases greatly, whereas the calculated average pore size reduces slightly after performing PEGylation for 4 h and more (Figures 2d and S35a). Again, after normalizing the N_2_ isotherms by subtracting the amount of mPEG–PO_3_ included (Figure S35b), we evaluated the loss of porosity as a function of reaction time on the basis of the roughly same amount of mPEG–PO_3_ included (Figure S38). From this plot we can clearly see a 53.9% loss of porosity after performing PEGylation for 4 h and then a gradual increase to around 60% in the following 8 h before reaching a plateau, suggesting the infiltration of mPEG–PO_3_ into the internal porosity, thus partially blocking the pore. Combined with the results that the loading of mPEG–PO_3_ is consistent after 4 h (Table S2), here we attribute the loss of porosity after 4 h to the dangling PEG chains of surface-bonded mPEG–PO_3_ infiltrating into the porosity. Taken together and based on the above results, we propose a two-step PEGylation process for PCN-222 with mPEG–PO_3_, this is, the PEGylation takes place first at the external surface, probably due to the electrostatic interaction between positively charged PCN-222 and negatively charged mPEG–PO_3_.^53^ After occupying the available binding sites at the external surface, the unreacted mPEG–PO_3_ molecules start to enter the internal channel of PCN-222, thus partially blocking the porosity (Figure 2a).

**Figure 3:**
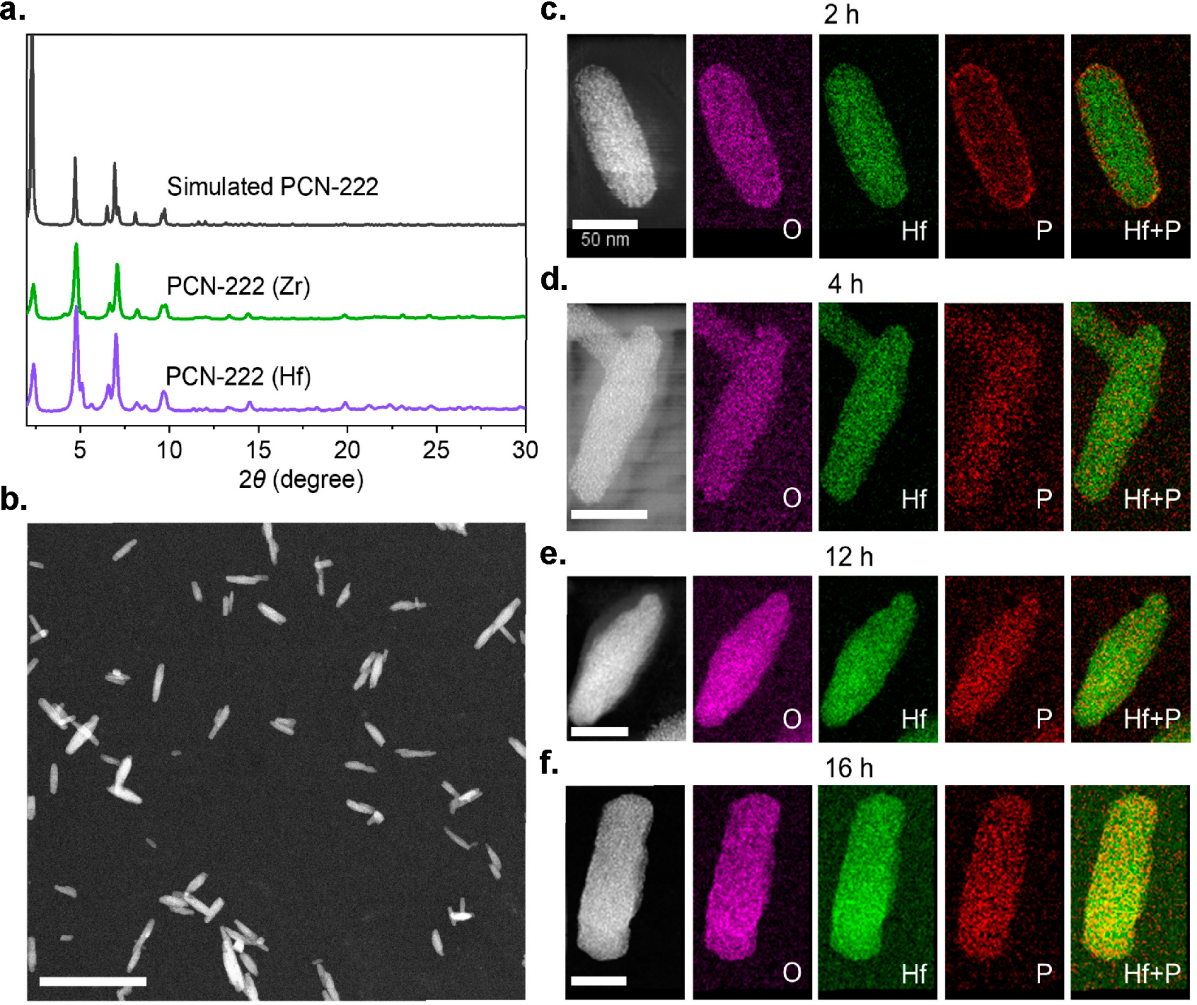
(a) Simulated and experimental PXRD patterns of PCN-222 (Hf). (b) HAADF-STEM image of PCN-222 (Hf). Scale bar: 500 nm. (c–f) HAADF-STEM images and EDX elemental maps of O, Hf, P, and overlapped Hf and P recorded at different reaction times: (c) 2, (d) 4, (e) 12, and (f) 16 h. Scale bar: 50 nm.

Considering the presence of P in mPEG–PO_3_, we next conducted the elemental mapping of the samples employing energy-dispersive X-ray (EDX) spectroscopy with STEM. In our case, since the Lα line for Zr is 2.042 keV and the Kα line for P is 2.012 keV,^54^ the energy window created for P would overlap with that of the Zr signal, thus leading to misidentification of the elements.^55^ Although correction can be performed by subtracting the contribution of the overlapping peak, it should be avoided in view of the uncertainties in the correction.^56^ We addressed this issue by simply replacing Zr-based PCN-222 with Hf-based PCN-222, denoted as PCN-222(Hf), whose lines are separate from those of P and Zr.^54^ PXRD and N_2_ adsorption confirm the crystallinity and porosity of PCN-222 (Hf) (Figures 3a and S37), whereas STEM imaging and the XPS spectrum clearly show the rod morphology of PCN-222 (Hf) and the presence of Hf (Figures 3b and S21). Under identical PEGylation conditions as those of Zr-based PCN-222, we obtained the ex situ STEM-EDX of PCN-222 (Hf) after 2, 4, 12, and 16 h, which can map out the distribution of P and Hf. After 2 h, Figure 3c shows that the signal of Hf is evenly distributed throughout the whole particle, whereas the P signal mainly originates from the edges; this provides further evidence to demonstrate that the mPEG–PO_3_ molecules mainly graft the external surface at the early stage. With the increment of reaction time from 4 to 16 h, Figure 3d–f shows that P signals are uniformly distributed throughout the whole particle. In addition, we also performed EDX line scans for PCN-222(Hf) after PEGylation of 16 h, which demonstrates the homogeneous distribution of Hf and P along the single particle (Figure S19). The results observed here are consistent with TGA, N_2_ adsorption, and ICP-OES as mentioned above, which firmly confirms our hypothesis that the PEGylation of PCN-222 with mPEG–PO_3_ proceeds in a stepwise fashion (Figure 2a).

### Molecular Dynamics (MD) Simulations

**Figure 4:**
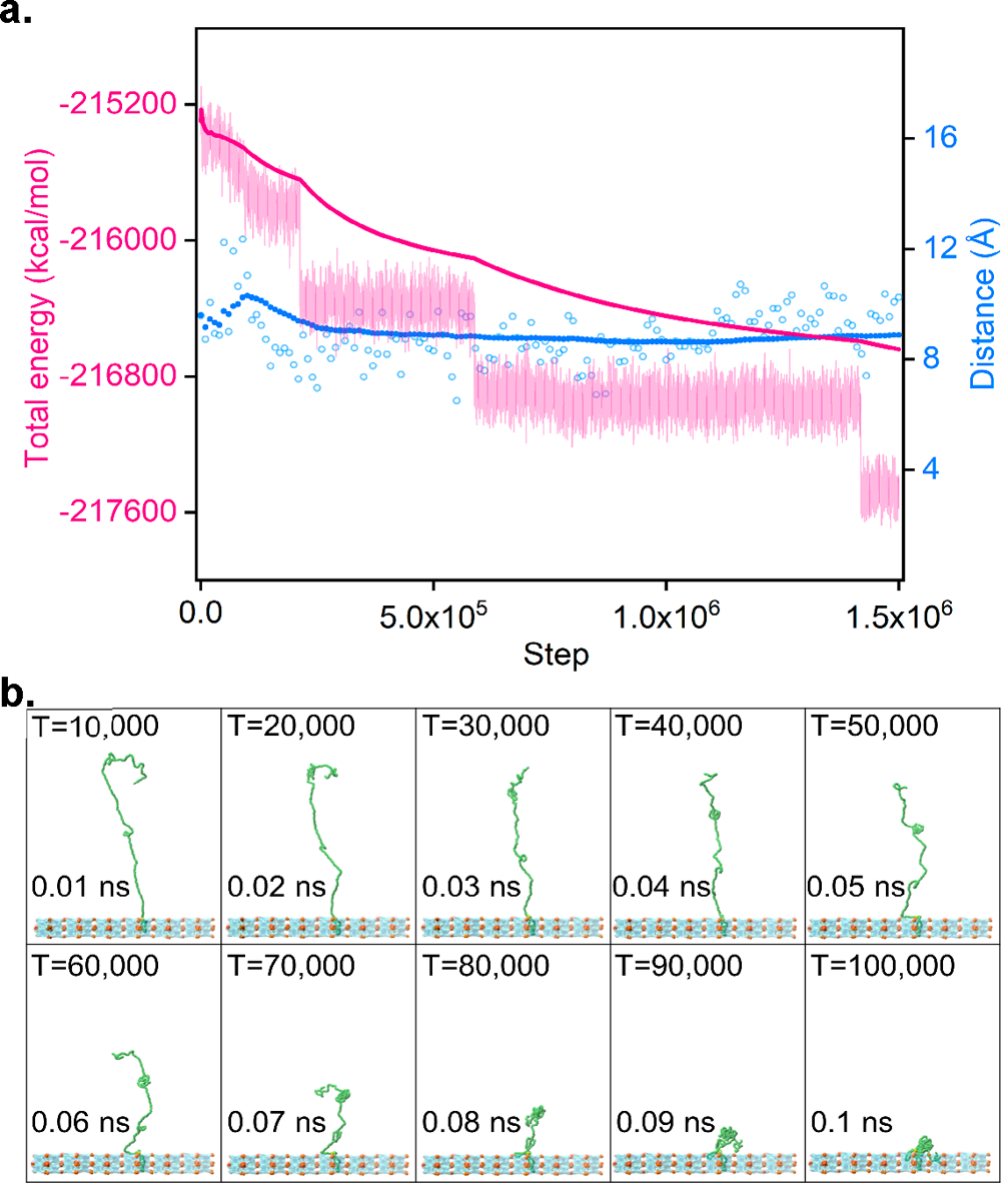
Molecular dynamics simulations. (a) Calculated energy and distance relative to the framework as a function of time steps. (Left *y* axis) Total energy in kcal/mol. (Right *y* axis) Distance between the phosphate group and the Zr atom (Angstroms). (*x* axis) Time step is plotted in femtoseconds. Light pink points represent the instantaneous total energy, and pink points represent the cumulative average total energy computed at every time step. Blue hollow points represent the instantaneous distance, and blue points represent the cumulative average distance computed every 10 000 time steps. (b) Position of the mPEG–PO_3_ relative to the framework as a function of time. PEG atoms are in green, phosphate group is in yellow, framework atoms are in turquoise (translucent), and Zr atoms of the framework are in brown.

We next performed MD simulations to investigate the PEGylation process; this allows us to gain insights into how the presence of mPEG–PO_3_ affects the energetics of the whole system (see Supporting Information, Video S1). Figure 4a shows the cumulative average total energy of the system decreasing continuously as a mPEG–PO_3_ molecule approaches the Zr atom on the external surface of the framework, suggesting the PEGylation process is energetically favorable. We note that the total instantaneous energy of the system stabilizes after mPEG–PO_3_ reaches the surface of the framework, indicating that this is an energetic minimum, equilibrium position of mPEG–PO_3_. We also note some metastable equilibrium states, but the average distance between the phosphate group of mPEG–PO_3_ and Zr (on the surface of the framework) remains constant. This implies that the change in the equilibrium state is due to the conformational rearrangements of the PEG chain on the surface of the framework.^57^Figure 4b shows the position of mPEG–PO_3_ relative to the framework as the simulation proceeds. At 0.01 ns, one can clearly see mPEG–PO_3_ reaching the surface of the framework and the phosphate group of the mPEG–PO_3_ anchoring itself to the Zr on the surface of the framework. Further analysis of the trajectories (Figure S46) tells us that only parts of the PEG chain dangle into the pores of the framework, as can be seen from Figure 4b at 0.1 ns, while the remaining large part of the PEG chain exists as a coil at the external surface. This behavior is in line with our hypothesis that the majority of mPEG–PO_3_ does not enter the pore but anchors itself at the Zr site on the surface of the framework at an early stage.

### Generality of the PEGylation Method and DOX Encapsulation

**Figure 5:**
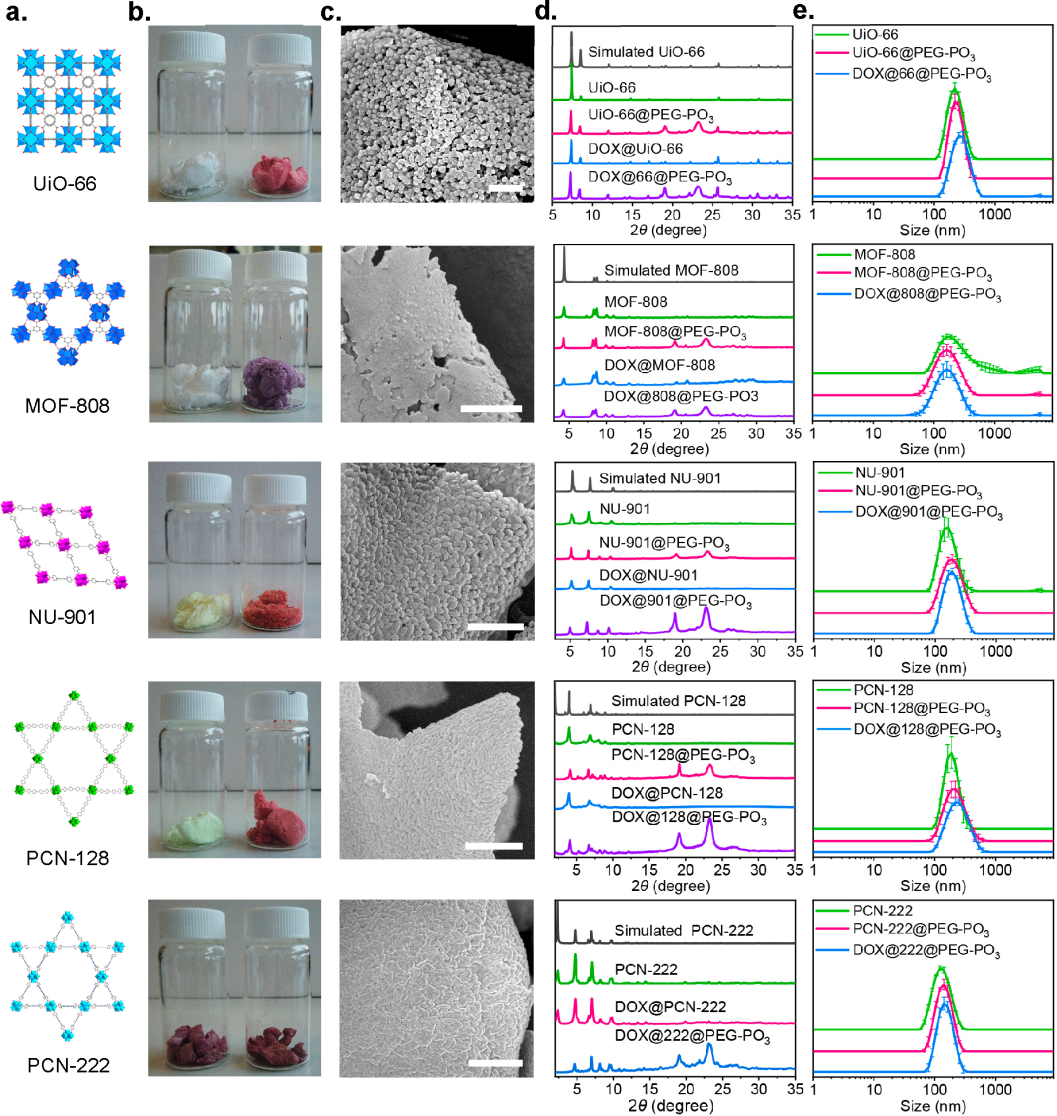
Characterization of MOF@PEG–PO_3_ and DOX@MOF@PEG–PO_3_. (a) Chemical structures of nanoMOFs. (b) Photographs of lyophilized MOF@PEG–PO_3_ (left) and DOX@MOF@PEG–PO_3_ (right). (c) SEM images of lyophilized DOX@MOF@PEG–PO_3_. (d) Simulated and experimental PXRD patterns. (e) Intensity-average diameter of the aqueous suspension of parent nanoMOFs (green line), lyophilized MOF@PEG–PO_3_ (pink line), and DOX@MOF@PEG–PO_3_ (blue line) (*n* = 3). Scale bar: 1 μm.

We next explored the generality of these findings to a broader scope of Zr(IV)-based MOFs, including UiO-66, MOF-808, NU-901, and PCN-128 (Figures 5a and S10). Remarkably, MOFs with distinct pore sizes and morphologies were all tolerated, and we successfully obtained the related lyophilized PEGylated MOFs, denoted as MOF@PEG–PO_3_, by following the same procedure as that of PCN-222. Figure S23a and S23b shows pronounced Tyndall phenomena for bare nanoMOFs and their PEGylated analogues. Figure 5d shows the retention of crystallinities and the presence of semicrystalline PEG after PEGylation, as proven by the PXRD tests. N_2_ adsorption isotherms and PSD show that the porosities are all partially blocked by the infiltrated mPEG–PO_3_, similar to what was observed for PCN-222@PEG–PO_3_ (Figure S39). Attempts to recover the blocked porosities by thermal decomposition of the incorporated mPEG–PO_3_ molecules were unsuccessful, as MOF@PEG–PO_3_ lost their crystallinities after treatment at 300 °C (Figure S24). The loadings of incorporated mPEG–PO_3_ molecules evaluated by ICP-OES are 37.7, 38.5, 30.6, and 34.1 wt % for UiO-66@PEG–PO_3_, MOF-808@PEG–PO_3_, NU-901@PEG–PO_3_, and PCN-128@PEG–PO_3_, respectively (Table S2). All of the lyophilized MOF@PEG–PO_3_ have low densities (Figure 5b). The SEM images of the lyophilized MOF@PEG–PO_3_ exhibit a uniform distribution of particles within the matrix formed by incorporated mPEG–PO_3_ molecules (Figures S11–S14). Most importantly, all of them can be redispersed easily after mild sonication treatment, and we cannot observe any significant difference in their hydrodynamic diameters compared with the suspensions before lyophilization (Figures 5e, S23b, and S41 and Videos S2 and S3). We also evaluated the colloidal stability of these MOF@PEG–PO_3_ in water and PBS (pH = 7.4). Figure S43 shows that the suspensions of PEGylated MOFs in water can be stable up to 7 days without significant changes in their hydrodynamic sizes. Although the sizes started to increase after 7 days, it still outperforms the corresponding bare MOFs, which aggregate in 1 day. In the case of PBS, we note that the pH has a dramatic impact on the colloidal stabilities of PEGylated MOFs. As shown in Figure S44, MOF@PEG–PO_3_ can maintain their hydrodynamic sizes at most for 36 h at pH 7.4. However, the aggregation rate increased rapidly in pH 6.4 and 8.4 PBS. For comparison, the bare MOFs aggregate dramatically in a very short time in pH 7.4 PBS (Figure S43), which limit their further in vitro use. Altogether, these results suggest that our PEGylation method can act as a versatile strategy to slow down the aggregation in water and PBS buffer.

Previously, we developed partial or complete amorphization strategies to prevent the burst release.^58,59^ In these approaches, the collapsed porosities could trap the drug molecules inside the nanoMOFs, prolonging their diffusion times through the collapsed pore. Similarly, we hypothesize herein that the infiltrated mPEG–PO_3_ molecules could block the drug molecules within the pore, thus providing the possibility of prolonging the diffusion time and slowing down the release of the encapsulated drugs. With these considerations in mind, we next investigated the effect of the infiltrated mPEG–PO_3_ molecules upon drug loading and release.

Using the above nanoMOFs as the model platforms, we chose DOX, an anticancer drug, as a model to examine the loading capacity and release profile. One critical question here is the partially blocked porosities by the infiltrated mPEG–PO_3_ molecules, which may further limit its application in loading any drug molecules. First, when performing the drug encapsulation on the lyophilized MOF@PEG–PO_3_, we found negligible amounts (less than 1 wt %) of DOX loaded. This suggests that the infiltrated mPEG–PO_3_ molecules block access to the internal porosities. Then, we carried out the drug loading before performing PEGylation. Briefly, mixing the suspension of nanoMOFs with an aqueous solution of DOX afforded the drug-loaded nanoMOFs, followed by the addition of an aqueous solution of mPEG–PO_3_, dialysis, and lyophilization. We obtained the DOX-loaded nanoMOFs and denote them and the PEGylated nanoMOFs as DOX@MOF and DOX@MOF@PEG–PO_3_. Successful DOX encapsulations were indicated by changes in the nanoMOFs color (Figures 5b and S11–S15). As verified by UV–vis spectroscopy, 17.6, 14.4, 22.2, 18.1, and 23.2 wt % loadings of encapsulated DOX were obtained for the bare UiO-66, MOF-808, NU-901, PCN-128, and PCN-222, respectively. We also observed a slight decrease to 15.1, 13.4, 20.2, 13.6, and 15.5 wt %, respectively, after PEGylation (Figure S29 and Table S4), likely due to partial infiltration of mPEG–PO_3_ in the porosity. For simplification, we denote these materials as DOX@66@PEG–PO_3_, DOX@808@PEG–PO_3_, DOX@901@PEG–PO_3_, DOX@128@PEG–PO, and DOX@222@PEG–PO_3_. The PXRD patterns confirm that the crystallinities remain intact after the DOX loading, and two new peaks at 2θ = 19.0° and 23.2° appear after the PEGylation, similar to that of MOF@PEG–PO_3_ (Figure 5d). The presence of the characteristic absorption peaks in the UV–vis spectra near 486 nm of DOX in DOX@MOF@PEG–PO_3_ and the FT-IR peaks at 1280 cm^–1^ (C–O–C, stretching vibration) in DOX@MOF and DOX@MOF@PEG–PO_3_ indicate the successful incorporation of DOX molecules (Figures S28 and S30).^60^ The N_2_ isotherms show that all of the DOX@MOFs exhibit a decrease in the BET areas and pore volumes after the introduction of DOX molecules (Figure S40 and Table S4). These results are consistent with the idea that a considerable portion of the DOX molecules occupy the internal porosity. We also note that the DOX molecule with a size of 10.3 Å × 15.8 Å is difficult to pass through the opening window of UiO-66 (Figure S27).^61^ However, we still achieved 17.6 and 15.1 wt % DOX loading in the case of DOX@UiO-66 and DOX@66@PEG–PO_3_ together with the mostly maintained PSD; this is likely due to the DOX molecules being mainly located at the defect sites and external surfaces. The following PEGylation leads to further loss of porosities (Figure S40), consistent with the above result of MOF@PEG–PO_3_ in which the drug molecules were not incorporated (Figure 2c and 2d). Expectedly, all of the lyophilized DOX@MOF@PEG–PO_3_ exhibit strong Tyndall effects and excellent redispersity, comparable to that of MOF@PEG–PO_3_ (Figures 5e, S23c, and S41 and Videos S2 and S3). It should be noted that nanoMOFs have been extensively studied as drug carriers, but none of them focused on the final formulation of the MOF–drug composites, which have the capability of long-term storage while maintaining the original hydrodynamic sizes.

We also performed grand canonical Monte Carlo (GCMC) simulations to investigate the adsorption of DOX in these five bare nanoMOFs (see Supporting Information, Section S5). The maximum DOX loadings were calculated to be 48.7, 34.6, 67.0, and 56.5 wt % for the bare MOF-808, NU-901, PCN-128, and PCN-222, respectively. In the case of DOX encapsulation in the UiO-66 model, we obtained zero uptake due to the mismatched sizes (Table S4), which further validates our hypothesis that the adsorption of DOX molecules occurs at the defect sites or external surfaces. Figures S47–S50 show the snapshots of the adsorption process of DOX at low, medium, and saturated loadings, which clearly exhibit the adsorption behavior of DOX. Briefly, DOX molecules are first adsorbed on the walls of the frameworks at low loadings before filling up the whole cavity at higher loadings. As for PCN-128 and PCN-222, which contain both micro- and mesoporous channels, DOX molecules tend to fill the bigger pore first at low loadings followed by the smaller pore (Figures S49 and S50).

We next investigated the effect of the incorporated mPEG–PO_3_ on DOX release behavior in PBS (pH = 7.4). We chose drug-containing UiO-66, NU-901, PCN-128, and PCN-222 and the related PEGylated counterparts to compare their cumulative release. Figure S45 shows the release kinetics of DOX@MOF and DOX@MOF@PEG–PO_3_. In the case of UiO-66, we observed a rapid release of DOX in both PEGylated and bare UiO-66, and no significant differences were found in their release kinetics in 200 h (Figure S45a). This is most likely owing to the fact that DOX is attached to the external surface, consistent with their mismatched sizes (Figure S27). As for the other three MOFs (NU-901, PCN-128, and PCN-222), the release profiles of the PEGylated nanoMOFs show a delay compared to that of the bare ones. In particular, during the first 6 h, less than ∼20% of DOX was released whereas the bare nanoMOFs released 38.4%, 52.5%, and 40.1%. Having demonstrated the delayed release with the presence of mPEG–PO_3_, we also tested the time-dependent stabilities of DOX-loaded bare and PEGylated MOFs in water and PBS (pH = 7.4). As shown in Figures S16 and S25, both bare and PEGylated MOFs remained stable in water after 14 days. However, in the case of PBS (pH = 7.4), bare MOFs decomposed rapidly after 2 h and degraded mostly at 12 h while the morphologies of PEGylated MOFs can be maintained up to 36 h. Afterwards, the particles began to degrade or aggregate (Figure S17), as confirmed by their PXRD patterns (Figure S25). We attribute the enhanced stabilities of PEGylated MOFs to the protection of mPEG–PO_3_, which serves as a shield around the MOF particles. In addition, to demonstrate the retention of framework integrity and porosity of PEGylated MOFs after treatment with PBS (pH = 7.4), we collected EM images using cryogenic electron microscopy (cryo-EM), where the cryogenic temperature can reduce radiation damage induced by the electron beam. Figure S18 shows the cryo-EM images of PBS-treated (pH = 7.4, 24 h) UiO-66@PEG–PO_3_ and PCN-222@PEG–PO_3_, which clearly indicates the preserved ordered channels and also suggests that PEGylation with mPEG–PO_3_ does not even partially decompose the internal porosities. Altogether, these results demonstrate that our PEGylation strategy can provide a protective shield around nanoMOFs, which simultaneously prevents the frameworks from rapid degradation and achieves controllable drug release. Most importantly, this can be a feasible way for the long-term storage of MOF-based drug carriers.

### Cytotoxicity and Cellular Uptake

**Figure 6:**
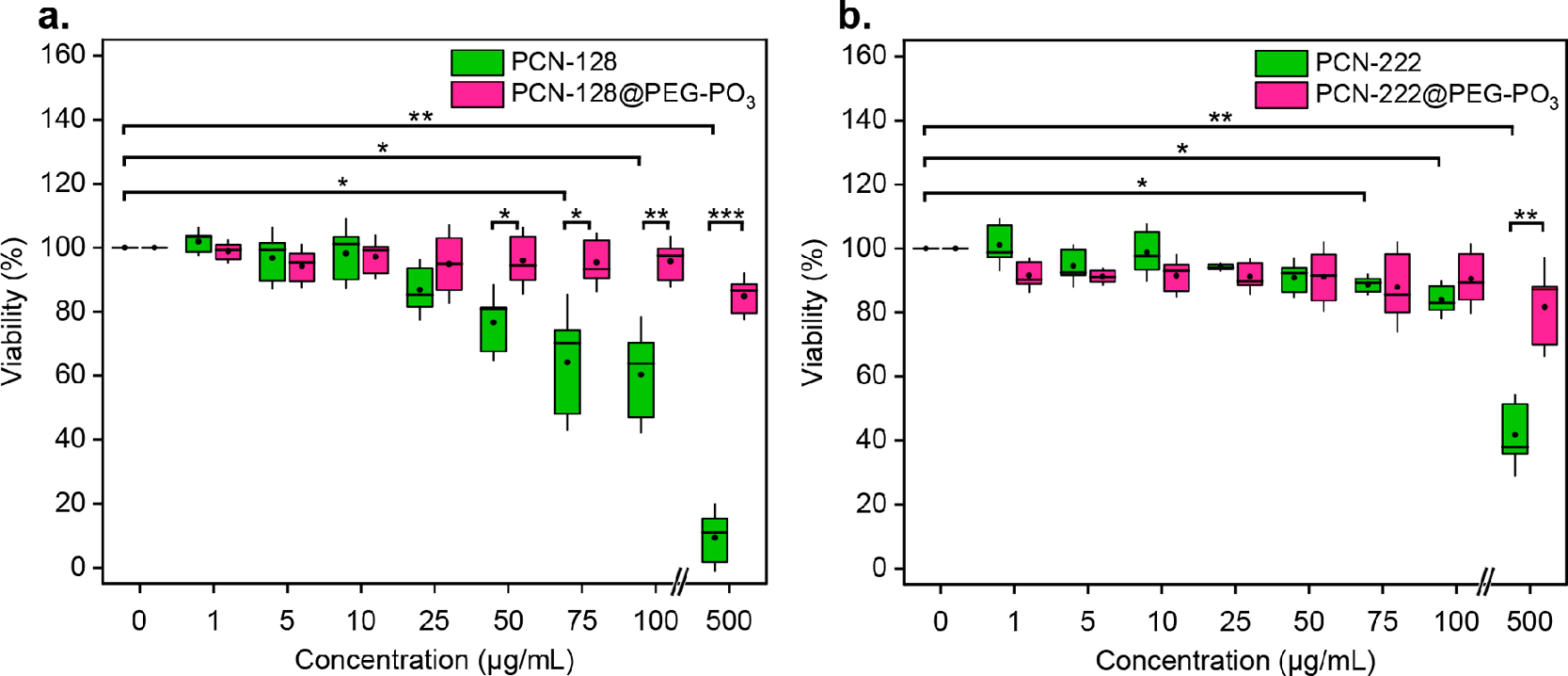
Cytotoxicity of bare nanoMOFs and MOF@PEG–PO_3_. HeLa cells’ viability was measured by MTS assay after a 72 h incubation of (a) PCN-128 vs PCN-128@PEG–PO_3_ and (b) PCN-222 vs PCN-222@PEG–PO_3_ at the same concentration based on nanoMOFs. Statistical analyses for bare PCN-128 and PCN-222 between each concentration and control are annotated with bars. Separate statistical analysis using a paired *t* test was performed between bare PCN-128/PCN-222 and their PEGylated counterpart at a given concentration, as shown directly above the grouped boxplot accordingly (*n* = 3; *** *p* ≤ 0.001, ** *p* ≤ 0.01, * *p* ≤ 0.05).

Following the release analysis, we next chose PCN-222 vs PCN-222@PEG–PO_3_ and PCN-128 vs PCN-128@PEG–PO_3_ as models for in vitro studies due to their fluorescent nature and large accessible porosity. We first evaluated the cytotoxicity of bare and PEGylated nanoMOFs by 3-(4,5-dimethylthiazol-2-yl)-5-(3-carboxymethoxyphenyl)-2-(4-sulfophenyl)-2*H*-tetrazolium (MTS) assay. As shown in Figure 6a, bare PCN-128 shows significant cytotoxicity at concentrations of 50, 75, and 100 μg/mL and 72 h, followed by a sharp fall in viability at an extremely high concentration of 500 μg/mL. As for the bare PCN-222, we observed a similar trend, where it seemed to be biocompatible at concentrations below 100 μg/mL (Figure 6b), but the cytotoxic effect was also observed at 500 μg/mL for 72 h. Dose-dependent toxicity has recently been reported for slightly smaller (15–20 nm diameter) zirconia oxide nanoparticles (ZrO_2_NPs) with toxicity evident at much lower concentrations than the Zr–MOFs we examined using a cell line.^62^ Similar findings to our own were reported by Wang et al. also using a cancer-derived cell line. This group reported 80% viability up to a concentration of 100 μg/mL for a similar porphyrinic Zr–MOF.^63^ In contrast with bare MOFs, we did not observe any significant differences in viability for HeLa cells treated with PCN-128@PEG–PO_3_ and PCN-222@PEG–PO_3_, with viability remaining above 80% at any tested concentration of PEGylated nanoMOFs for 72 h exposure (Figure 6). At high concentrations, 50 μg/mL and above, we observed a significant increase in viability for PCN-128@PEG–PO_3_ compared to bare PCN-128. Importantly, PEGylation also solved PCN-222’s toxicity compared to its bare PCN-222 counterpart at 500 μg/mL. The cytotoxicity of bare nanoMOFs could be due to the poor colloidal stability and cationic surface charge, which promote aggregation and disrupt cellular integrity.^64^ We hypothesize that the shielding of nanoMOFs’ external surface charge by PEGylation improves their biocompatibility and colloidal stability in solution, as described by others.^65,66^

**Figure 7:**
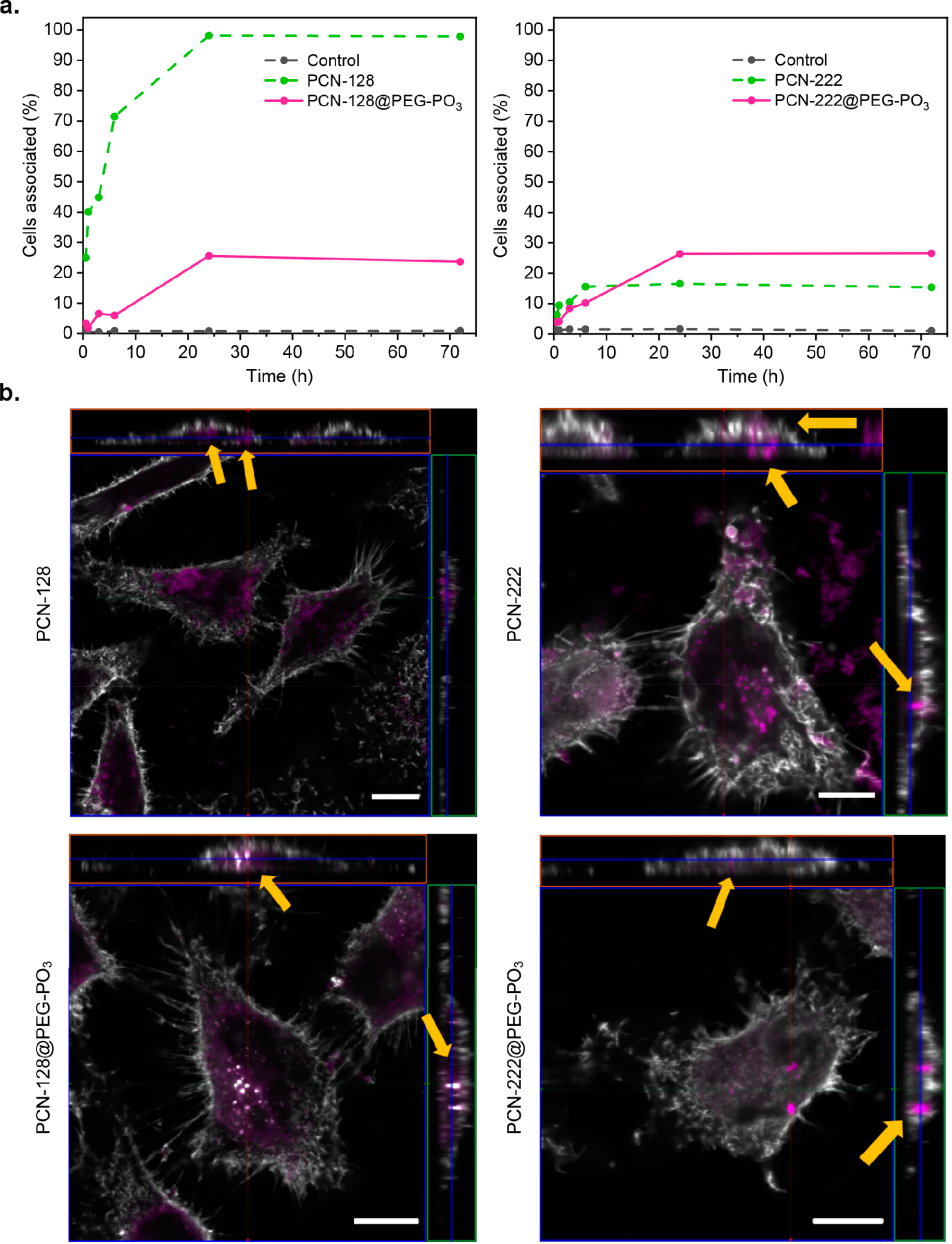
Cellular uptake of PCN-128, PCN-128@PEG–PO_3_, PCN-222, and PCN-222@PEG–PO_3_. (a) Flow cytometry analysis of HeLa cells treated with bare and PEGylated nanoMOFs at equivalent nanoMOF concentrations of 10 μg/mL. (b) Orthogonal projections of *z*-stack confocal slices of single HeLa cells counterstained for the cell surface membrane (in white) with nanoMOFs colored in violet. Orthogonal images are shown in transverse (*x*/*y*), sagittal (*x*/*z*), and frontal (*y*/*z*) views with yellow arrows indicating nanoMOFs that reside inside the cell membrane boundary (see Supporting Information for full *z* stacks and Videos S4, S5, S6, and S7). Scale bar: 10 μm.

We then compared the cellular uptake of PCN-128 and PCN-222 and their PEGylated counterparts by flow cytometry and confocal laser scanning microscopy. HeLa cells were treated with either bare nanoMOFs or PEGylated MOFs for 0.5, 1, 3, 6, 24, and 72 h before being detached from the plate for flow cytometry analysis (Figure S51). As shown in Figure 7a, we observed an immediate uptake for the bare PCN-128 with a sharp increase of cells associated in the first 6 h, which continues to rise until 24 h. In comparison, PCN-128@PEG–PO_3_ shows a much slower increase in cellular affinity over time until 24 h, where the percentage of cells associated with PCN-128@PEG–PO_3_ remains the same afterwards. This effect is less profound in PCN-222, where cell affinities for bare PCN-222 and PCN-222@PEG–PO_3_ at 24 h were similar. Cellular uptake of nanoparticles can be influenced by many properties of the nanoparticle, such as size, shape, charge, and surface chemistry. Both bare PCN-128 and PCN-222 are rod-shaped nanoparticles with similar charge and particle size, but their linkers constitute different external surfaces on the nanoparticle.^67^ This could subsequently lead to different interfacial interactions between bare nanoMOFs and proteins,^68,69^ which may possibly account for the extremely high cellular association for bare PCN-128 but not bare PCN-222 in Figure 7. Although PEGylation of nanoparticles under in vitro conditions has been reported to show reduced cellular uptake,^66,70^ the exact reason currently remains unclear. After PEGylation, nonspecific protein adsorption could be minimized,^71^ and in our case, the zeta potential for PEGylated particles changes from a positively to a negatively charged surface (Figures 1d and S42), which may also explain the differences in cellular affinity due to the presence of negatively charged macromolecules at the cell surface.^72^ To further assess whether these nanoMOFs are internalized into cells or are on the external surface, we performed internalization studies by treating HeLa cells with either bare or PEGylated nanoMOFs for 24 h (Figure S52). We utilized the optical sectioning capabilities of the laser scanning confocal microscope to reconstruct 3D images, which reveal the localization of nanoMOFs in the interior of the cells. Figure 7b shows orthogonal projections and 3D visualizations of *z*-stack images of cells counterstained with wheat germ agglutinin (WGA, colored in white) to outline their plasma membranes and the nanoMOFs (displayed in violet). Both bare PCN-128 and bare PCN-222 are internalized and accumulated as dim clusters inside the cells. Notably, we also observed some bare PCN-222 in large clusters located at the surface of the cells and in the extracellular space on the coverslip, suggesting that bare PCN-222 aggregated in these locations upon exposure to complete cell medium during the 24 h incubation. Indeed, this is consistent with our previous findings where we showed that bare MOFs exhibit shorter term dispersity and colloidal stability than PEGylated ones (Figure S43). In comparison, the internalized PCN-128@PEG–PO_3_ and PCN-222@PEG–PO_3_ appear as large bright particles in the cytoplasm as well as some diffused particles inside the cells (Figure 7b).

### In Vitro Cytotoxicity of Free DOX, DOX@PCN-128, and DOX@128@PEG–PO_3_

**Figure 8:**
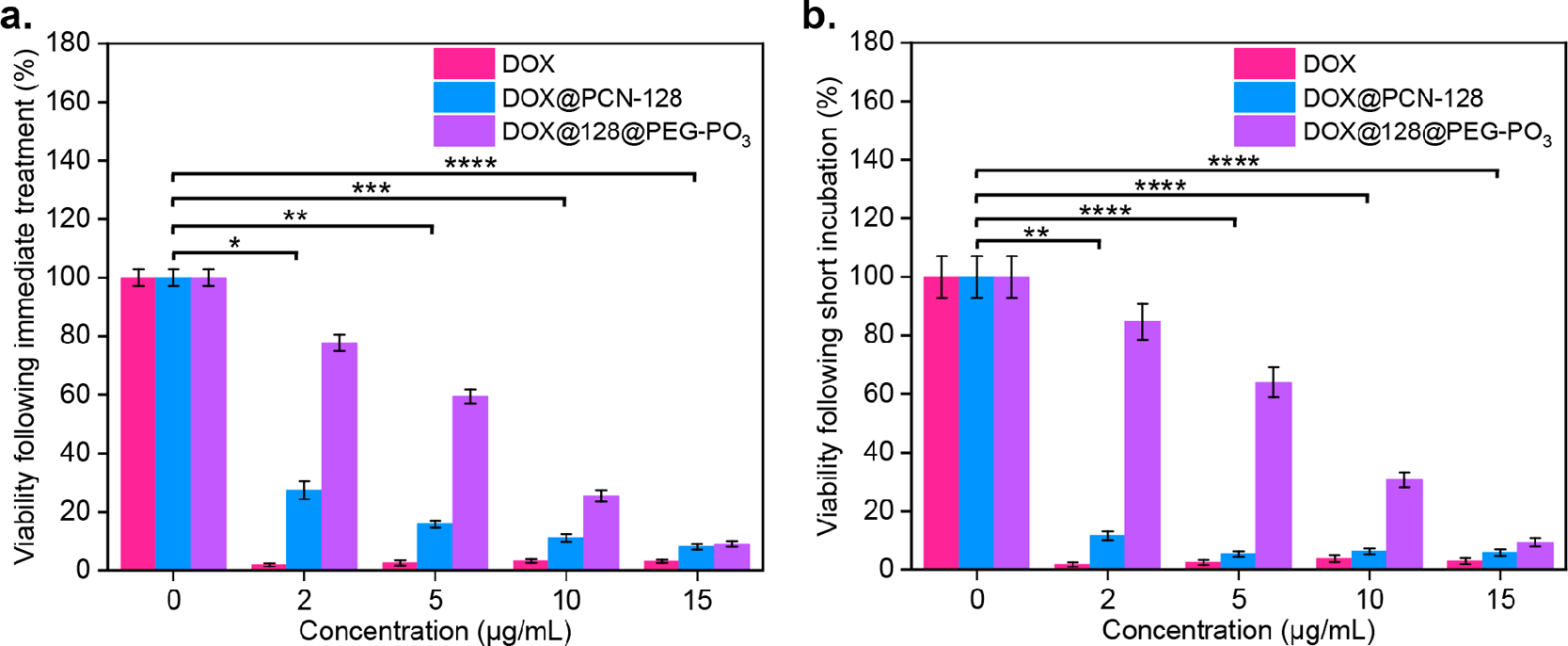
Cytotoxicity of free DOX, DOX@PCN-128, and DOX@128@PEG–PO_3_. HeLa cells’ viability was measured by MTS assay after a 72 h treatment of free DOX, DOX@PCN-128, and DOX@128@PEG–PO_3_. (a) Freshly prepared stock solutions. (b) Stock solution after being kept at room temperature for 2 h (*n* = 4; **** *p* ≤ 0.0001, *** *p* ≤ 0.001, ** *p* ≤ 0.01, * *p* ≤ 0.05).

In order to avoid the overlap of absorbance spectra between DOX and nanoMOFs (Figure S30), we next selected fluorescent PCN-128 to compare the anticancer efficacy between DOX, DOX@PCN-128, and DOX@128@PEG–PO_3_ by MTS assays and IncuCyte for live-cell imaging, which enables observation of cell behavior over time by automatically gathering and analyzing images. Briefly, we prepared free DOX, DOX@PCN-128, and DOX@128@PEG–PO_3_ at the same DOX concentration (1 mg/mL) as a stock solution prior to each experiment, which was either used immediately or incubated at room temperature for 2 h before being used in cell culture. The viability was evaluated by MTS assay after a 72 h treatment. Figure 8 shows that HeLa cells treated with free DOX exhibited significant toxicity over a 72 h incubation even at 2 μg/mL, whereas DOX@PCN-128 and DOX@128@PEG–PO_3_ had lower—but a concentration-dependent trend—toxicity than free DOX with DOX@PCN-128 being more toxic than DOX@128@PEG–PO_3._ This suggests that the presence of mPEG–PO_3_ has a protective effect on the encapsulated drug cargo, thus preventing the burst release, consistent with the release profile (Figure S45c). On the other hand, when a stock solution of DOX@PCN-128 was used 2 h after its preparation (Figure 8b), its cytotoxicity was more profound at lower concentrations than if it was used immediately. In contrast, in the case of DOX@128@PEG–PO_3_, the short incubation period did not cause any significant change in toxicity (Figure 8a). This could be explained by the DOX-release profiles (Figure S45c), where the burst release of DOX@PCN-128 provoked a similar effect to that of free DOX, thereby lessening its capabilities as a controlled-release, drug-delivery vehicle in comparison to the PEGylated counterpart.

**Figure 9:**
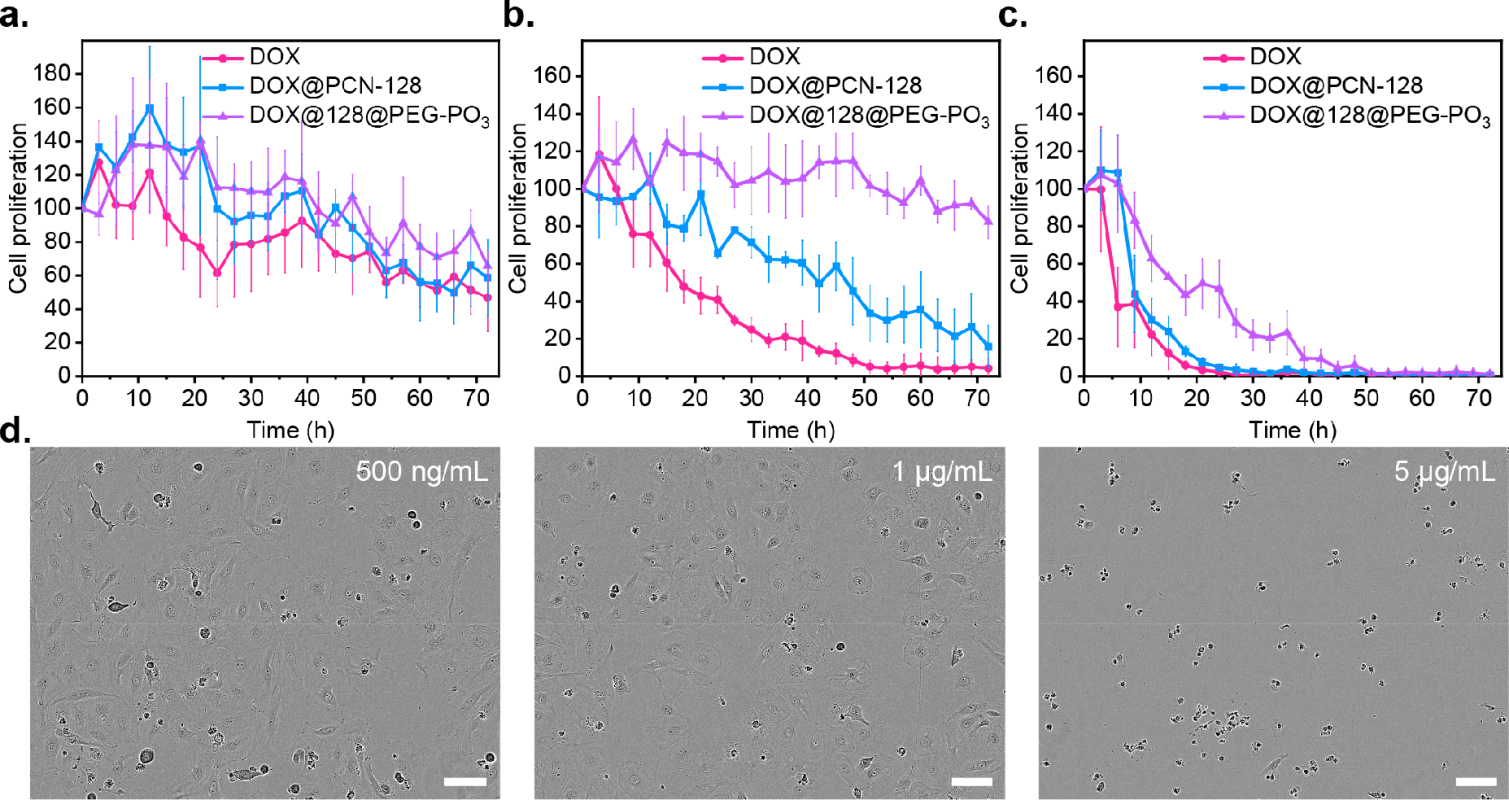
Cell proliferation over a 72 h incubation of DOX, DOX@PCN-128, and DOX@128@PEG–PO_3_. HeLa cells were treated with DOX, DOX@PCN-128, and DOX@128@PEG–PO_3_ and imaged by IncuCyte Zoom every 3 h for a total 72 h incubation. Three different concentrations were used: (a) 500 ng/mL, (b) 1 μg/mL, and (c) 5 μg/mL. (d) Representative phase-contrast photos of HeLa cells treated with DOX@128@PEG–PO_3_ in different concentrations after 72 h (see Videos S8, S9, and S10). Scale bar: 150 μm.

To further examine the effect of released DOX on cell proliferation, we carried out continuous live-cell imaging at 3 h intervals over the course of 72 h on HeLa cells treated with free DOX and DOX-loaded, bare, and PEGylated PCN-128. Figure 9a shows the time-dependent apoptosis of HeLa cells upon treatment with free DOX, DOX@PCN-128, and DOX@128@PEG–PO_3_ at a DOX concentration of 500 ng/mL. HeLa cells proliferated on the first day of treatment, but the cell count decreased over time with no significant differences between each treatment. At a higher DOX dosage of 1 μg/mL (Figure 9b and Videos S8, S9, and S10), DOX reduced HeLa cells by one-half in less than 24 h, while in the case of DOX@PCN-128, 50% cell death was obtained at 48 h. In contrast, cells treated with DOX@128@PEG–PO_3_ were able to proliferate initially on day 1, but some cells seem to struggle later and resulted in cell death. After further increasing the concentration based on DOX to 5 μg/mL (Figure 9c and 9d), both DOX and DOX@PCN-128 performed similarly, causing dramatic cell death in less than 10 h, whereas DOX@128@PEG–PO_3_ was capable of slowing down cell death for 24 h before one-half of the cells were deceased. These results indicate that both PCN-128 and PCN-128@PEG–PO_3_ were capable of delivering their drug cargo DOX and achieve a similar cytotoxic effect at low concentration. Most importantly, PEGylated PCN-128, in particular, had the ability to release encapsulated DOX slowly at a high drug concentration, highlighting its therapeutic potential for controlled release as a drug-delivery vehicle.

## Outlook

We demonstrated the development of a mild and general strategy for the formulation of Zr–MOFs-based drug carriers by performing PEGylation in an aqueous mPEG–PO_3_ solution at room temperature and drying with lyophilization. We performed a series of ex situ characterizations at different reaction times and MD simulations to systematically study the PEGylation process of PCN-222. On the basis of these results, we propose a two-step PEGylation process for PCN-222. First, the mPEG–PO_3_ molecules bond to the available sites at the surface due to the electrostatic interactions between positively charged PCN-222 and negatively charged mPEG–PO_3_; second, after occupying the available binding sites at the surface, the mPEG–PO_3_ molecules start to enter the internal channels of PCN-222 and partially block the porosity. We then extended our formulation strategy to other Zr-based MOFs, which have distinct pore sizes, particle sizes, and morphologies; the obtained PEGylated nanoMOFs exhibited improved colloidal stabilities in water and PBS (pH = 7.4) compared to their parent counterparts. With DOX as a model drug, we tested their application in drug storage. Most importantly, the lyophilized bare or DOX-containing PEGylated nanoMOFs showed excellent redispersibility with sonication treatment while maintaining their hydrodynamic diameters. In vitro studies suggest that the presence of mPEG–PO_3_ greatly reduces the cytotoxicities of nanoMOFs at higher concentrations and avoids the burst release of the loaded DOX. Using flow cytometry and a series of *z*-stack confocal microscopy images, we observed different cellular affinity and internalization of bare and PEGylated nanoMOFs within the HeLa cells. Since the majority of established MOF-based drug-delivery vehicles are kept in wet conditions, this work for the formulation of redispersible drug-containing MOF composites not only represents an easy way to increase the colloidal stability of nanoMOFs and simultaneously endow them with water redispersibility through the use of a phosphate-functionalized PEG but also provides inspiration for the storage of MOFs-based drug carriers as well as new drug-release options, which holds great potential for biomedical applications.

## Supporting Information Available

Side and top view of one mPEG–PO_3_ molecule in PCN-222 (MP4)
Redispersion of PCN-222@PEG–PO_3_ and DOX@222@PEG–PO_3_ in water (MP4)
Redispersion of MOF@PEG–PO_3_ and DOX@MOF@PEG–PO_3_ in water (MP4)
Three-dimensional visualization of *z*-stack confocal images of HeLa cells with PCN-128 turn around Y (MP4)
Three-dimensional visualization of *z*-stack confocal images of HeLa cells with PCN-128@PEG–PO_3_ turn around Y (MP4)
Three-dimensional visualization of *z*-stack confocal images of HeLa cells with PCN-222 turn around Y (MP4)
Three-dimensional visualization of *z*-stack confocal images of HeLa cells with PCN-222@PEG–PO_3_ turn around Y (MP4)
IncuCyte Live cell imaging of HeLa cells treated with DOX over 72 h (MP4)
IncuCyte Live cell imaging of HeLa cells treated with DOX@PCN-128 over 72 h(MP4)
IncuCyte Live cell imaging of HeLa cells treated with DOX@128@PEG–PO_3_ over 72 h (MP4)
Materials synthesis, characterization details, supporting data items, and supporting figures (PDF)
