# Supplementary material for: Formulation of Metal–Organic Framework-Based Drug Carriers by Controlled Coordination of Methoxy PEG Phosphate: Boosting Colloidal Stability and Redispersibility
Source: J Am Chem Soc. 2021 Aug 6;143(34):13557–72. doi: 10.1021/jacs.1c03943 (PMC8414479; doi:10.1021/jacs.1c03943)
Supplement: Supplementary file 11 — ja1c03943_si_011.pdf [file ja1c03943_si_011.pdf]

Supporting Information for:

Formulation of Metal-Organic Frameworks-based Drug Carrier by Controlled Coordination of Methoxy PEG Phosphate: Boosting Colloidal Stability and Redispersibility

Xu Chen[a], Yunhui Zhuang[a], Nakul Rampal[a], Rachel Hewitt[e], Giorgio Divitini[b], Christopher A. O'Keefe[c], Xiewen Liu[a], Daniel J. Whitaker[c], John W. Wills[e], Ravin Jugdaohsingh[e], Jonathan J. Powell[e], Han Yu[d,*], Clare P. Grey[c], Oren A. Scherman[c] and David Fairen-Jimenez[a,*]

[a]Adsorption & Advanced Materials Laboratory (A^2ML), Department of Chemical Engineering & Biotechnology, University of Cambridge, Philippa Fawcett Drive, Cambridge CB3 0AS, U.K.

[b]Electron Microscopy Group, Department of Materials Science and Metallurgy, University of Cambridge, 27 Charles Babbage Road, Cambridge CB3 0FS, U.K.

[c]Department of Chemistry, University of Cambridge, Lensfield Road, Cambridge CB2 1EW, U.K.

[d]School of Chemical and Environmental Engineering Shanghai Institute of Technology, No. 100 Haiquan Road, Shanghai 201418, P.R. China

[e]Biominerals Research Laboratory & Cellular Imaging and Analysis Facility, Department of Veterinary Medicine, University of Cambridge, Madingley Road, Cambridge CB3 0ES, UK

*e-mails: hanyu0220@tsinghua.edu.cn; df334@cam.ac.uk

Table of Contents

**S1.**	General experimental remarks	**S2**
**S2.**	General synthetic procedures	**S8**
**S3**	Characterizations	**S13**
**S4**	BET area calculation using BETSI	**S43**
**S5.**	MD and GCMC simulations	**S53**
**S6.**	*In Vitro* evaluation of nanoMOFs	**S56**
**S7.**	References	**S61**

S1. General experimental remarks

Lyophilization was carried out using a Telstar LyoQuest benchtop freeze dryer (0.008 mBar, -70 °C).

Powder X-ray Diffraction (PXRD): PXRD data were collected on a Bruker D8 DAVINCI diffractometer at 298 K using Cu K*α* radiation. The calculated PXRD patterns were produced using the Mercury program and single crystal reflection data.

Thermogravimetric Analysis (TGA): TGA measurements were carried out using a TA Instruments Q500 Thermogravimetric Analyzer. Measurements were collected from room temperature to 800 °C with a heating rate of 5 °C / min under nitrogen.

Fourier-transform Infrared Spectroscopy (FT-IR): FT-IR was carried out using a Bruker Tensor 27 FTIR with attenuated total reflectance (ATR) method.

Gas Uptake: N_2 adsorption isotherm measurements were performed on a Micromeritics 3-Flex analyzer at 77 K. Around 40 mg samples were used for each measurement, Prior to the measurement, all the samples were degassed under vacuum at 55 °C for 20 hours using the internal turbopump. 55 °C was selected as the activation temperature to avoid further insertion of the PEG caused by thermal annealing,[1] Gas uptakes were performed on the air-dried samples.

Dynamic Light Scattering (DLS) and Zeta Potential: Measurements were recorded in aqueous solution with the sample concentration of 0.2 mg/ mL by a Zetasizer Nano ZS, (Malvern Instrument Ltd., U.K.) equipped with a He−Ne laser operating at 633 nm at 25 °C. Zeta potential was measured in a folded capillary Zeta cell DTS1070. The Smoluchowski equation was used to calculate the zeta potential. Measurements were performed three times with over 10 subruns for each sample. Error bars represent the standard deviation of three measurements.

Inductively Coupled Plasma-optical Emission Spectroscopy (ICP-OES): ICP-OES was performed using a Perkin Elemer ICP-OES Optima 2100DV. Samples were dispersed in 2 mL of nitric acid and 6 mL of hydrochloric acid (*CAUTION!*) and left to stand at room temperature in the fume cupboard for at least 1 h until all reactions have ceased. After that, samples were heated at 90 °C for 10 h to fully digest the sample. The mixture was diluted 750 times before the measurement.

X-ray Photoelectron Spectroscopy (XPS): XPS analysis was carried out using a Thermo Fisher Scientific ESCALAB 250Xi XPS System. A monochromatic Al-Kα source (1486.74 eV) and a charge neutralizer were used for all samples. Survey scans were acquired using pass energy of 100 eV, 1 scan and dwell time of 50 ms. High-resolution data were acquired using 50 eV pass energy, 50

scans, dwell time of 50 ms and standard magnetic lenses.

UV-Vis Spectroscopy: UV-vis and fluorescence spectra were recorded using a Tecan Spark® Multimode Microplate Reader.

Liquid Nuclear Magnetic Resonance Spectroscopy (NMR) was carried out using a Bruker 400 MHz Avance III HD Smart Probe Spectrometer.

Solid-State Nuclear Magnetic Resonance Spectroscopy (NMR): Solid-state NMR experiments were conducted on a Bruker Avance III 200 MHz spectrometer equipped with a 4.7 T magnet (corresponding to Larmor frequencies: $v_0(^1H)$ = 200 MHz and $v_0(^{31}P)$ = 81.0 MHz). A Bruker 1.3 mm double-channel magic-angle spinning (MAS) probe was used with a spinning frequency of 60 kHz. Spectra were acquired using a rotor-synchronized Hahn echo experiment with optimized excitation and refocusing pulse lengths of 1.56 and 3.12 µs, respectively. A recycle delay of 1 s was used for all experiments, and either 4096 (mPEG-PO_3 sample) or 8192 (MOF sample) transients were collected. Spectra were processed with 50 Hz of exponential apodization, zero filling, followed by Fourier transformation. ^{31}P chemical shifts were calibrated using 85% H_3PO_4 in H_2O ($\delta_{iso}(^{31}P)$ = 0 ppm) as an external reference.

Scanning Electron Microscopy (SEM): The samples for SEM tests were coated with Pt or Au for 40 seconds and imaged using a FEI Nova Nano SEM 450.

Transmission Electron Microscopy (TEM): The samples for TEM test were prepared by dispersing the samples in ethanol using ultrasonication. After that, a small number of suspensions were drop-casted on a copper grid with a carbon support film. TEM micrographs were collected on a Tecnai F20 with an acceleration voltage of 200 kV. Scanning transmission electron microscopy (STEM) was carried out on a FEI Tecnai Osiris operated at 200 kV using a OneView CMOS camera. The EDX chemical maps were acquired using a Super-X detector setup, with a total acquisition solid angle of 0.9 sr.

Cryogenic Electron Microscopy (cryo-EM): The samples were prepared for cryo-EM using a Vitrobot and conventional vitrification protocols (blot total 1, blot force 3, blot time 2.5 s). The frozen samples on Quantifoil grids were imaged in a Krios cryo-EM operated at 300 kV, equipped with a Falcon4 detector. The images were acquired using EPU using a Falcon4 detector in counting mode.

Size Exclusion Chromatography with Multi-angle Light Scattering (SEC-MLAS) was performed on a Shimadzu HPLC system consisting of an LC-20AD Pump, SIL-20A autosampler, CTO-20A column oven and CBM-20A control unit. The column set was made up of 1 x PSS SUPREMA

analytical 100 Å (8 x 300 mm) and 2 x PSS SUPREMA analytical 3000Å (8 x 300 mm). Dual detection was achieved *via* a Wyatt DAWN HELEOS-II multiangle light scattering (MALS) detector (laser at λ = 658 nm), and Wyatt Optilab rEX differential refractive index (DRI) detector with a 658 nm light source. MilliQ Water containing 0.1 mol/L sodium nitrate and 0.01 mol/L sodium azide was used as the eluent at a flow rate of 1.0 mL/min. The column temperature and the detector temperature were kept at 30 °C. All data analysis was performed using Wyatt Astra V 6.1.1 software. A literature value for the dn/dc of poly(ethylene glycol) in water (0.134 mL/g)[2] was used to determine the molecular weight of all samples.

Redispersing of PEGylated nanoMOF was carried out using a ultrasonic sonicator bath (2 min, room temperature). Particle size distributions measured with DLS after low-speed centrifugation (1 min, 1000 rpm). For the convenience of observation, the process of redispersion, we used a probe sonicator (Qsonica Q125 sonicator with a 3 mm diameter probe) instead. Briefly, to a 20 mL vial, MOF@PEG-PO_3 or DOX@MOF@PEG-PO_3 (20 mg) and H_2O (10 mL) were added. The mixture was sonicated using a probe sonicator (20% amplitude) at room temperature for 2 min, followed by standing for another 5 min. See **Videos S2** and **S3** in the Supporting Information.

Cell Culture: HeLa cells were chosen as our *in vitro* cellular system to evaluate the biocompatibility and cellular uptake efficiency of our nanoMOFs, as well as their capacity as drug delivery system. HeLa cells were maintained at 37 °C and 5% CO_2 in high rich glucose (4500 mg/L) DMEM (Gibco Dulbecco's Modified Eagle Medium, Gibco® 41965039) supplemented with 10% (v/v) fetal bovine serum (Sigma-Aldrich F9665), 100 units/mL penicillin, and 100 µg/mL streptomycin (Life Technologies 15140122). The supplemented medium is then named as ‘complete’ medium. PBS (Sigma D8537) and trypsin-EDTA (Life Technologies 25300054) were used to maintain the cell line.

MTS Cytotoxicity Assay: The concentration-dependent viability of PCN-128, PCN-128@PEG-PO_3, PCN-222, PCN-222@PEG-PO_3, DOX, DOX@PCN-128, DOX@128@PEG-PO_3 was investigated using the CellTiter 96® Aqueous Non-Radioactive Cell Proliferation Assay (Promega, UK). Briefly, HeLa cells were seeded on a 96-well plate at a density of 5000 cells/well for approximately 24 h. Prior to each experiment, suspensions of PCN-128 *vs* PCN-128@PEG-PO_3, PCN-222 *vs* PCN-222@PEG-PO_3 (based on the nanoMOFs concentration) and DOX@PCN-128 and DOX@128@PEG-PO_3 (at a concentration of 1 mg/mL based on DOX) were freshly prepared before they were aliquoted in complete medium. Among them, suspensions of PCN-128@PEG-PO_3, PCN-222 *vs* PCN-222@PEG-PO_3 DOX@128@PEG-PO_3 was prepared by redispersing the lyophilized samples in water. Then, nanoMOFs and drugs were dispersed in complete medium and a range of concentrations was prepared accordingly, of which 100 µL were added to each well and incubated for 72 h at 37 ºC, 5% CO_2. At the end of the incubation period for 72 h, the treatment solutions were removed, and cells were washed once with PBS. A solution of 100 µL of complete media and 20 µL

of MTS solution was added to the wells, and the contents were incubated for 1.5 h at 37 °C/5% CO_2. After the incubation, 100μL of the resulting solution was transferred to a clean 96-well plate, and the absorbance was recorded by UV-Vis SPECTROstar Nano at 490 nm.

Flow Cytometry: HeLa cells were seeded on to a 6-well plate the day before they were treated with nanoMOFs at the nanoMOFs concentration of 10 μg/mL to each well. At the end of the incubation period, cells were gently lifted from the plate using 500 μL/well of cell dissociation solution (Thermo Fisher Scientific, 13151014)) for 15 min at 37 °C/5% CO_2. The cell pellets were then collected and washed with PBS by centrifugation and stained with Live Dead stain (Thermo Fisher Scientific, L34955) on ice for 20 min according to the manufacturers' protocol. Following the staining, samples were washed with flow cytometry staining buffer (FACS buffer) and fixed on ice with 2% paraformaldehyde (PFA) for immediate acquisition on the same day. Unstained and single stain compensation tubes, with and without nanoMOFs treatment, were also prepared for spectral overlap compensation.
The CyAn ADP flow cytometer (Beckman Coulter, Ltd, High Wycombe, UK) is equipped with three lasers (405, 488 and 642 nm) in standard configuration and standard filter sets. Summit software v4.3 was used for acquisition and analysis (Beckman Coulter). Samples were filtered through 35 μm nylon cell strainer mesh tubes (BD Biosciences) and 50,000 events were acquired for each sample.

Confocal Microscopy: HeLa cells were grown on 13 mm coverslips within a 24-well plate at 1.5 × 10^4 cells/well density for incubation overnight in complete medium at 37 °C/5% CO_2. Cells were then treated with 100 μg/mL of nanoMOFs diluted in media for 6 h or 24 h incubation. At the end of cellular incubations with or without MOFs, fluorescence labelling was carried out within the cell culture plate wells. In brief, cells were washed 3 times with PBS (5 min each). They were then fixed with 4% paraformaldehyde (PFA) for 10 min, then washed 3 times (1 min each) in Tris-buffered saline (TBS). Following washing, fixed cells were stained with Wheat Germ Agglutinin (WGA) Alexa Fluor 555 conjugate (Thermo Fisher Scientific, W32464) for 15 min in TBS to visualize cell membranes. After 3 final washes in TBS, coverslips with cells attached were carefully removed from tissue culture plate wells and mounted on microscope slides using approximately 40 μL mounting medium (Thermo Fisher Scientific, P36980).
Confocal imaging was carried out using a Zeiss LSM780 laser scanning confocal microscope. The 405 nm and 514 nm lasers were used to excite MOFs and WGA 555 stains, respectively. Images were collected using an oil immersion 63X/1.4NA lens. Zen software (Zeiss) was used for the acquisition image processing.

IncuCyte® Proliferation Assays: HeLa cells were seeded onto 96-well plates at a density of 1500 cells per well and incubated at 37 °C/5% CO_2 for approximately 24 h. Then DOX, DOX@PCN-128, DOX@128@PEG-PO_3 samples were freshly prepared and aliquoted in complete cell medium. Cells

were then cultured and imaged within the IncuCyte Zoom (Essen BioScience) every 3 h using phase-contrast imaging with no lasers on. Images were exported and cells were semi-automatically quantified using ImageJ to find maxima with a range of noise tolerance level from 100-170. Finally, all images are compiled as image sequences to be exported as videos. See **Videos S8-S10** in the Supporting Information.

Molecular Dynamics (MD) Simulations: The structure of PCN-222 is taken from the work of Feng et al.[3] The structure of the mPEG-PO_3 (consisting of 128 monomer units) is created in Material Studio. Geometry optimization of both the polymer and the framework is performed using the Forcite Module in Materials Studio. Charges for the framework and the PEG are calculated using the EQeq protocol.[4] All simulations are performed using the LAMMPS[5] molecular dynamics package using the Universal Force Field (UFF)[6] parameters to describe the Lennard-Jones, bond, angle, dihedral and improper torsion potentials. The simulation box consists of a 2x2x2 cell of PCN-222 – which is considered large enough to avoid any finite-size effects – and one mPEG-PO_3 chain placed at the external surface of the framework. Periodic boundary conditions are applied in all three dimensions. Initially, the energy minimization (equilibration run) of the model is done using the NVE ensemble until a temperature of 298 K is reached. Next, the production runs are conducted using the canonical ensemble (NVT) for a duration of 1.5 ns. All MD simulations are done using a time step of 1 fs. The temperature is maintained using a Nosé-Hoover thermostat[7] with a damping factor of 0.1 ps. The long range electrostatic interactions are computed using Ewald summation and the precision is set to 1×10^{-4}. The non-bonded van-der Waals interactions are computed using the Lennard-Jones potential and the short range electrostatic interactions are computed using the coulombic potential, both with a cut off of 12.5 Å. The trajectories are sampled every 0.01 ns and are viewed using VMD.[8]

Grand canonical Monte Carlo (GCMC) simulations: DOX has been modeled as a flexible model, except for the rings which are kept fixed.[9] The bonding, bending, and torsional parameters have all been taken from the OPLS-AA force field.[10] **Table S5** shows the atom numbers assigned to the different atoms of the molecule, with their corresponding OPLS-AA atom types, along with their Lennard-Jones parameters, and charges. The loading of DOX at 1 atm and 298 K is simulated using the GCMC method as implemented in the RASPA simulation package.[11] Monte Carlo moves consist of insertion, deletion, translation, and rotation moves, each taking place with equal probabilities. We use 20,000 cycles for equilibration and 200,000 cycles to average the properties. A cycle is defined as the maximum of 20 steps or the number of molecules in the system; this means that on average, a Monte Carlo move has been attempted on all the molecules during each cycle. The crystallographic information file (cif) of the different frameworks have been taken from the Cambridge structural database (CSD).[12] All the framework atoms are kept fixed at the crystallographic positions with periodic boundary conditions applied in all directions to make sure that the simulation box is large enough to ensure that a distance of at least twice the cutoff radius is maintained between the periodic

images. Intermolecular interactions are modeled using the Lennard-Jones (LJ) potential with a cutoff of 12.8 Å. Lorentz-Berthelot mixing rules are used for all the cross interaction terms. Electrostatic interactions are modeled using the coulombic potential and are computed using the Ewald summation method with the precision set to 10^{-6}. The partial charges for the framework are calculated using the EQeq protocol.[4] The framework atoms are modeled using LJ parameters taken from the DREIDING force field.[13] For Zr, which is not described in the DREIDING force field, the LJ parameters are taken from the Universal force field (UFF).[6] **Table S6** shows the LJ parameters for the atoms of the framework. Maximum DOX loadings (in wt%) obtained from the GCMC simulations are listed in **Table S4** with the corresponding snapshots showing the DOX (in green) loading within the framework shown in **Figures S47** (MOF-808)**, S48** (NU-901)**, S49** (PCN-128)**,** and **S50** (PCN-222).

S2. General synthetic procedures

All reagents unless otherwise stated were purchased from Sigma Aldrich, Fluorochem or Alfa Aesar and used as provided. The synthesis of Zr_6 cluster, tetrakis (4-carboxyphenyl)porphyrin (TCPP) and (4', 4''', 4''''', 4''''''-(ethene-1,1,2,2-tet-rayl)tetrakis(([1,10-biphenyl]-4-carboxylate) (ETTC) were adapted from the literature procedures.[3, 14-15] Milli-Q water (18.2 MΩ.cm resistivity at 25 °C) was used throughout the experiment. Dialysis tubing (Molecular Weight Cut-off; MWCO 3,500 and12,000 - 14,000 Daltons) was obtained from Medicell Membranes Ltd.

Synthesis of mPEG5K-phosphate (mPEG-PO_3)

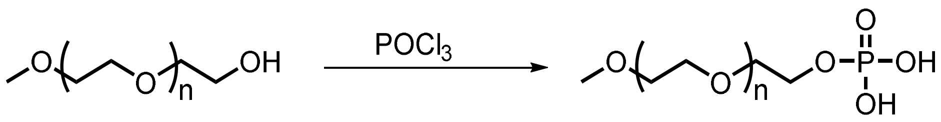

Scheme S1. Synthesis of mPEG-PO_3.

mPEG-PO_3 was synthesized according to a previously reported method with slight modification.[16] To a flame-dried two-neck flask, phosphorus oxychloride ($POCl_3$) in (100 mmol) 10 mL of dry DCM was added, followed by the addition of dry triethylamine (300 mmol) at 0 °C. After that, poly(ethylene glycol) methyl ether (M_n = 5000, 30 mmol) was added to the mixture in 100 mL of dry DCM over 30 min. The resulting solution was stirred at room temperature for 10 h, then 40 mL of water was added, the mixture was reacted for an additional 3 h. DCM was removed under a vacuum, and the raw product was purified by dialysis (MWCO 3,500) followed by removing the residual water with lyophilization to give **mPEG-PO_3** as a white solid (127.5 g, 25.5 mmol, 85%). ^{1}H NMR (D_2O, 400 MHz), δ: 3.63 (s, 448H, -C**H**$_2$OC**H**$_2$-), 3.31 (s, 3H, -OC**H**$_3$) ^{31}P NMR δ: (D_2O, 162 MHz) δ: 0.22.

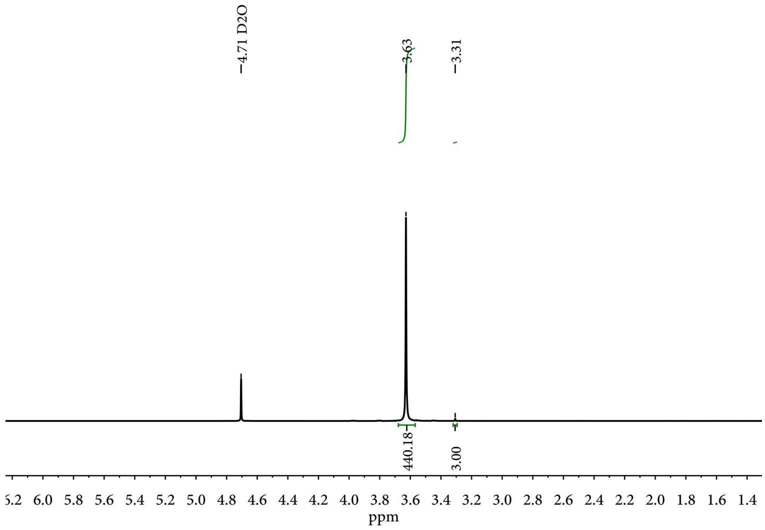

Figure S1. ^{1}H NMR spectrum of mPEG-PO_3.

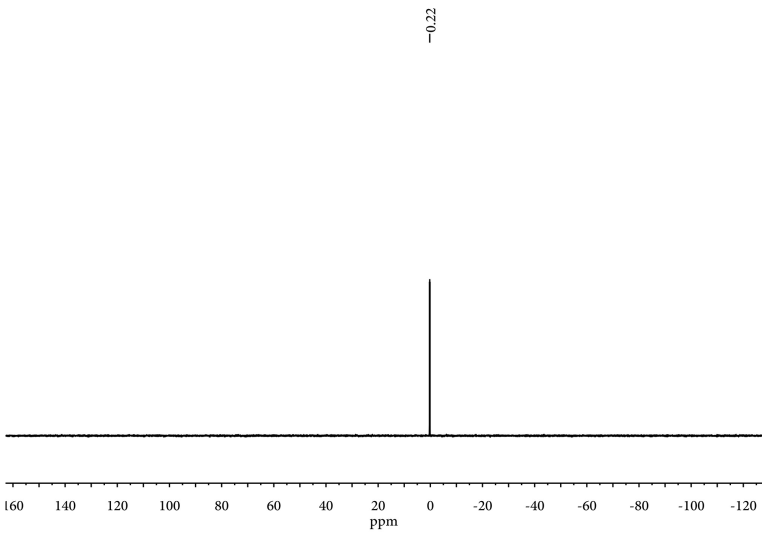

Figure S2. ^{31}P NMR spectrum of mPEG-PO_3. Only one singlet was observed in ^{31}P NMR, indicating that either one or two PEG chains is/are attached to the phosphate group, whereas, M_n obtained from GPC (Figure S3) further demonstrated the presence of one PEG chain.

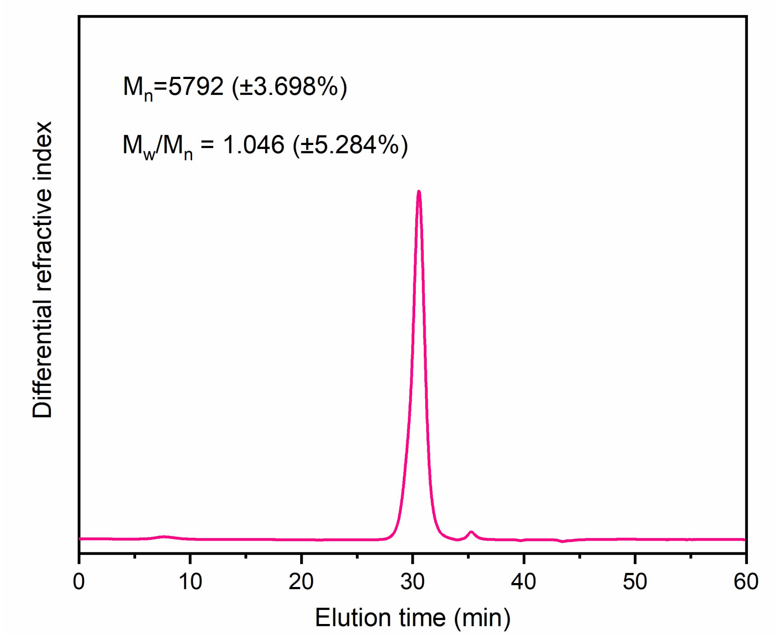

Figure S3. GPC trace of mPEG-PO_3 (5k).

Size-Controlled Synthesis of PCN-222 Nanoparticles

To control the size of PCN-222, different contents of trifluoroacetic acid (TFA) were utilized. The details are as follows:

TCPP (22.5 mg, 28.5 μmol), Zr_6 cluster (38 mg, 14.2 μmol), 8 mL DMF and **a μL of TFA** were ultrasonically dissolved in a 20 mL threaded vial. The resultant mixture was placed in the 120 °C block heater for 5 hours. After cooling down to room temperature, the obtained purple sample was collected by high-speed centrifugation (15,000 rpm, 35 min), followed by washing with fresh DMF 3 times and exchanging with ethanol 3 times. The final product was redispersed in ethanol for further use. (a = 80, 130, 180 and 400 μL, the related size = 83.7 ± 9.8 nm, 117.8 ± 12.9 nm, 266.1 ± 22.6 nm and 628 ± 36.6 nm).

Synthesis of PCN-222 (Hf)

TCPP (10 mg, 12.6 μmol), $HfOCl_2·8H_2O$ (25 mg, 62.1 μmol), 8 mL DMF and 200 μL TFA were ultrasonically dissolved in a 20 mL threaded vial. The resultant mixture was placed in the 120 °C block heater for 5 hours. After cooling down to room temperature, the obtained purple sample was collected by high-speed centrifugation (15,000 rpm, 35 min), followed by washing with fresh DMF 3 times and exchanging with ethanol 3 times. The final product was redispersed in ethanol for further use.

Synthesis of UiO-66 (166.7 ± 30.2 nm)

1,4-benzenedicarboxylic acid (BDC) (320 mg, 1.93 mmol), $ZrCl_4$ (466 mg, 2.00 mmol), benzoic acid (2.44 g, 20 mmol) and 36 mL DMF were ultrasonically dissolved in a 100 mL threaded vial, followed by addition of concentrated HCl (0.33 mL). The mixture was heated at 120 °C for 48 h. After cooling to room temperature, the obtained sample was collected by centrifugation (4,700 rpm, 35 min), followed by washing with fresh DMF 3 times and exchanging with ethanol 3 times. The final product was redispersed in ethanol for further use.

Synthesis of MOF-808 (44.7 ± 25.0 nm)

1,3,5-benzenetricarboxylic acid (70 mg, 0.33 mmol), $ZrCl_4$ (233 mg, 1.00 mmol), formic acid (5.6 mL) and DMF (10 mL) were ultrasonically dissolved in a 50 mL threaded vial. The mixture was heated at 120 °C for 48 h. After cooling to room temperature, the obtained sample was collected by high-speed centrifugation (15,000 rpm, 35 min), followed by washing with fresh DMF 3 times and exchanging with ethanol 3 times. The final product was redispersed in ethanol for further use.

Synthesis of NU-901 (251.2 ± 47.1 nm)

1,3,6,8-tetrakis(*p*-benzoic acid)pyrene (H_4TBAPy) (25 mg, 40.4 μmol), $ZrOCl_2·8H_2O$ (121 mg, 375.5 μmol), 4-aminobenzoic acid (400 mg, 2.92 mmol), TFA (200 μL) and DMF (20 mL) were ultrasonically dissolved in a 50 mL threaded vial. The mixture was heated at 140 °C for 50 min, whereupon a yellow suspension formed. After cooling to room temperature, the obtained sample was collected by centrifugation (4,700 rpm, 35 min), followed by washing with fresh DMF 3 times and exchanging with ethanol 3 times. The final product was redispersed in ethanol for further use.

Synthesis of PCN-128 (180.9 ± 27.1 nm)

ETTC (10 mg, 12.3 μmol), Zr_6 cluster (20 mg, 7.5 μmol), DMF (4 mL) and TFA (100 μL) were ultrasonically dissolved in a 6 mL threaded vial. Afterwards, triethylamine (TEA) (20 μL) was added. The mixture was heated at 120 °C for 24 h. After cooling to room temperature, the obtained sample was collected by high-speed centrifugation (15,000 rpm, 35 min), followed by washing with fresh DMF 3 times and exchanging with ethanol 3 times. The final product was redispersed in ethanol for further use.

The synthesis of PCN-222@PEG-PO_3

mPEG-PO_3 solution (10 mL, 25 mg/mL in H_2O) was added into the aqueous suspension of PCN-222 (5 mL, 10 mg/mL). After stirring at room temperature for 16 h, the reaction mixture was centrifuged to remove the unreacted mPEG-PO_3, and redispersed in water, then dialyzed (MWCO, 12,000~14,000) against water for 12 h. The water was decanted and replenished with the fresh one every 4 h. The obtained suspension of PCN-222@PEG-PO_3 was then lyophilized (0.008 mBar, -70 °C) to give a brown product, or collected by high-speed centrifugation (18,000 rpm, 35 min), washed with ethanol and dried under the air, giving a dense and dark coloured product.

The methods for synthesis of UiO-66@PEG-PO_3, MOF-808@PEG-PO_3, NU-901@PEG-PO_3 and PCN-128@PEG-PO_3 were similar to that of PCN-222@PEG-PO_3. The amount of mPEG-PO_3 was determined by ICP-OES (P/Zr). The amount of mPEG-PO_3 (wt %) = mass of encapsulated mPEG-PO_3/MOF@PEG-PO_3 × 100.

The time-dependent study of the PEGylation of PCN-222

To 4 identical vials containing 5 mL water suspension of 120 nm-sized PCN-222 (10 mg/mL) were added with 10 mL mPEG-PO_3 solution (25 mg/mL in H_2O), respectively, the mixture was stirred (800 rpm) at room temperature for 2, 4, 12 and 16 h. At each time point, one sample was quenched by centrifugation to remove the unreacted mPEG-PO_3, and washed with fresh water 3 times, then redispersed in water. The obtained suspension was lyophilized (0.008 mBar, -70 °C) or collected by high-speed centrifugation (15,000 rpm, 35 min), washed with ethanol and dried under the air, giving the desired products, named PEGylation *x* h (*x* is the reaction time). The lyophilized sample was used for PXRD, EM imaging, while the air-dried sample was used for N_2 adsorption measurements.

DOX encapsulation

DOX@MOF; The aqueous as-synthesized nanoMOFs (5 mg/mL, 6 mL) suspension was added to an aqueous DOX solution (15 mg/mL, 4 mL), the mixture was then stirred for 48 hours at room temperature. Afterwards, the DOX loaded nanoMOFs were collected by centrifugation (15,000 rpm, 35 min) and washed with water for 3 times. The unloaded DOX was combined and diluted to a final volume of 500 mL. The obtained DOX@MOF was dried under vacuum or redispersed in water for future use.

DOX@MOF@PEG-PO_3; The aqueous as-synthesized nanoMOFs (5 mg/mL, 6 mL) suspension was added to an aqueous DOX solution (15 mg/mL, 4 mL), the mixture was then stirred for 48 hours at room temperature. Afterwards, the aqueous mPEG-PO_3 solution (6 mL, 25 mg/mL) was added, the resulting mixture was stirred for another 16 h. The reaction mixture was then centrifuged (15,000 rpm, 35 min) to remove the unreacted mPEG-PO_3 and unloaded DOX, and redispersed in water, followed by dialysis (MWCO, 12,000~14,000) against water for 12 h. The water was decanted and replenished with the fresh one every 4 h. The unloaded DOX was combined and diluted to a final volume of 500 mL. The final suspension was lyophilized (0.008 mBar, -70 °C) or dried under vacuum.

The absorbance was determined by UV-Vis spectroscopy (maximum absorbance at 486 nm). The concentration was calculated by comparing the UV-Vis absorbance with a calibration curve (**Figure S31**).

Drug loading (wt %) = (mass of drug used - mass of unloaded drug) / (mass of MOF + loaded drug) × 100.

Drug release

In PBS (pH = 7.4): Around 10 mg DOX@MOF and DOX@MOF@PEG-PO_3 were suspended in 2 mL PBS (pH = 7.4) and placed in dialysis bags (MWCO; 1,2000 – 1,4000) dispersed with 2 mL PBS (pH = 7.4), dialyzing against the same media (28 mL) under magnetic stirring at room temperature. 1 mL of the media was taken out and replaced with fresh media at specific time intervals.

The drug released was analyzed by fluorescence spectroscopy by comparing with their calibration curves (**Figure S32**, emission maximum at 595 nm when excited at 486 nm).

Stability study

DOX-loaded bare and PEGylated nanoMOFs were dispersed in PBS (pH = 7.4) or water, samples nanoparticles were characterized by TEM and PXRD at different intervals of time.

S3. Characterizations

Optical and EM images

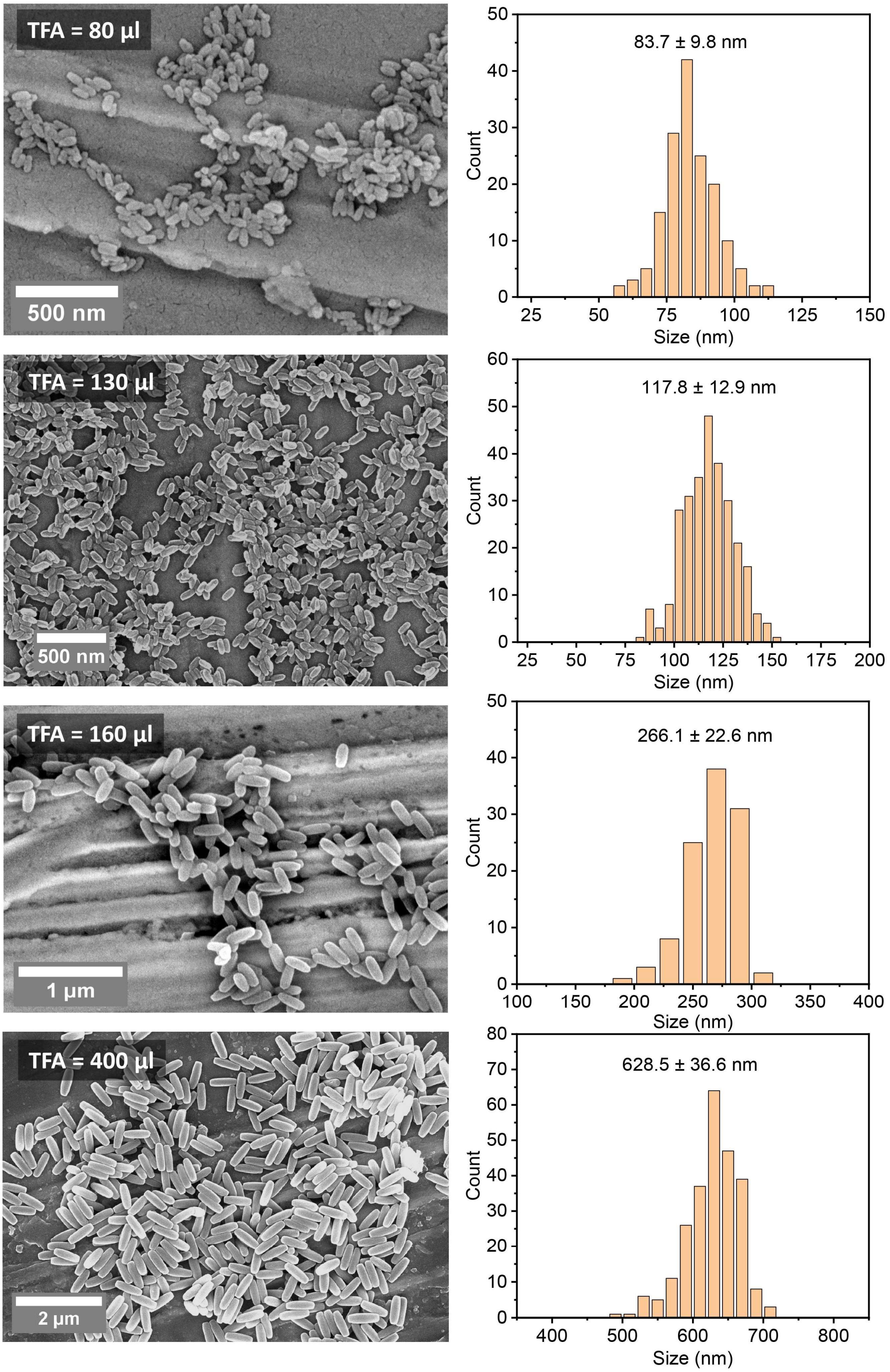

Figure S4. SEM images and particle size analysis of PCN-222. Average particle sizes were determined by manually counting at least 100 particles.

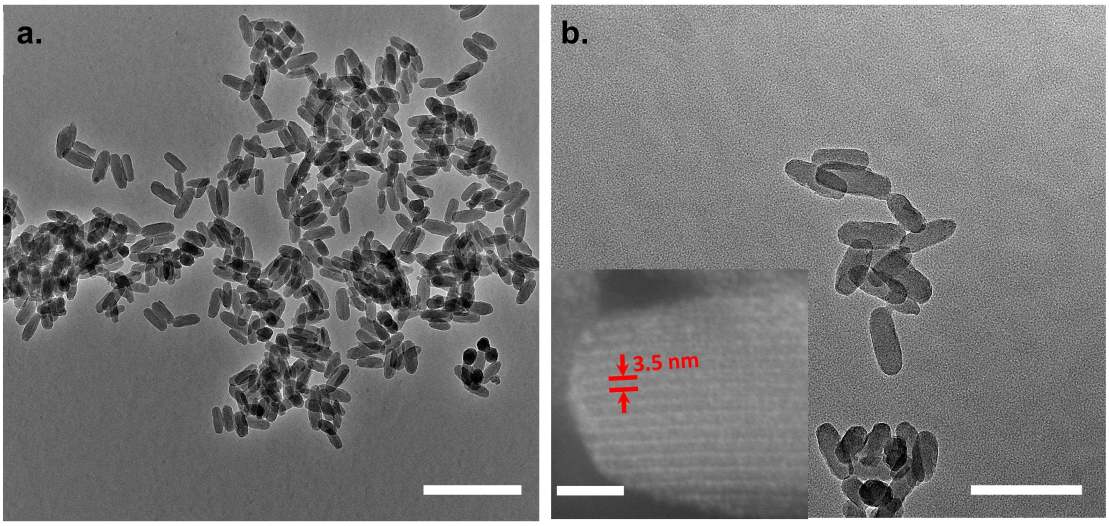

Figure S5. a. TEM and **b.** HAADF-STEM (inset) images of PCN-222. Scale bar, 500 nm (left), 200 nm (right) (inset, 15 nm).

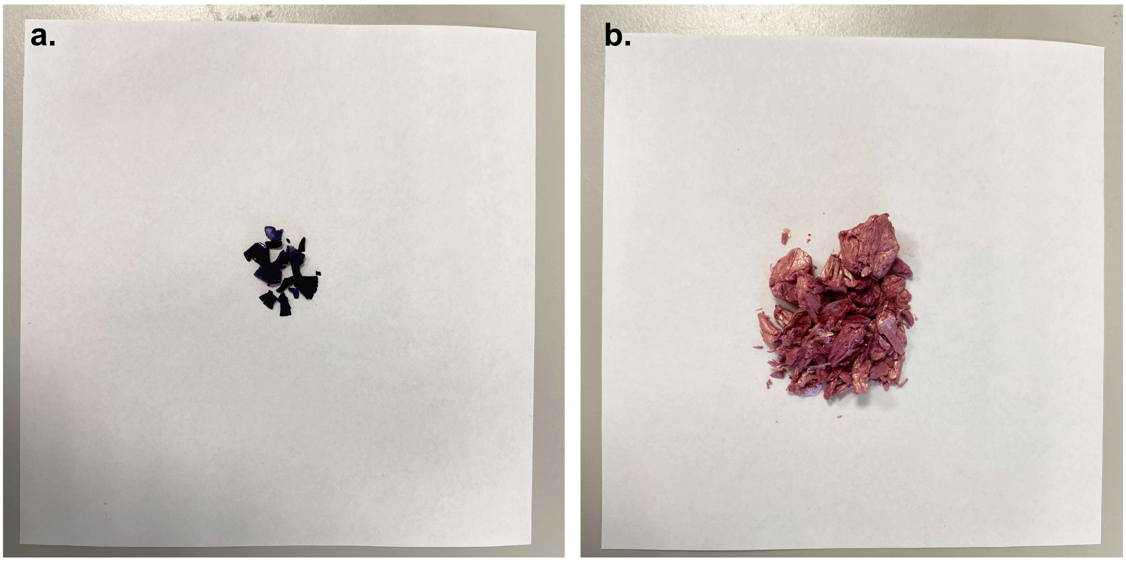

Figure S6. Photographs of **a.** ambient-dried PCN-222@PEG-PO_3 and **b.** lyophilized PCN-222@PEG-PO_3 (both have the same mass, 60 mg).

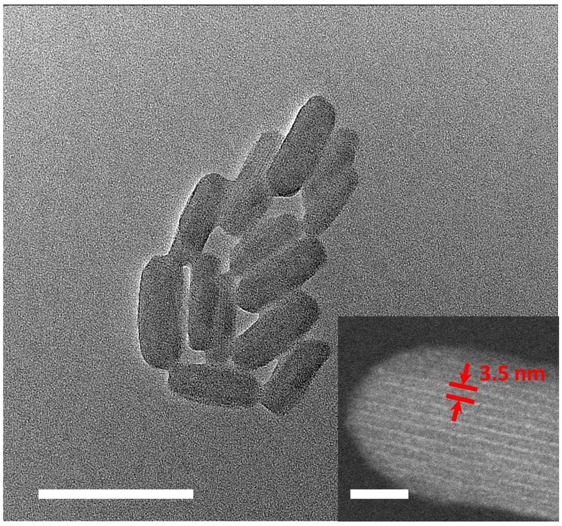

Figure S7. TEM and HAADF-STEM (inset) images of PCN-222@PEG-PO_3. Scale bar, 200 nm (inset, 15 nm).

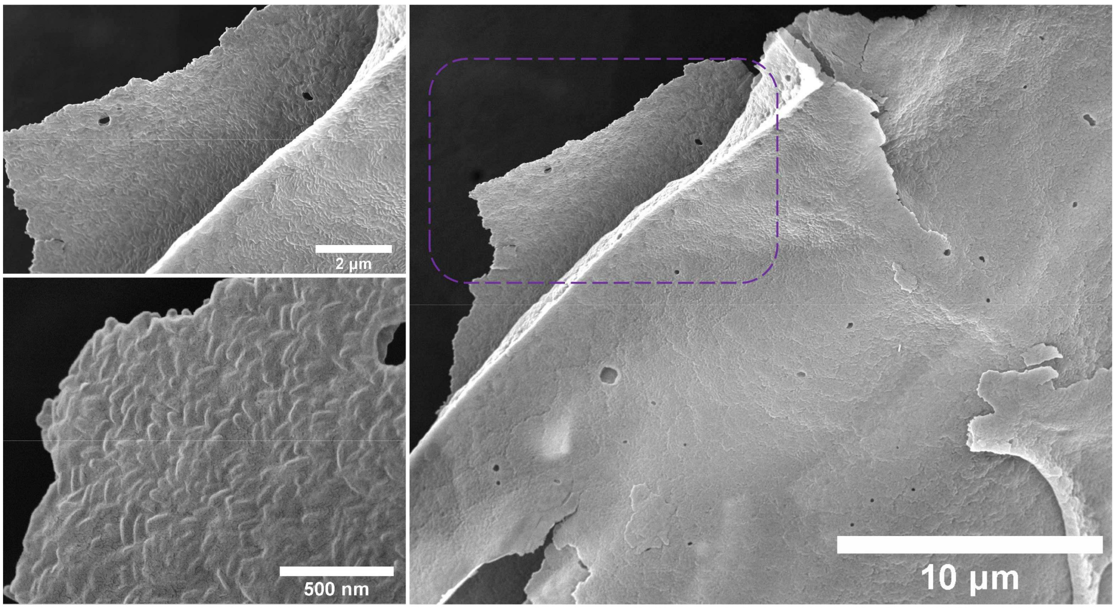

Figure S8. SEM images of lyophilized PCN-222@PEG-PO_3 at different magnifications, clearly suggesting that the PCN-222@PEG-PO_3 are deeply immersed in the mPEG matrix.

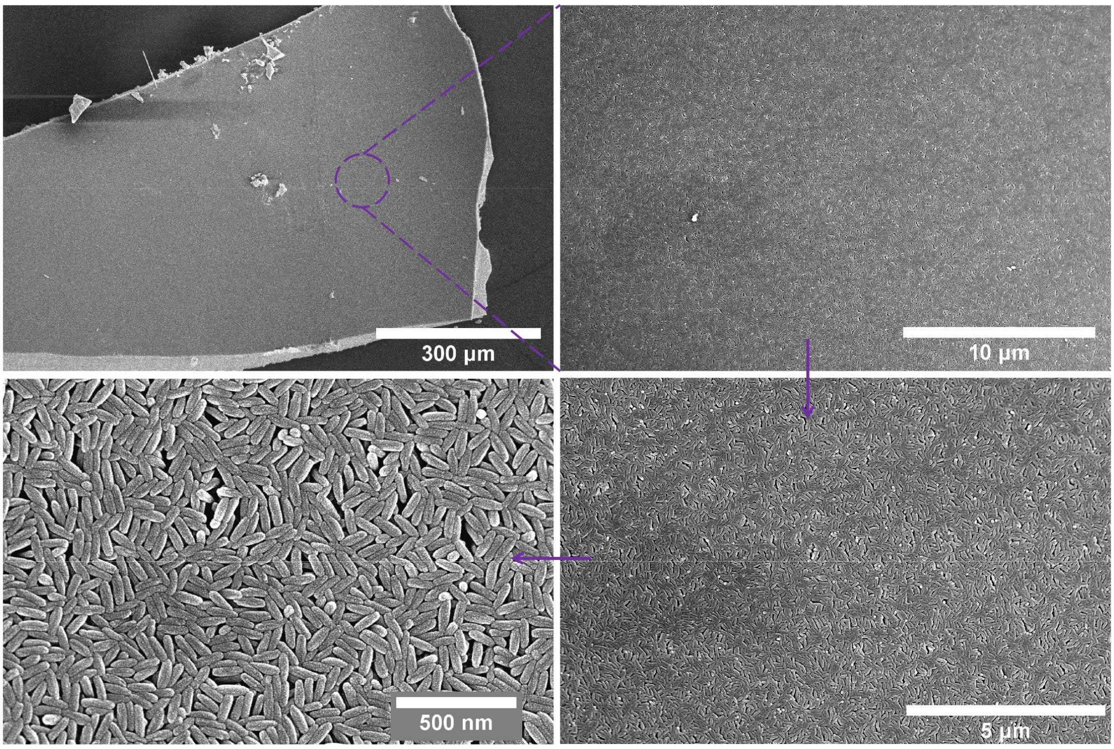

Figure S9. SEM images of ambient-dried PCN-222@PEG-PO_3 with different magnifications, which clearly indicate the close packing and uniform dispersion of PCN-222@PEG-PO_3 and their rod-shaped morphology.

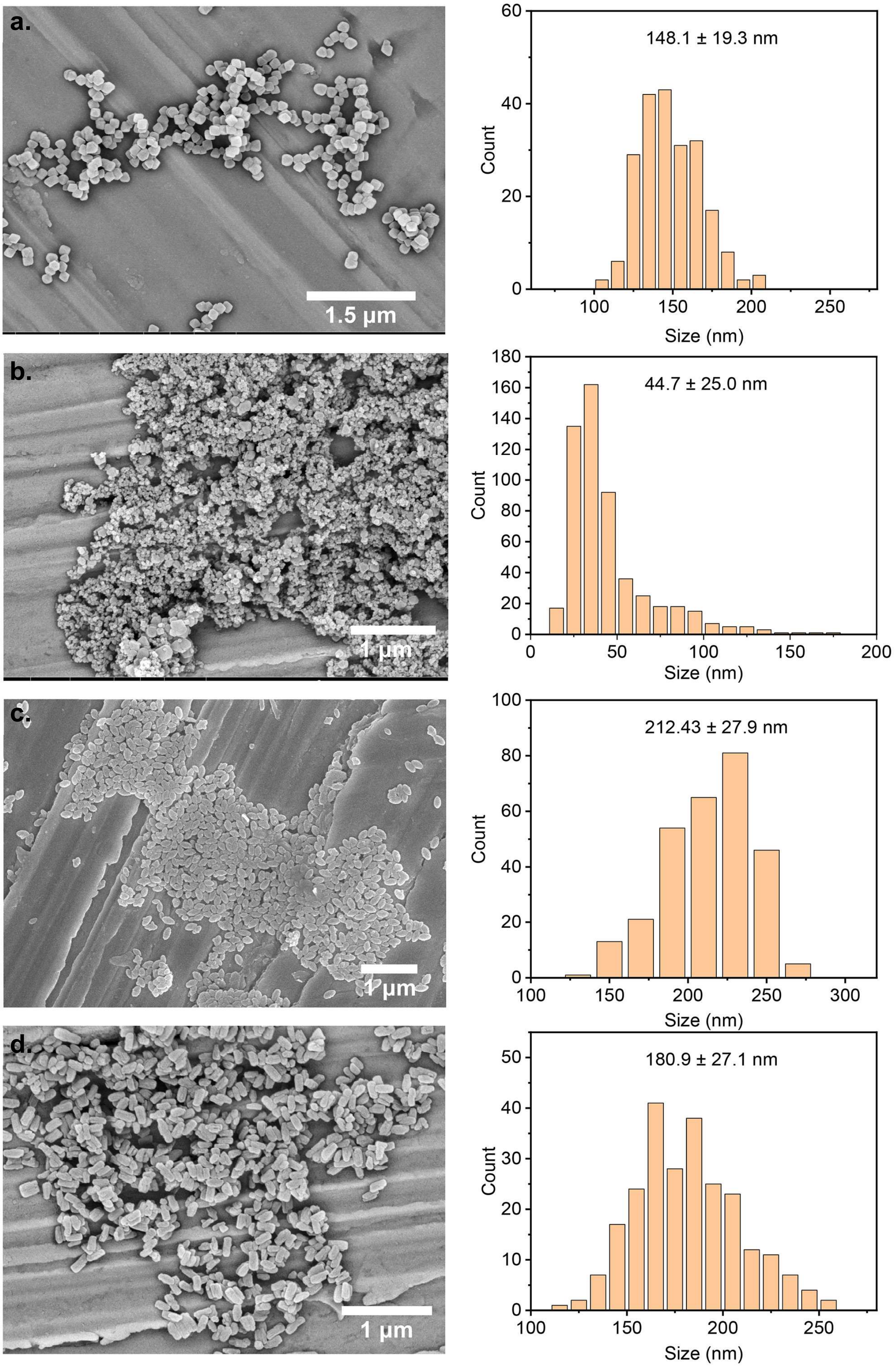

Figure S10. SEM images and particle size analysis of **a.** UiO-66, **b.** MOF-808, **c.** NU-901 and **d.** PCN-128. Average particle sizes were determined by manually counting at least 100 particles.

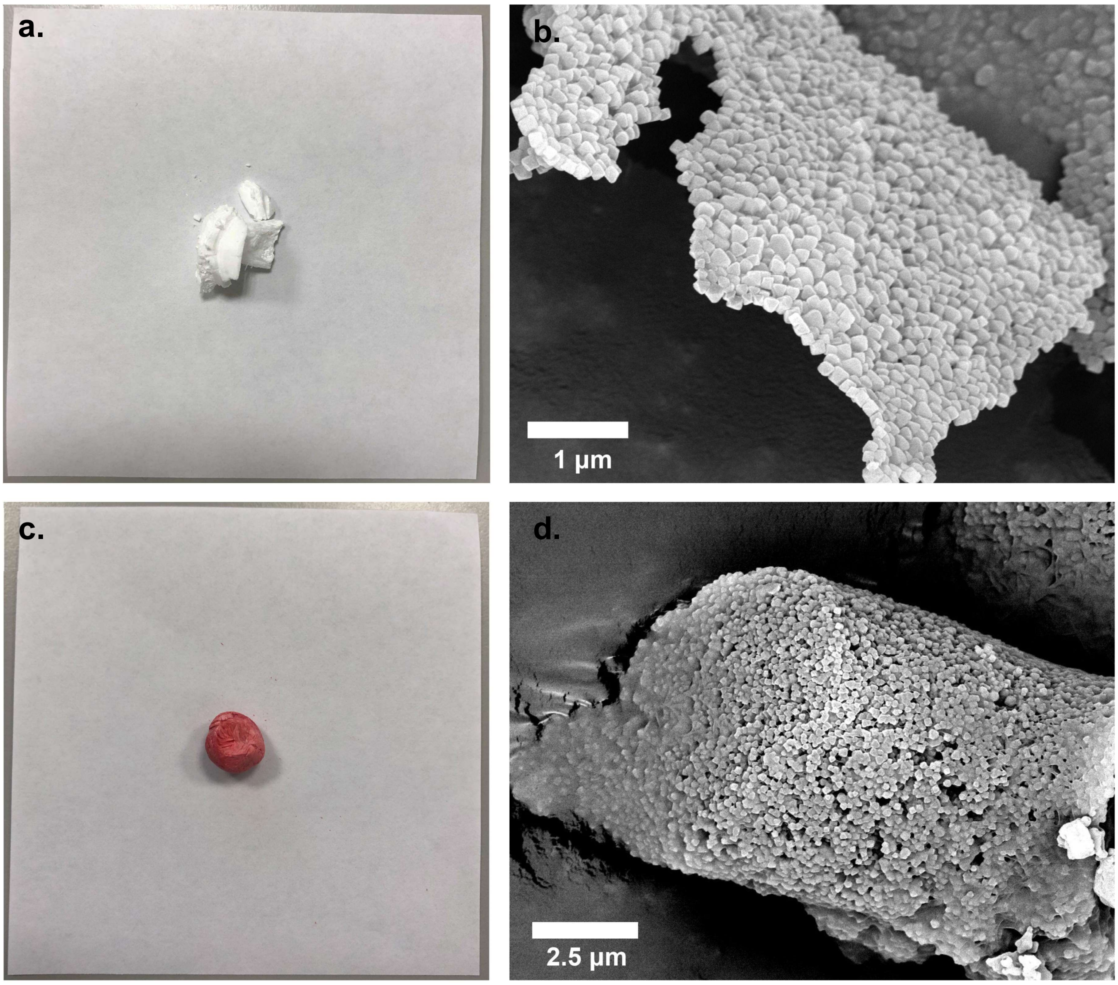

Figure S11. Morphological characterization. **a.** and **c.** Optical images, **b.** and **d.** SEM images of UiO-66@PEG-PO_3 and DOX@66@PEG-PO_3.

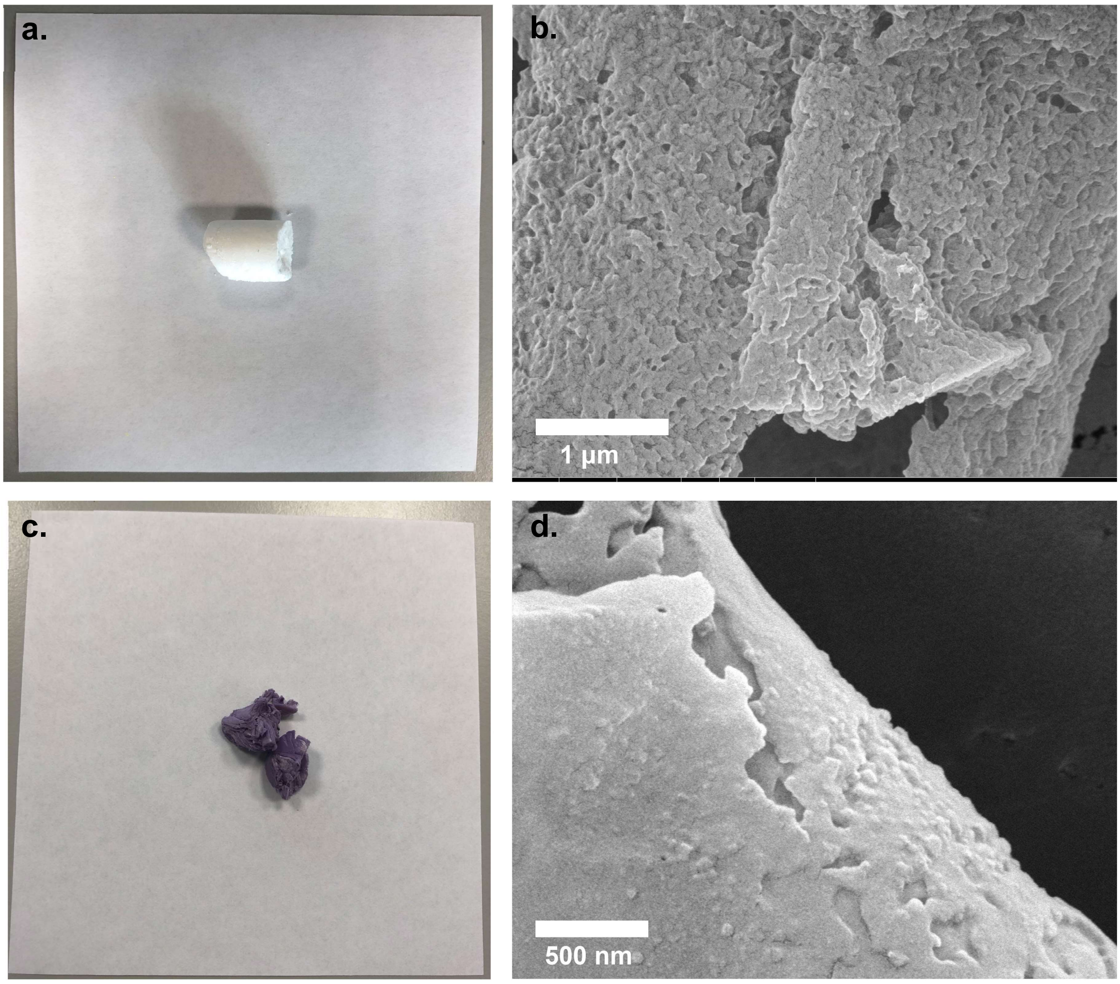

Figure S12. Morphological characterization. **a.** and **c.** Optical images, **b.** and **d.** SEM images of MOF-808@PEG-PO_3 and DOX@808@PEG-PO_3.

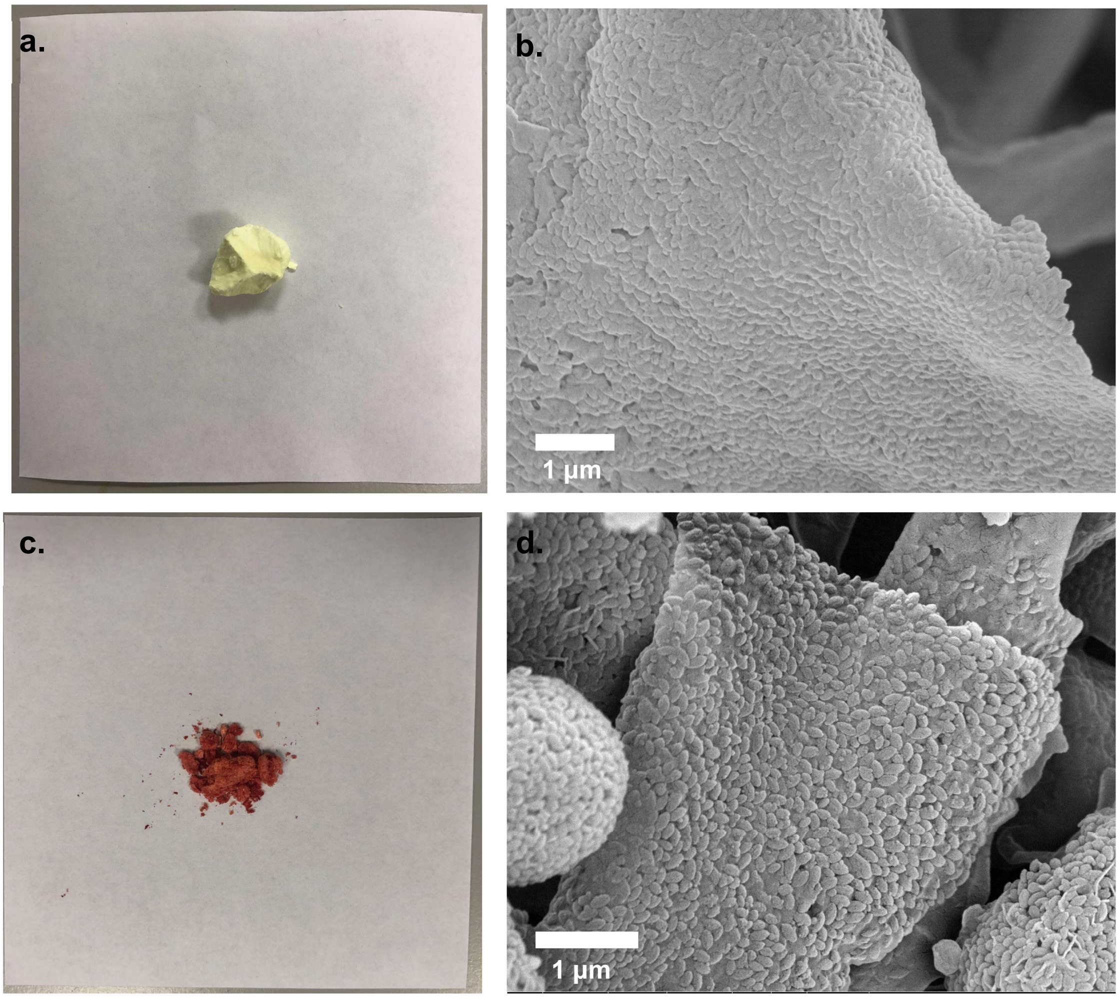

Figure S13. Morphological characterization. **a.** and **c.** Optical images, **b.** and **d.** SEM images of NU-901@PEG-PO_3 and DOX@901@PEG-PO_3.

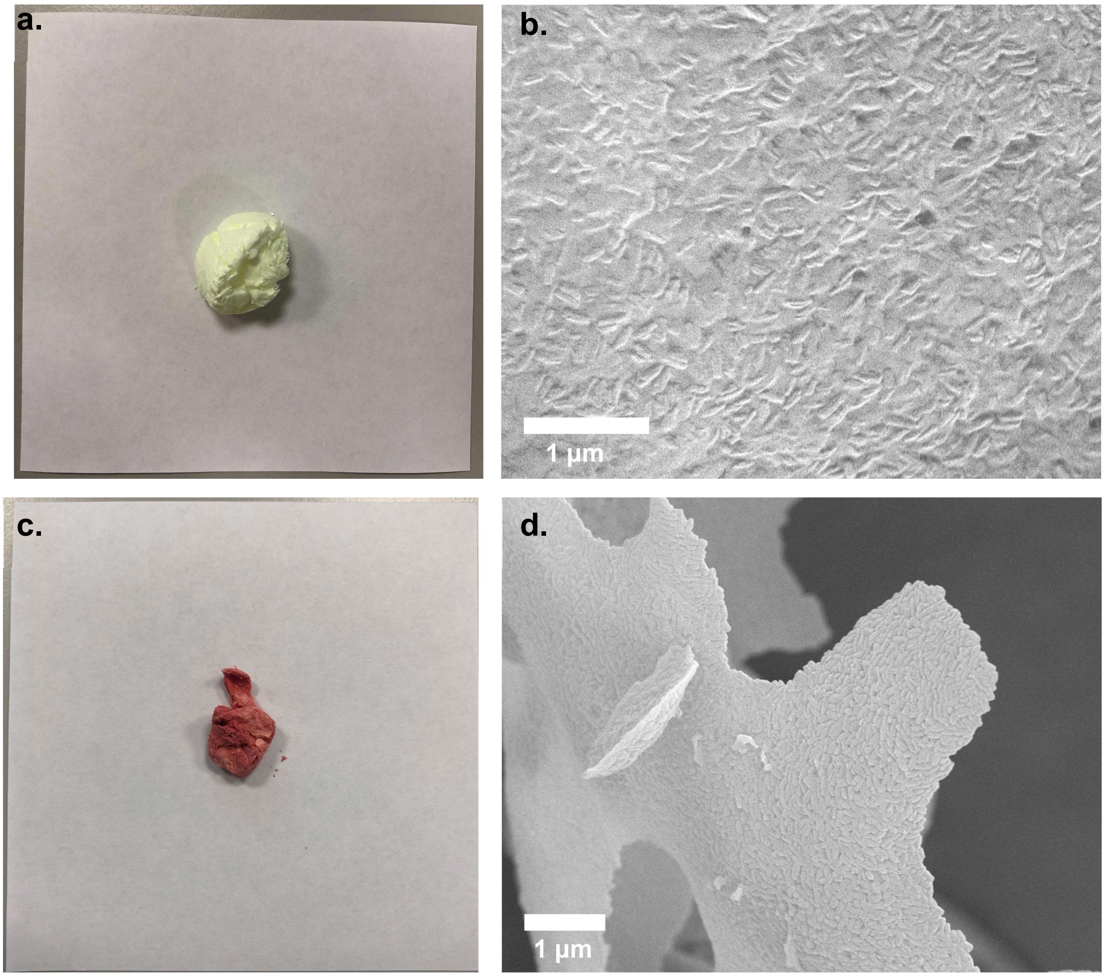

Figure S14. Morphological characterization. **a.** and **c.** Optical images, **b.** and **d.** SEM images of PCN-128@PEG-PO_3 and DOX@128@PEG-PO_3.

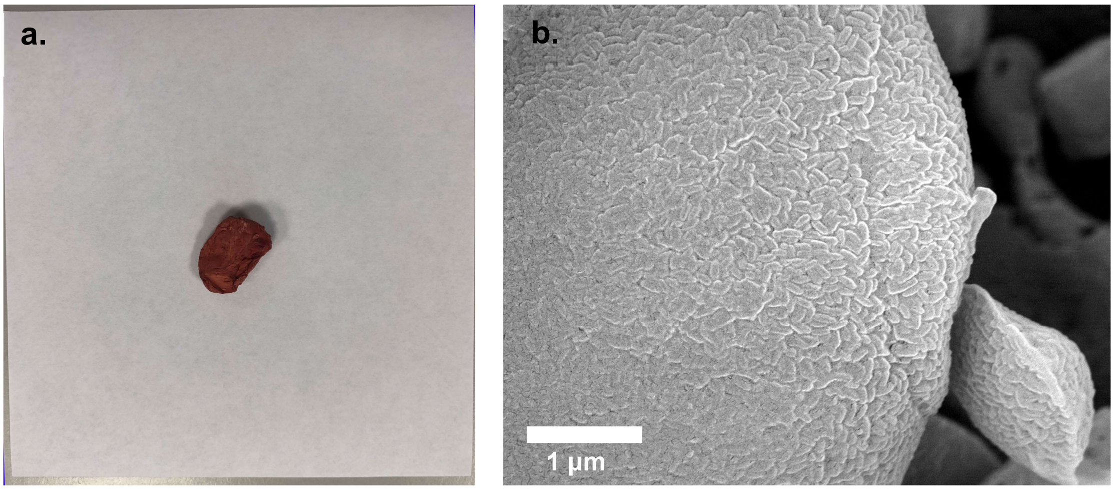

Figure S15. Morphological characterization. **a.** Optical image, **b.** SEM image of DOX@222@PEG-PO_3.

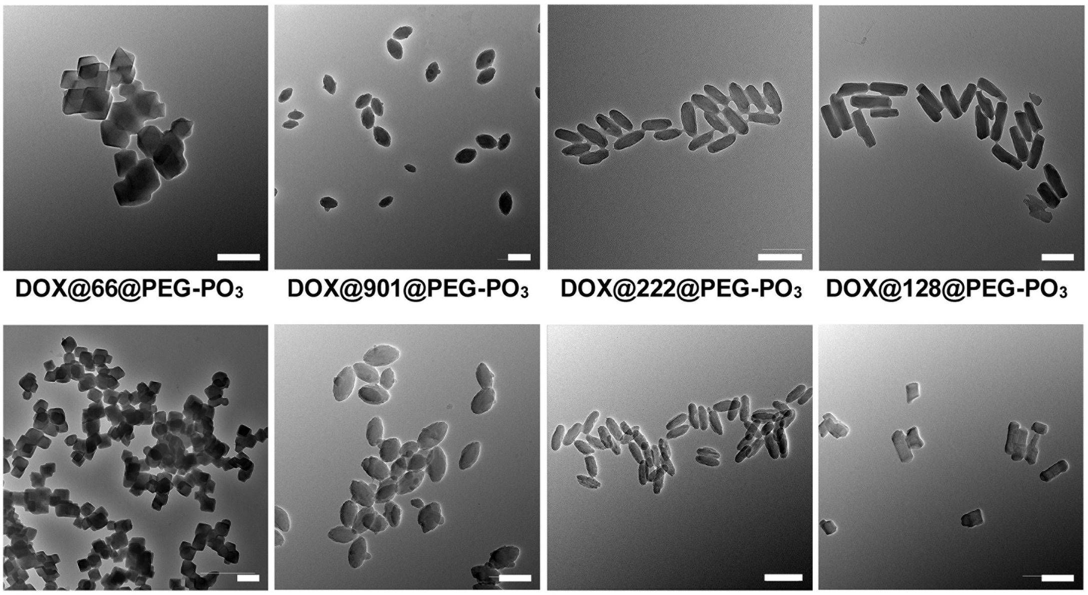

Figure S16. TEM images of DOX-loaded bare and PEGylated MOFs in water (pH =7.4) after 14 days. Scale bar: 200 nm.

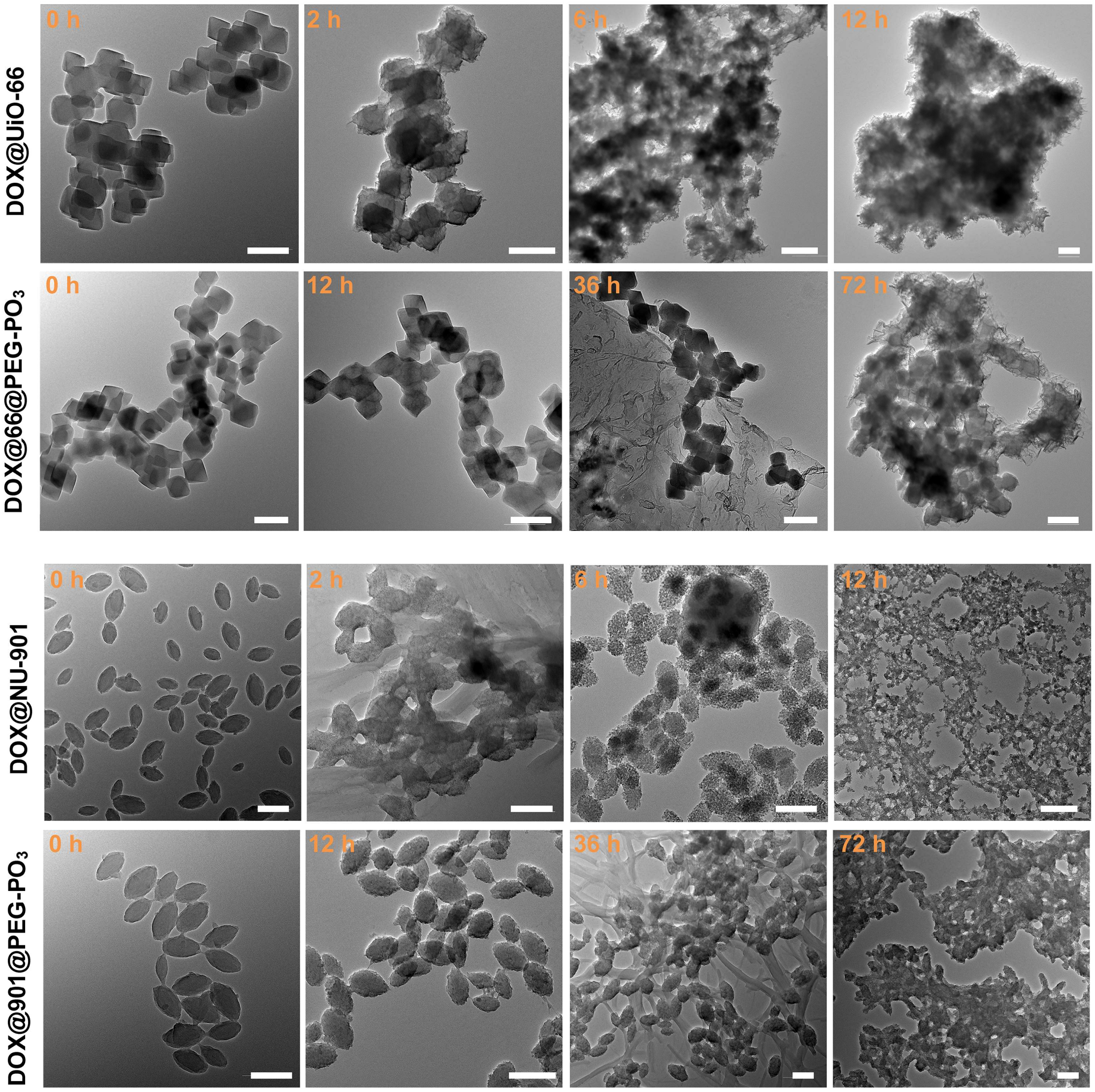

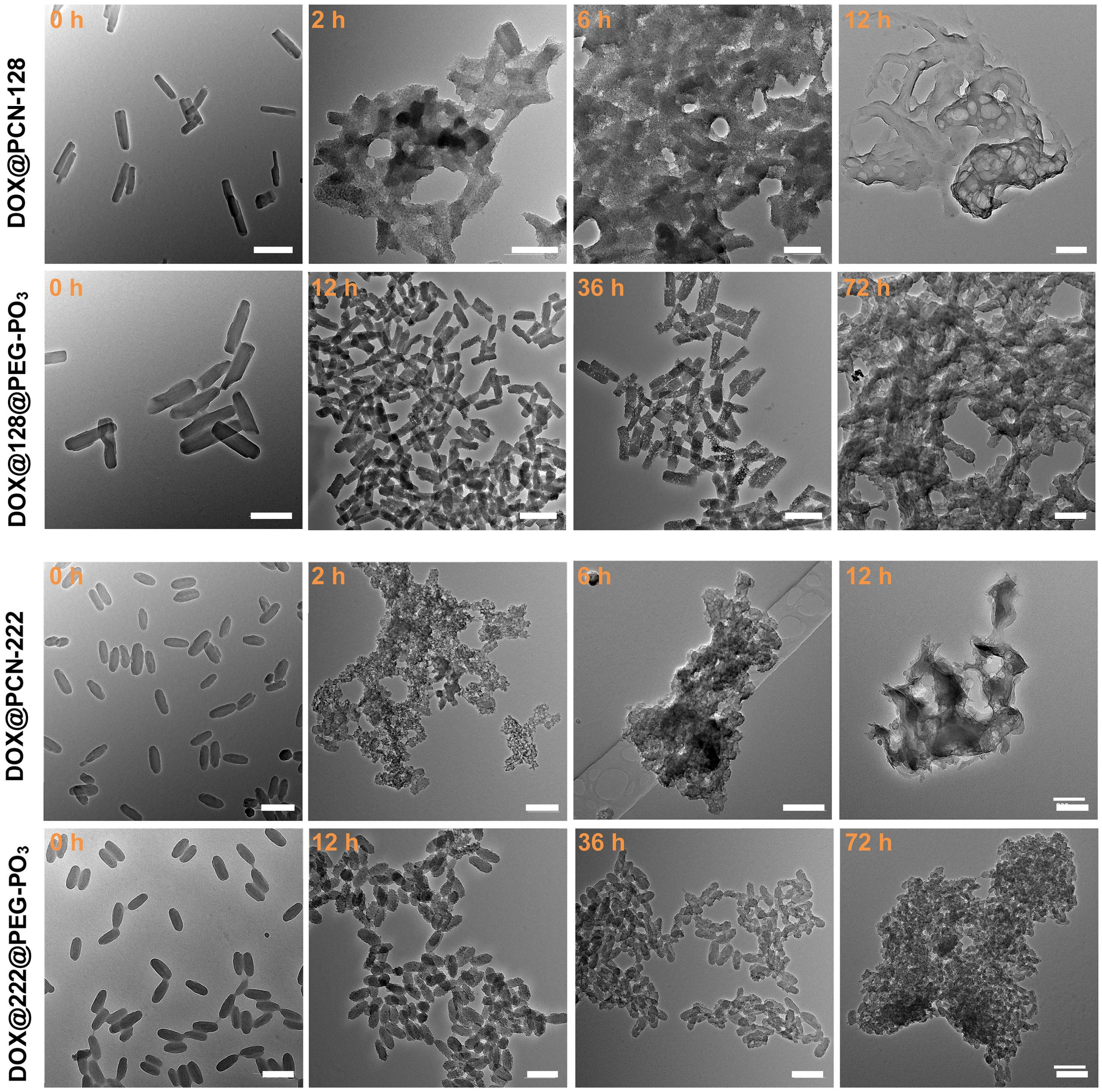

Figure S17. Time-dependent stability of DOX-loaded bare and PEGylated MOFs in PBS (pH =7.4) monitored by TEM. Scale bar: 200 nm.

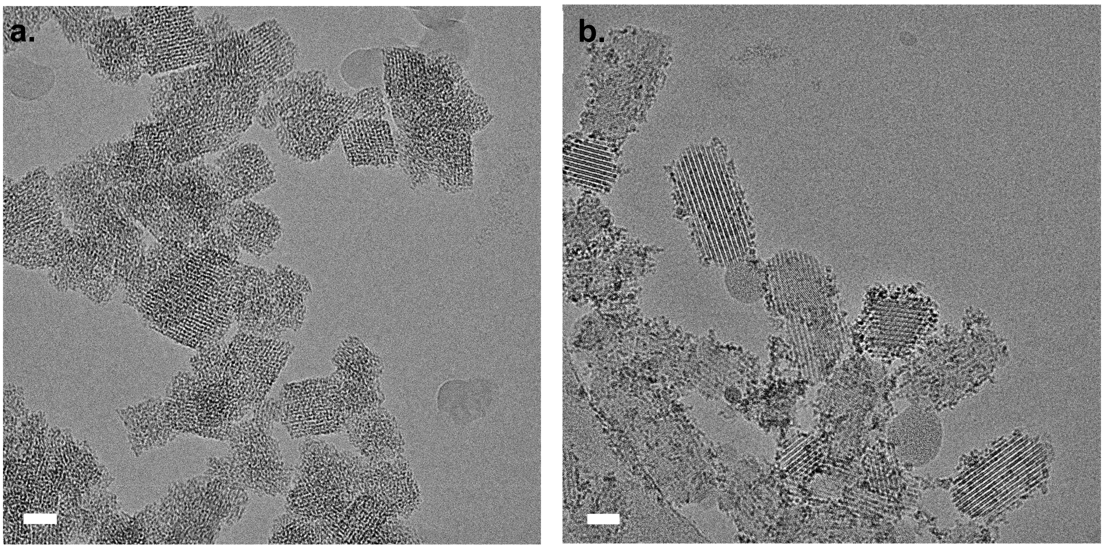

Figure S18. The Cryo-EM images of **a.** UiO-66@PEG-PO_3 and **b.** PCN-222@PEG-PO_3 after treating with PBS (pH = 7.4) for 24 h. Scale bar: 50 nm.

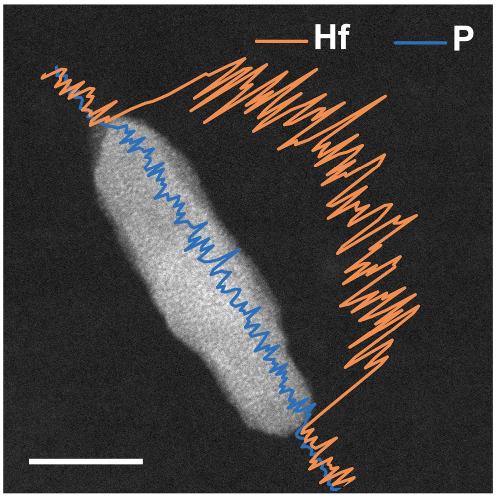

Figure S19. STEM image and EDX spectra of PCN-222@PEG-PO_3 after PEGylation of 16 h. EDX line scans for Hf and P are in brown and blue, respectively. Scale bar, 50 nm.

XPS spectra

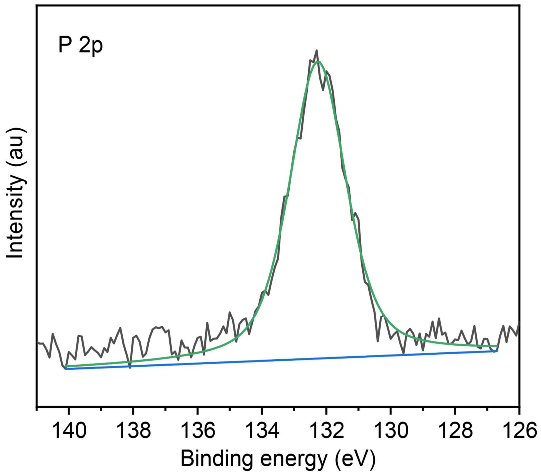

Figure S20. P 2p XPS spectrum of PCN-222@PEG-PO_3.

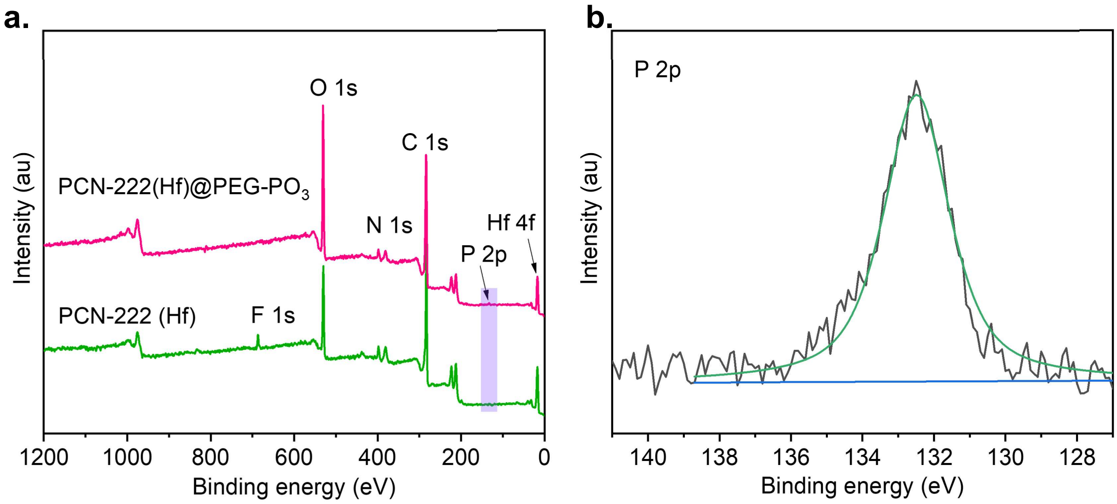

Figure S21. XPS spectra of PCN-222 (Hf) and PCN-222(Hf)@PEG-PO_3. **a.** XPS survey spectrum and **b.** high-resolution of P 2p spectrum of PCN-222(Hf)@PEG-PO_3.

Photographs of the suspension and the related Tyndall effects

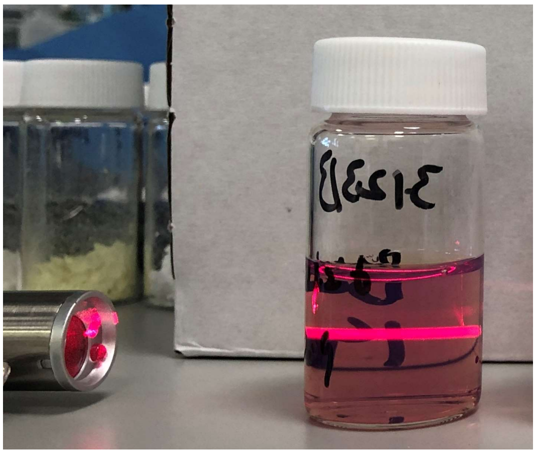

Figure S22. Typical Tyndall effect of PCN-222@PEG-PO_3.

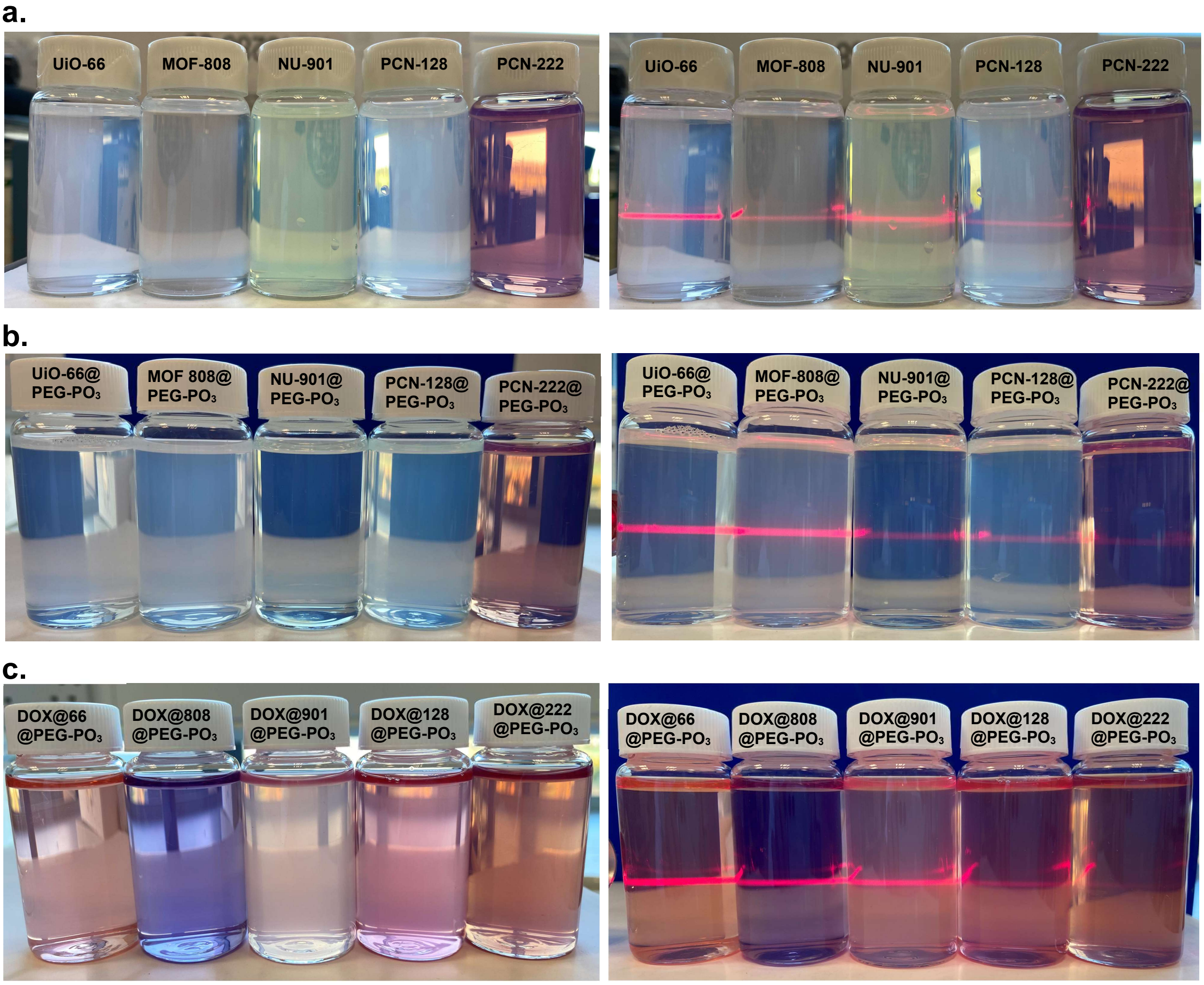

Figure S23. Optical images of the suspension and the related Tyndall effects at nanoMOFs concentration of 0.5 mg/mL. **a.** Bare nanoMOFs, **b,** and **c,** the redispersed MOF@PEG-PO_3 and DOX@ MOF@PEG-PO_3. The purple suspension was obtained for DOX@808@PEG-PO_3, this is due to the complexation between DOX and Zr^{4+} that released from MOF-808.[17] The UV-Vis spectra are shown in **Figure S30**.

PXRD patterns

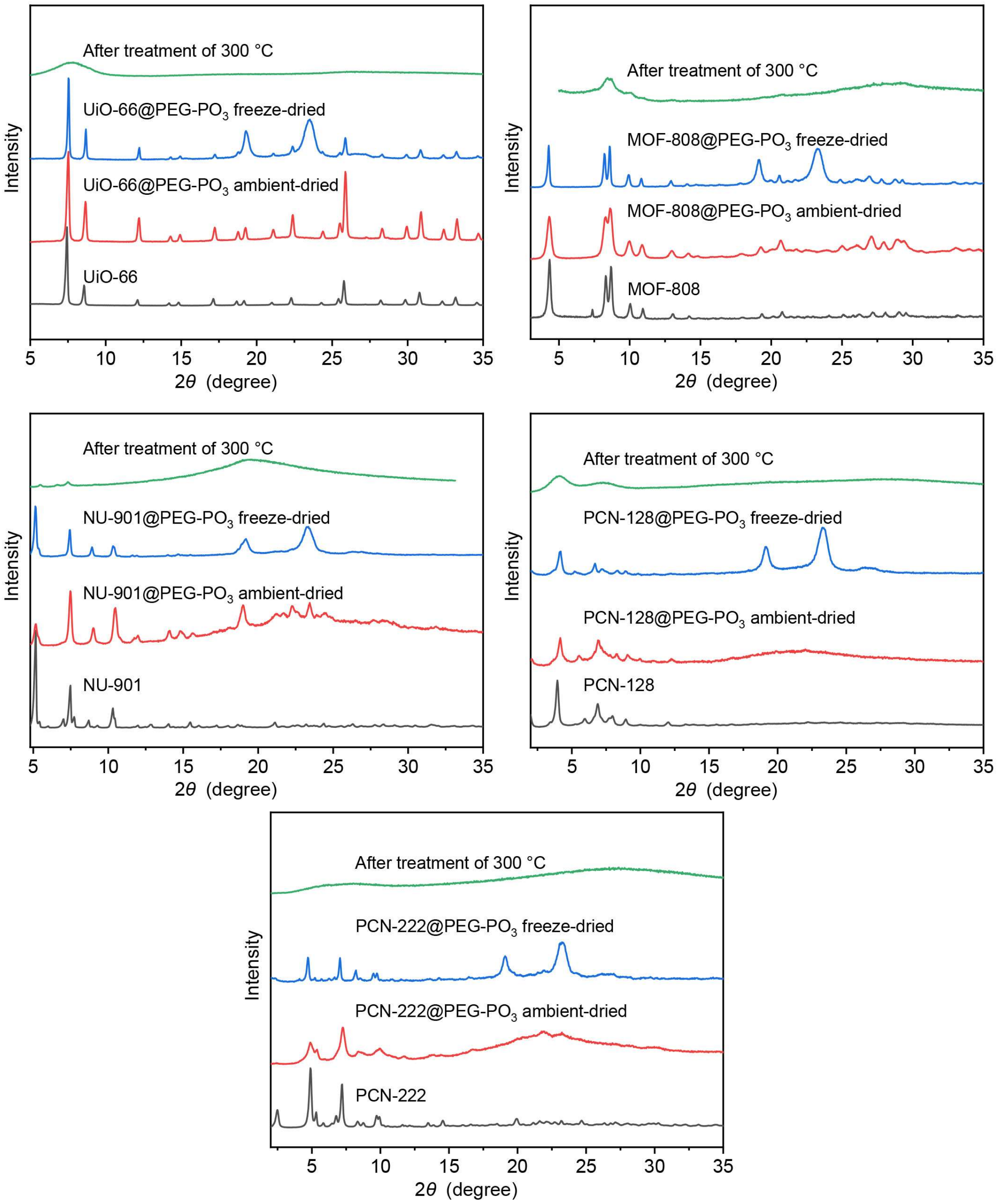

Figure S24. PXRD patterns of bare nanoMOFs, ambient-dried and lyophilized MOF@PEG-PO_3, and MOF@PEG-PO_3 after heating under 300 °C for 6 h.

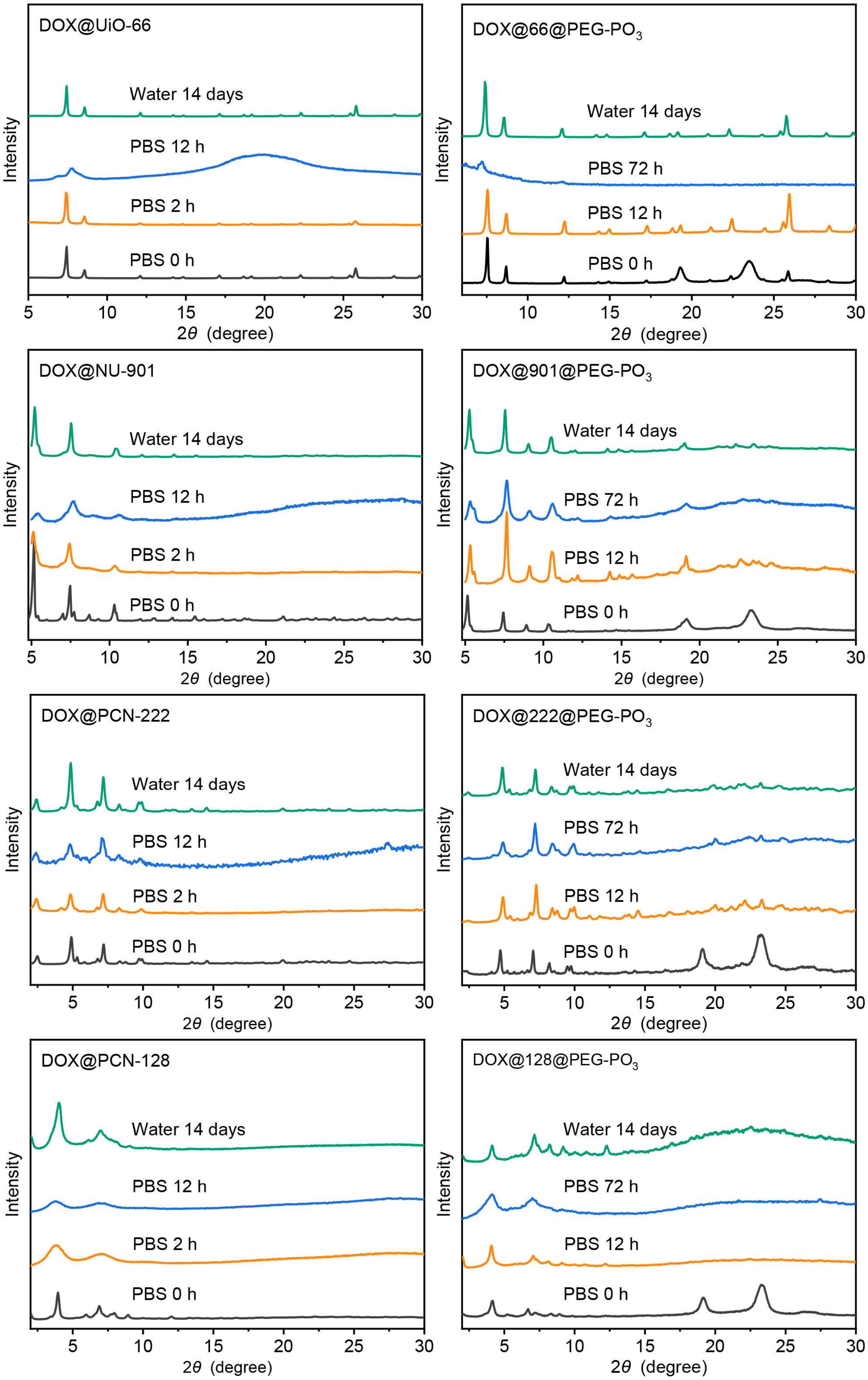

Figure S25. Time-dependent stability in PBS (pH = 7.4) or water monitored by PXRD.

TGA curves

.

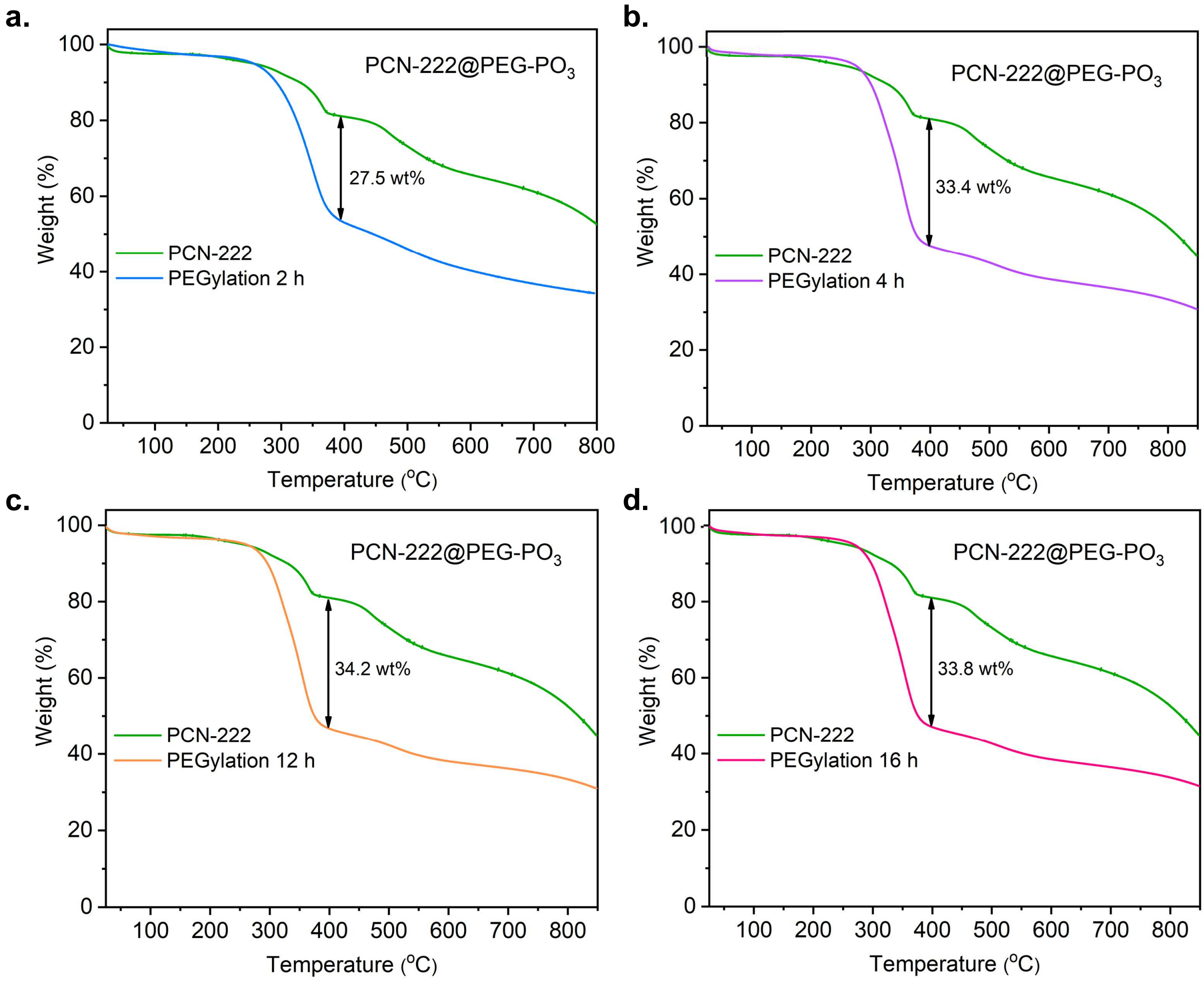

Figure S26. TGA profiles of PCN-222@PEG-PO_3: **a.** PEGylation 2 h, **b.** PEGylation 4 h, **c.** PEGylation 12 h and **d.** PEGylation 16 h. The encapsulated amount of mPEG-PO_3 was calculated based on the difference between the TGA curve of the first plateau (376-452 °C).[18-19]

.

Additional figure

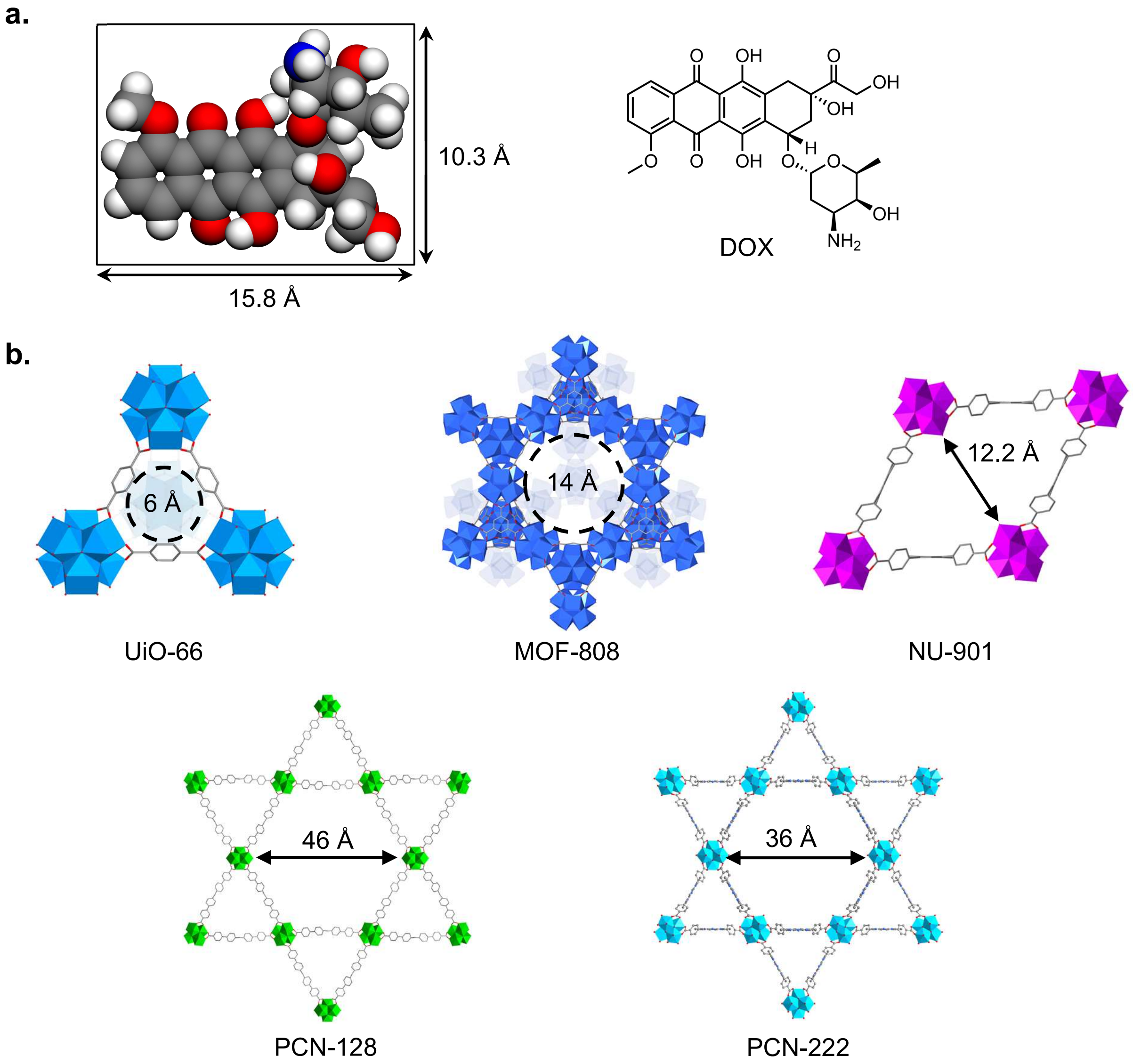

Figure S27. **a.** Calculated molecular model and molecular structures of DOX. (The space-filling models were calculated by Materials Studio). **b.** Illustration of the pore size of UiO-66, MOF-808, NU-901, PCN-128 and PCN-222.

FT-IR spectra

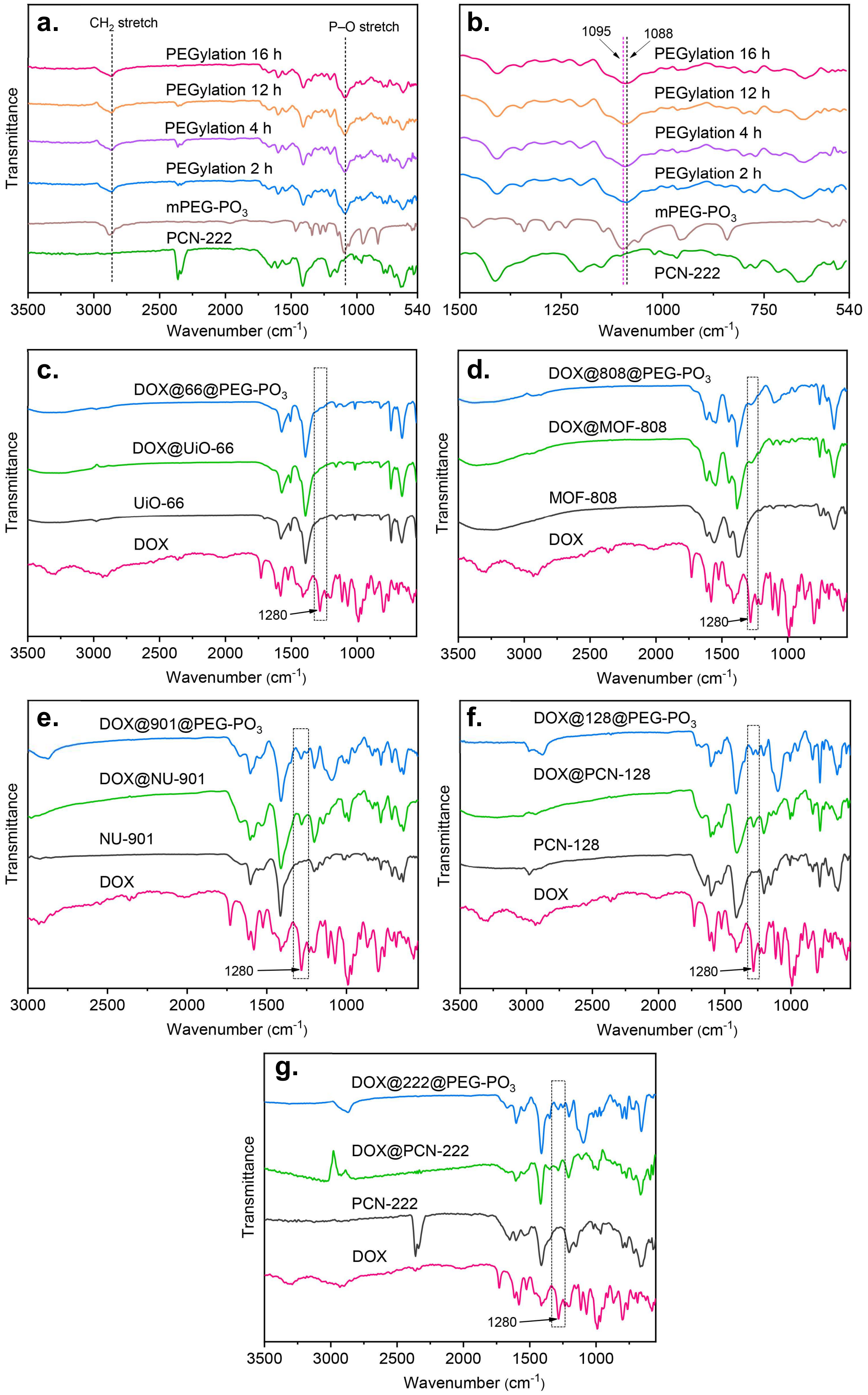

Figure S28. **a.** Full FT-IR spectra. **b.** The enlarged spectra of PEGylated PCN-222 at different time points. The appearance of two new bands at 2866 and 1090 cm^{-1} were attributed to the stretching vibration of C−H and P−O from mPEG-PO$_3$.[20] (**c - g**) FT-IR spectra of DOX, bare nanoMOFs, DOX@MOF, DOX@MOF@PEG-PO$_3$: **c.** UiO-66, **d.** MOF-808, **e.** NU-901, **f.** PCN-128 and **g.** PCN-222.

UV-Vis spectra

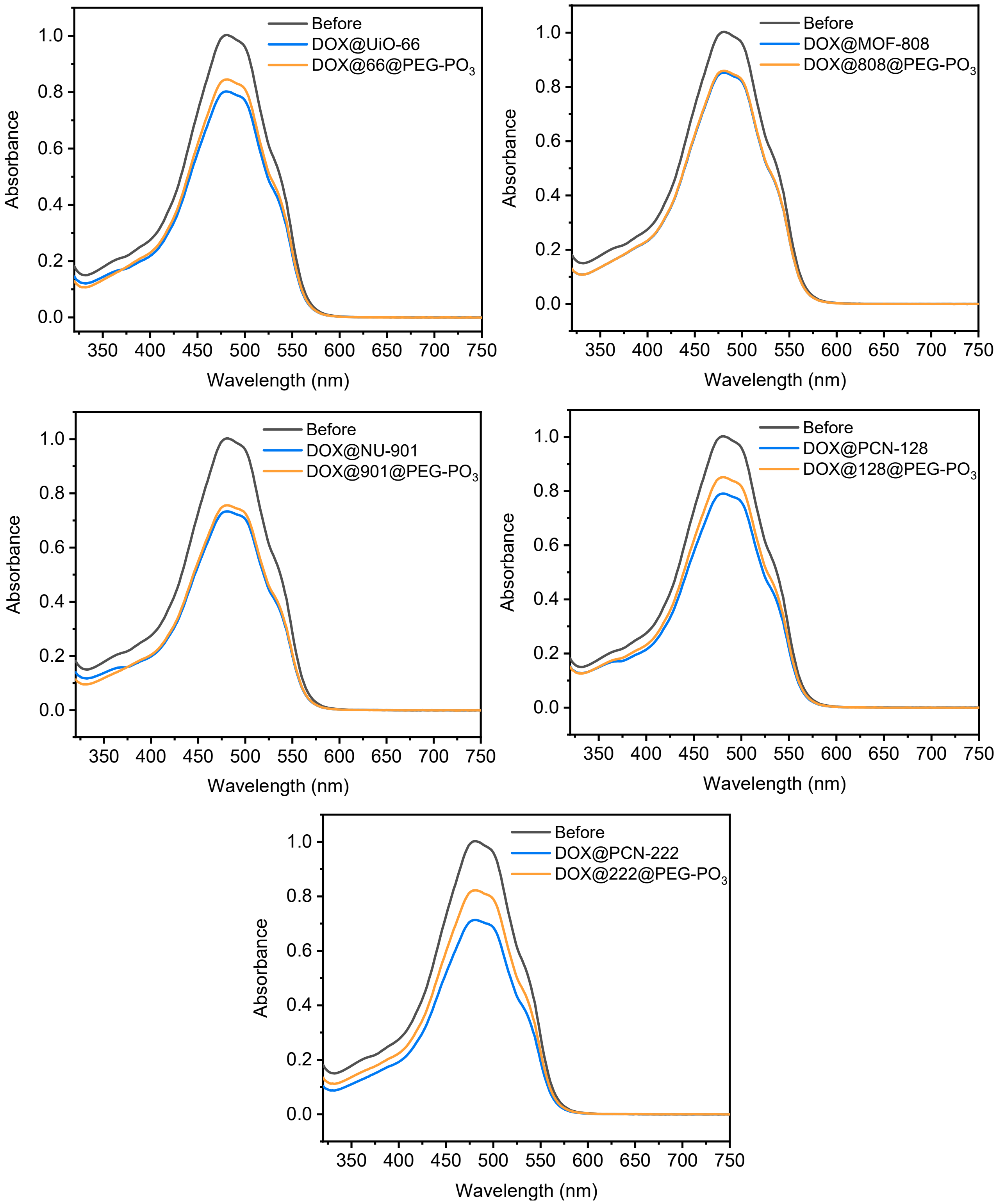

Figure S29. UV-Vis spectra of the pristine DOX solution, after drug loading and PEGylation.

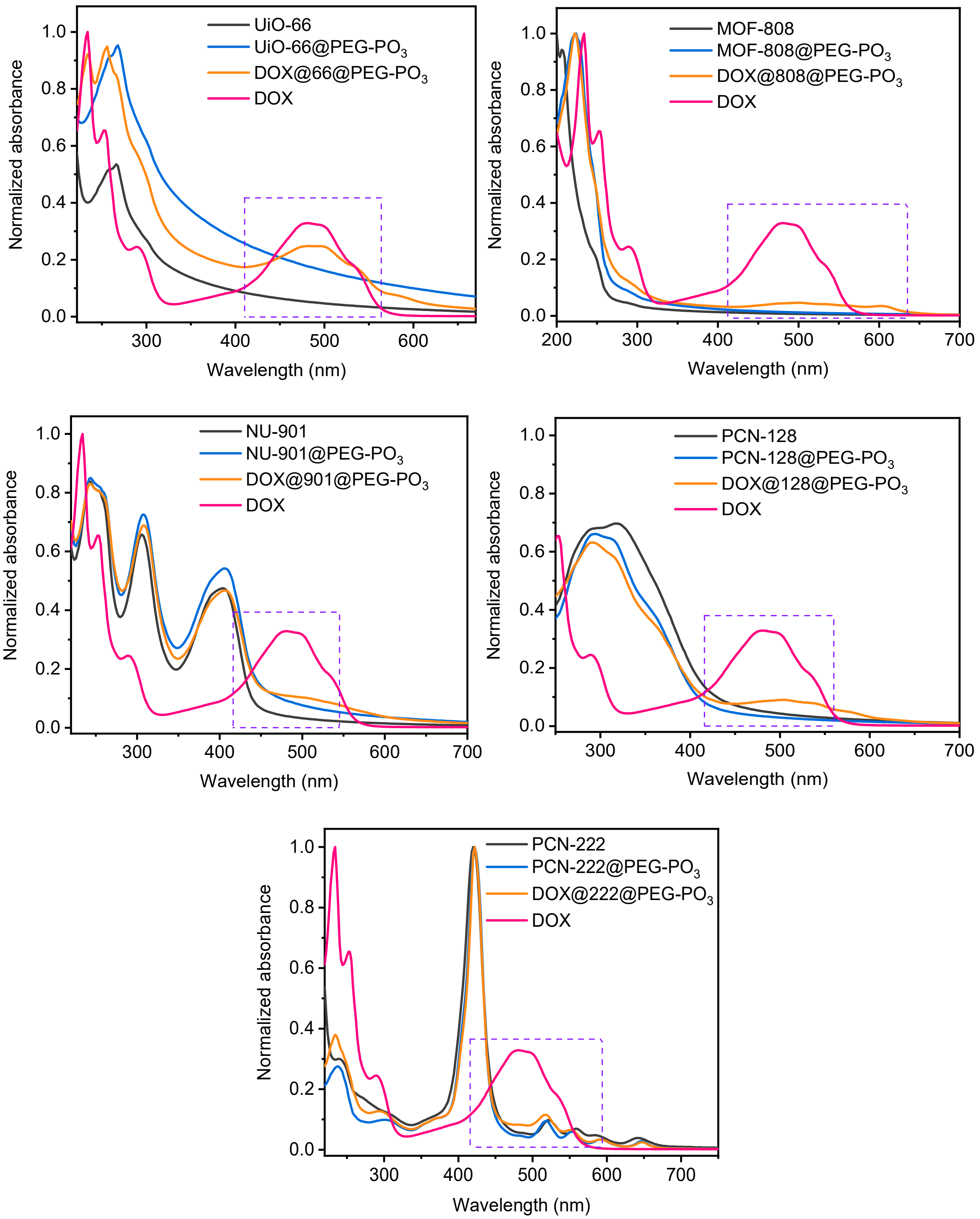

Figure S30. UV-Vis spectra of DOX, bare nanoMOFs, MOF@PEG-PO_3 and DOX@MOF@PEG-PO_3.The purple boxes show the presence of DOX in PEGylated MOFs. It is worth recalling that the absorption spectrum of DOX displays bands at 288 nm and 480−500 nm.[21] We note that there is either a sharp or broad peak in related positions of DOX@MOF@PEG-PO_3. Briefly, peaks ranged from 400 to 600 nm were observed for DOX@66@PEG-PO_3, DOX@901@PEG-PO_3 and DOX@128@PEG-PO_3. In the case of DOX@808@PEG-PO_3, a broad 450 nm to 650 nm was observed. The slight shift of its absorbance of DOX-loaded is due to the complexation between DOX and Zr^{4+} that released from MOF-808.[17] At the same time, the colour of its

suspension has changed into purple (**Figure S23c**). As for the spectrum of DOX@222@PEG-PO_3, the absorption of DOX overlaps with that of PCN-222; that is the reason why the UV absorption peak of DOX in PEGylated PCN-222 is not obvious. In addition, a similar result has also been found in a previously reported paper.[22]

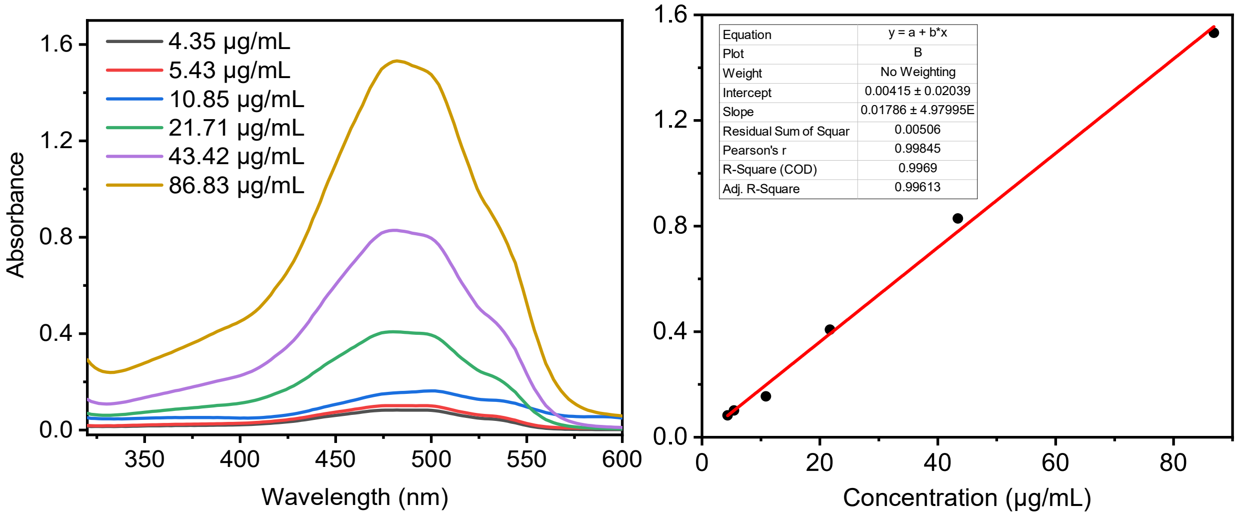

Figure S31. Calibration curve of DOX in H_2O (maximum absorbance at 486 nm).

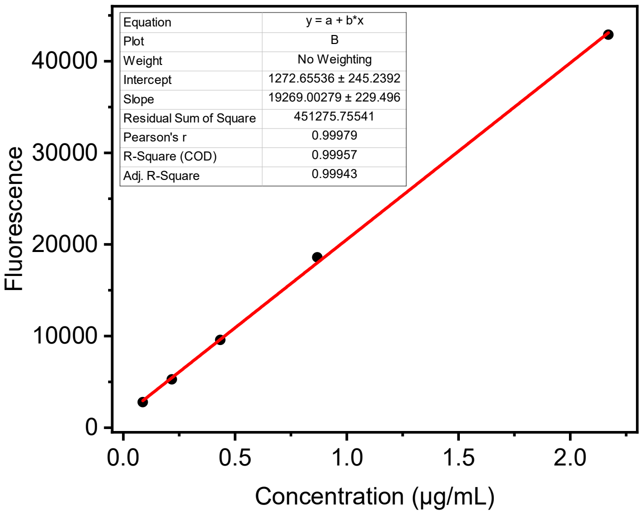

Figure S32. Calibration curve of DOX in PBS (pH = 7.4) (emission maximum at 595 nm when excited at 486 nm).

N_2 sorption isotherms

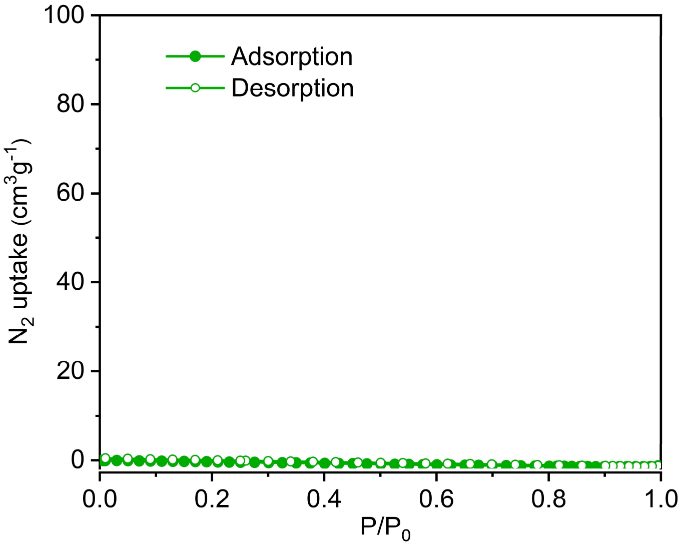

Figure S33. N_2 adsorption isotherms of pure mPEG-PO_3.

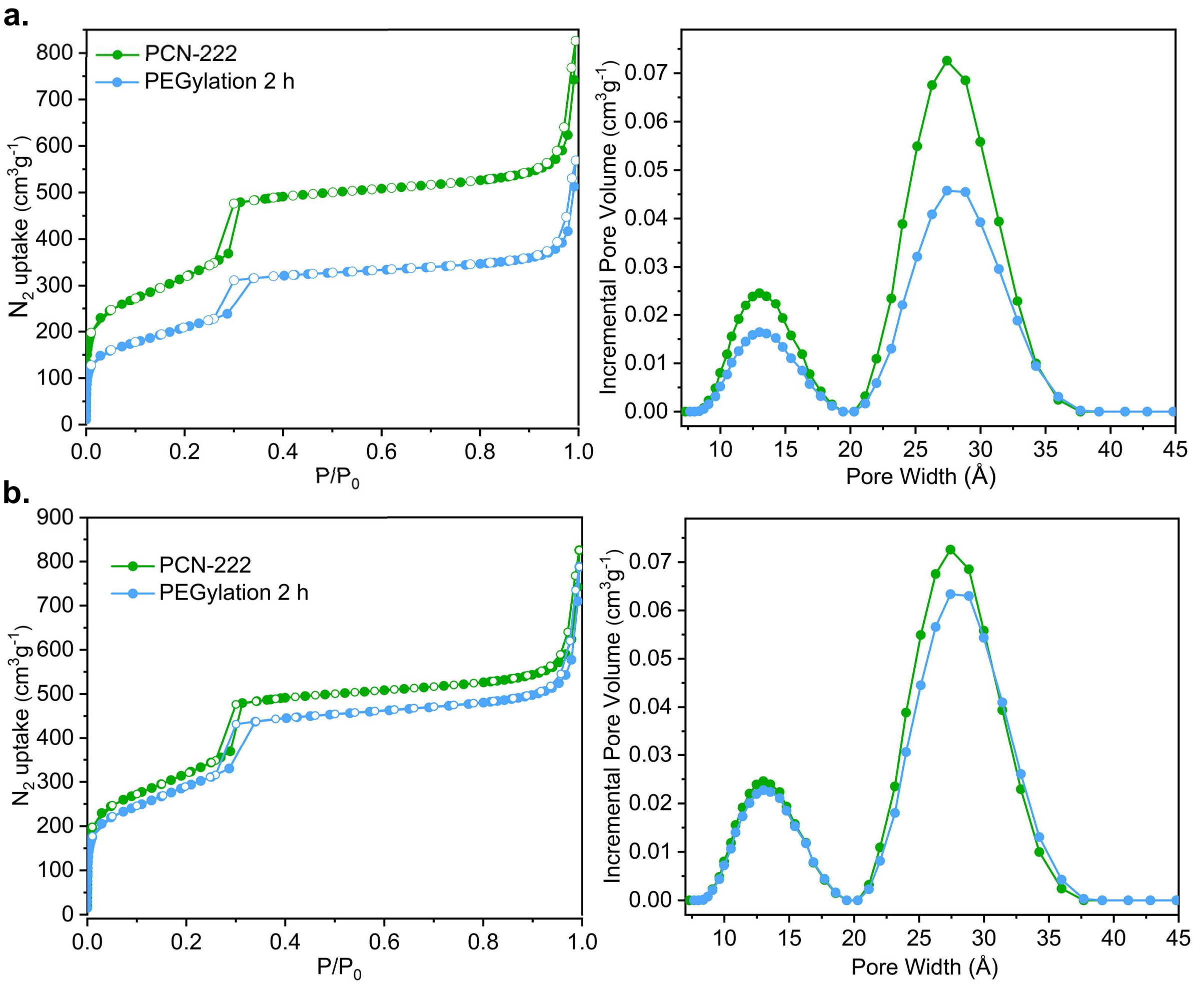

Figure S34. N_2 adsorption isotherms and PSD of PCN-222 and PEGylation 2 h. **a.** before and **b.** after subtracting the amount of mPEG-PO_3 incorporated.

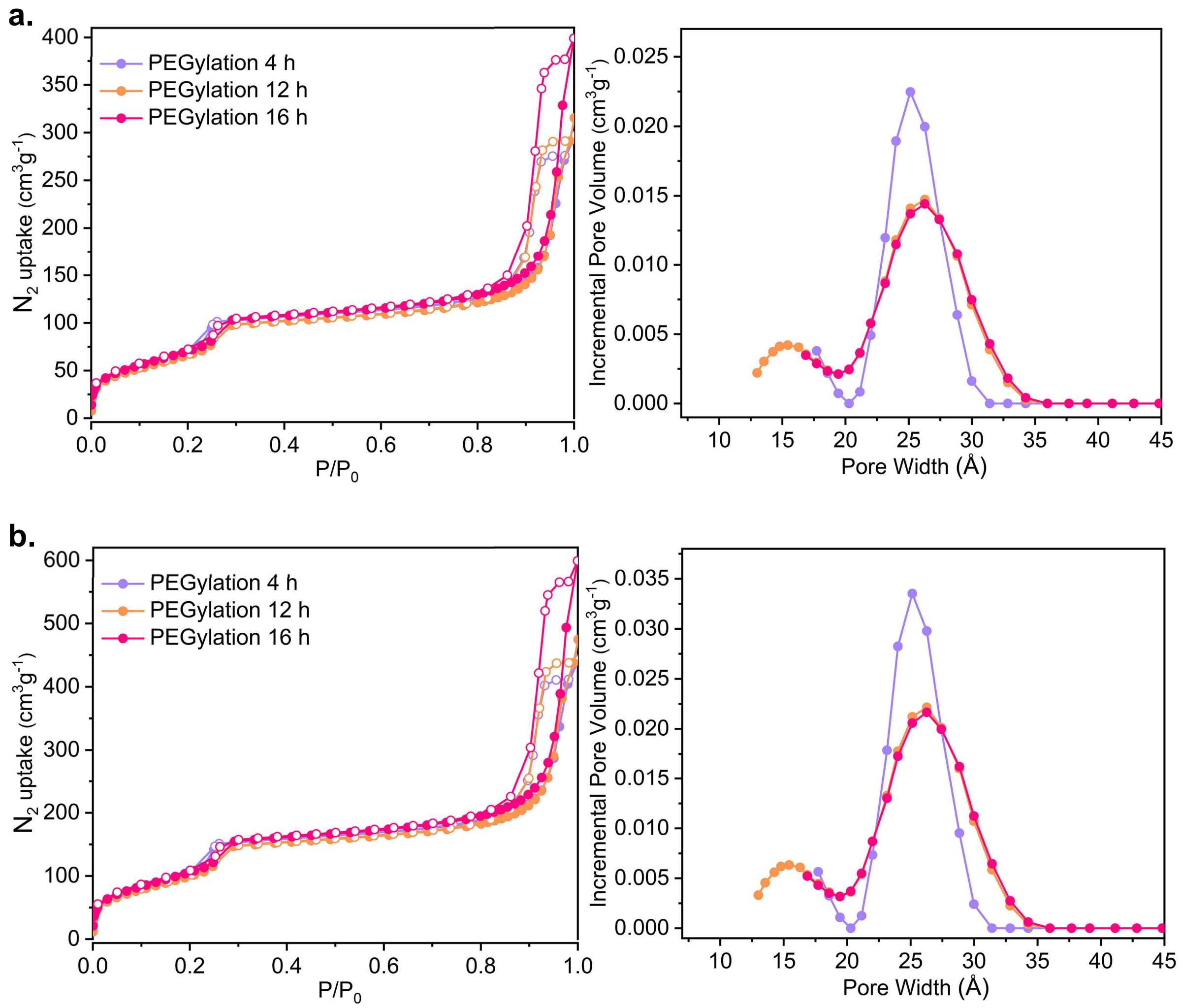

Figure S35. N_2 adsorption isotherms and PSD of PEGylation 4, 12 and 16 h. **a.** before and **b.** after subtracting the amount of mPEG-PO_3 included.

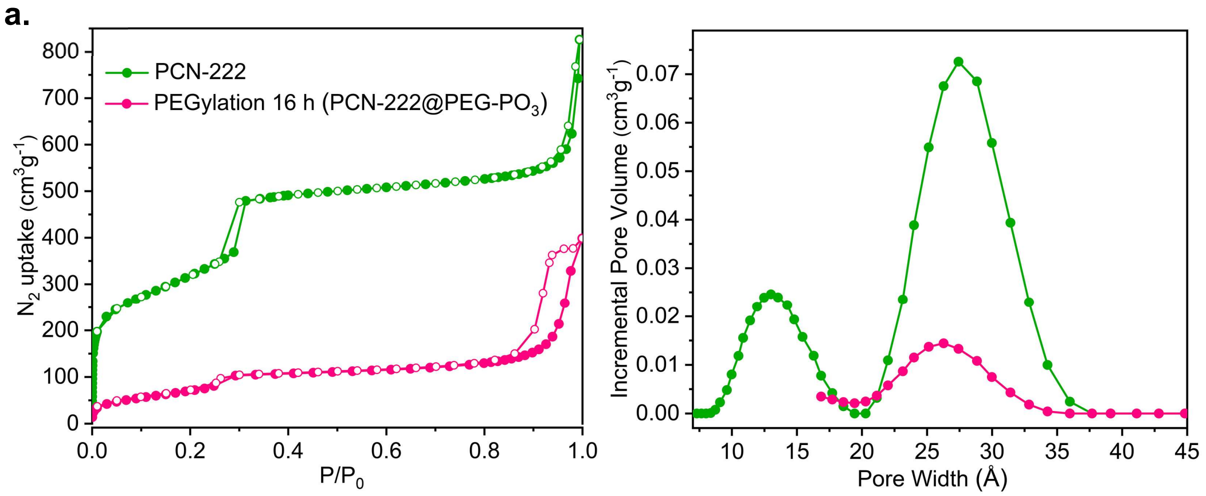

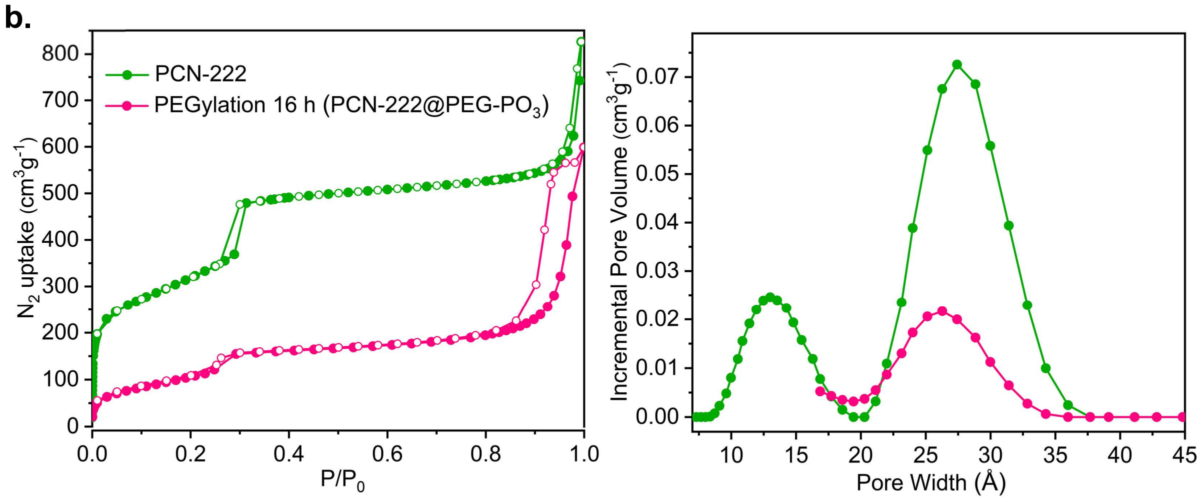

Figure S36. N_2 adsorption isotherms and PSD of PCN-222 and PEGylation 16 h. **a.** before and **b.** after subtracting the amount of mPEG-PO_3 included.

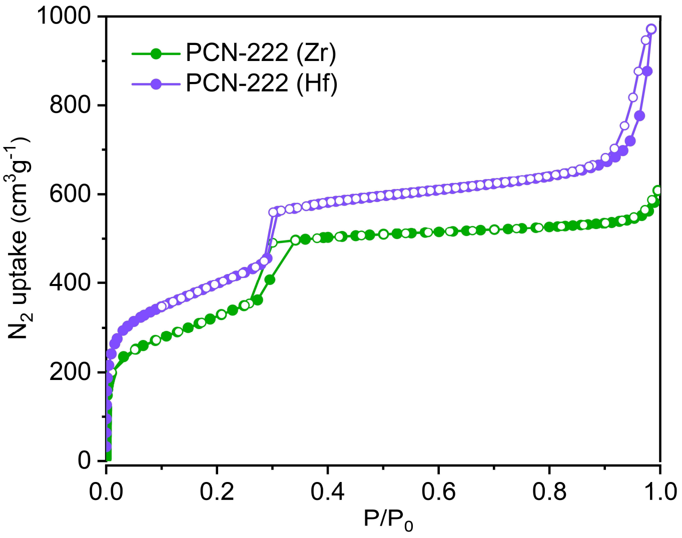

Figure S37. N_2 adsorption isotherms of PCN-222 (Zr and Hf).

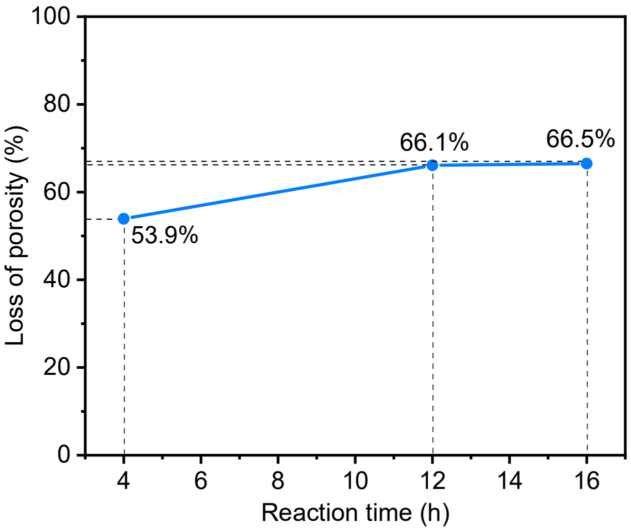

Figure S38. Correlation between loss of porosity and reaction time.

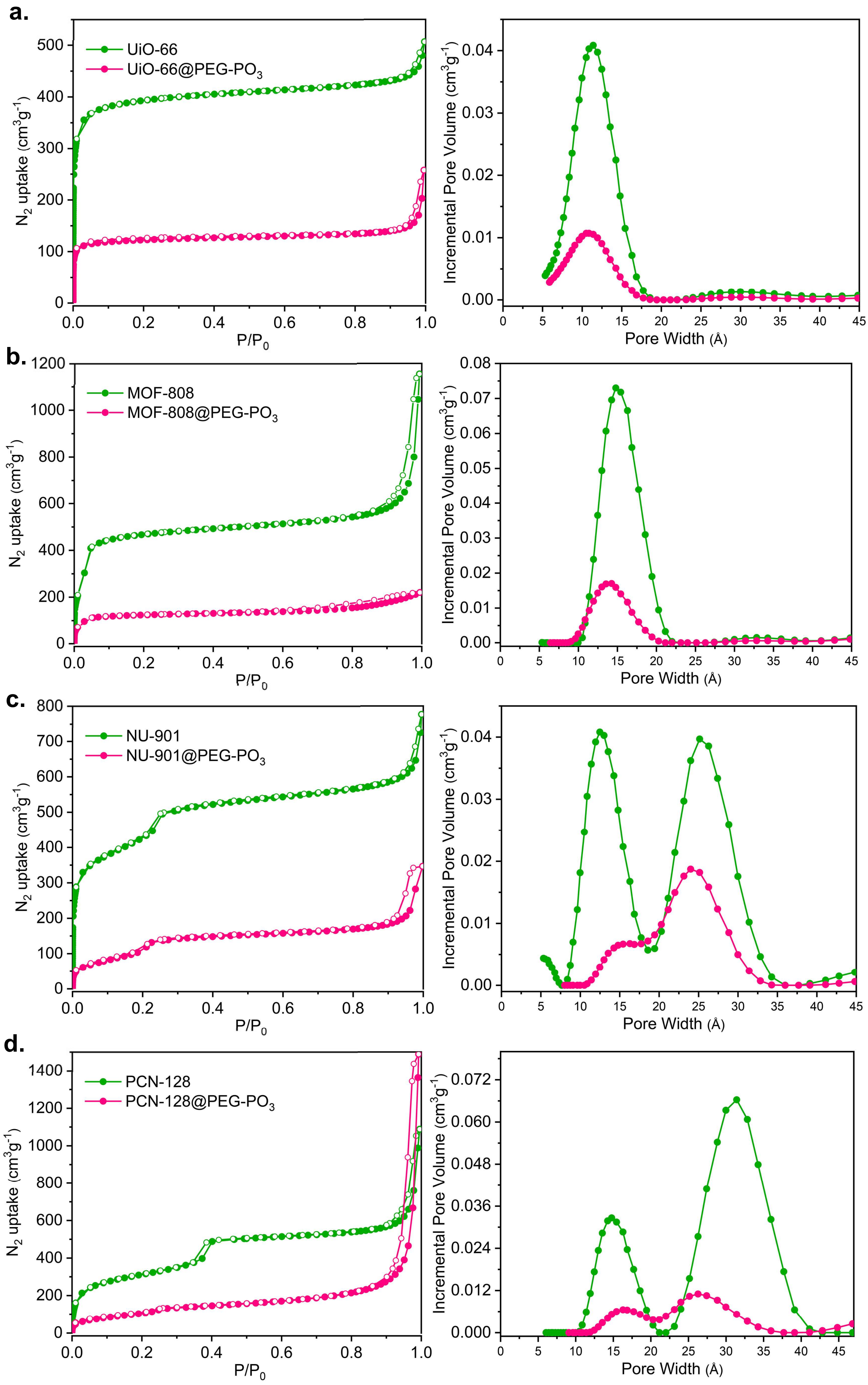

Figure S39. N_2 adsorption isotherms and PSD of MOF@PEG-PO_3. **a.** UiO-66, **b.** MOF-808, **c.** NU-901 and **d.** PCN-128.

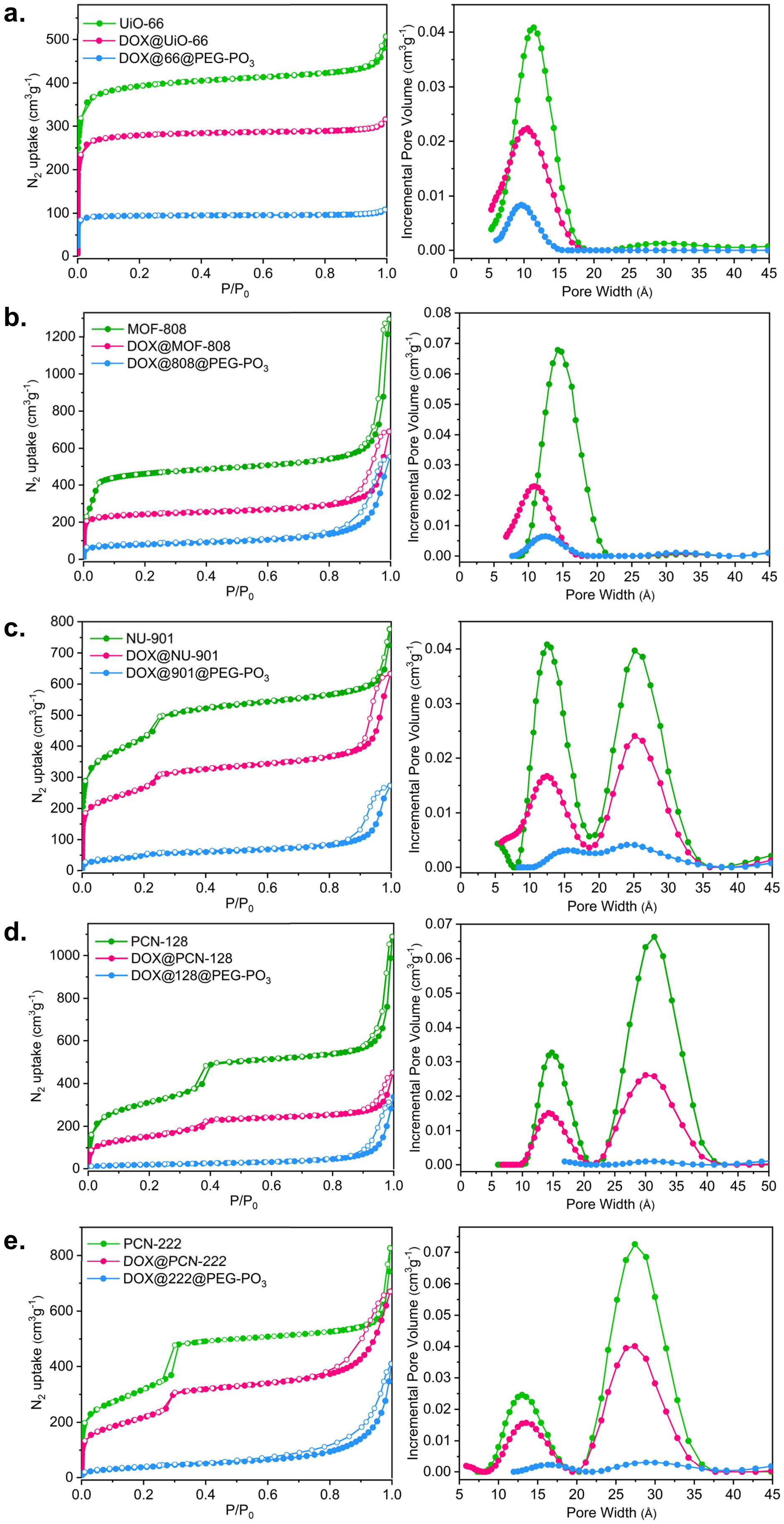

Figure S40. N_2 adsorption isotherms and PSD of DOX@MOF and DOX@MOF@PEG-PO_3. **a.** UiO-66, **b.** MOF-808, **c.** NU-901, **d.** PCN-128 and **e.** PCN-222.

DLS and zeta potential

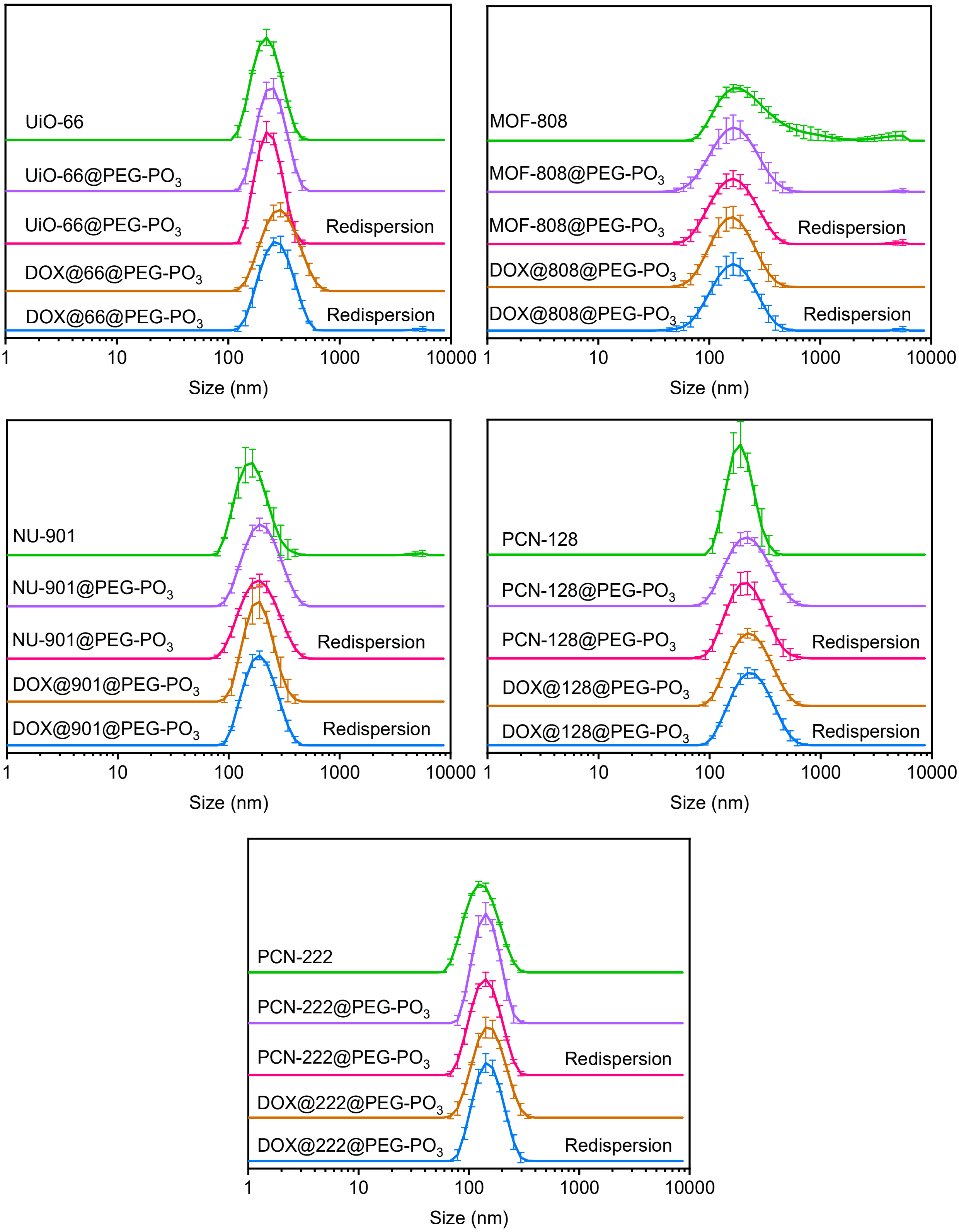

Figure S41. Intensity-average diameter of the suspension of parent nanoMOFs (green line), MOF@PEG-PO_3 before (purple line) and after (pink line) lyophilization, DOX@MOF@PEG-PO_3 before (brown line) and after (blue line) lyophilization (n = 3).

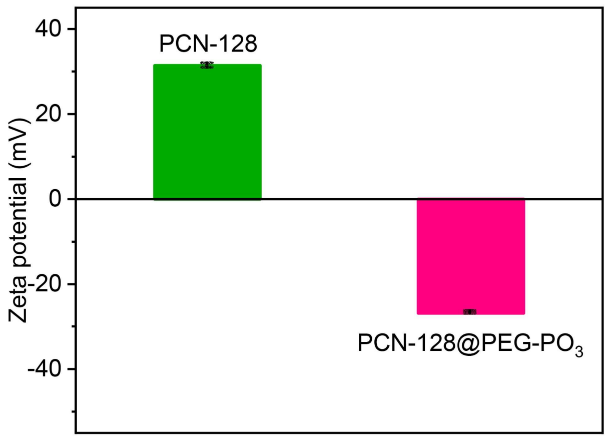

Figure S42. Zeta potential of PCN-128 and PCN-128@PEG-PO$_3$

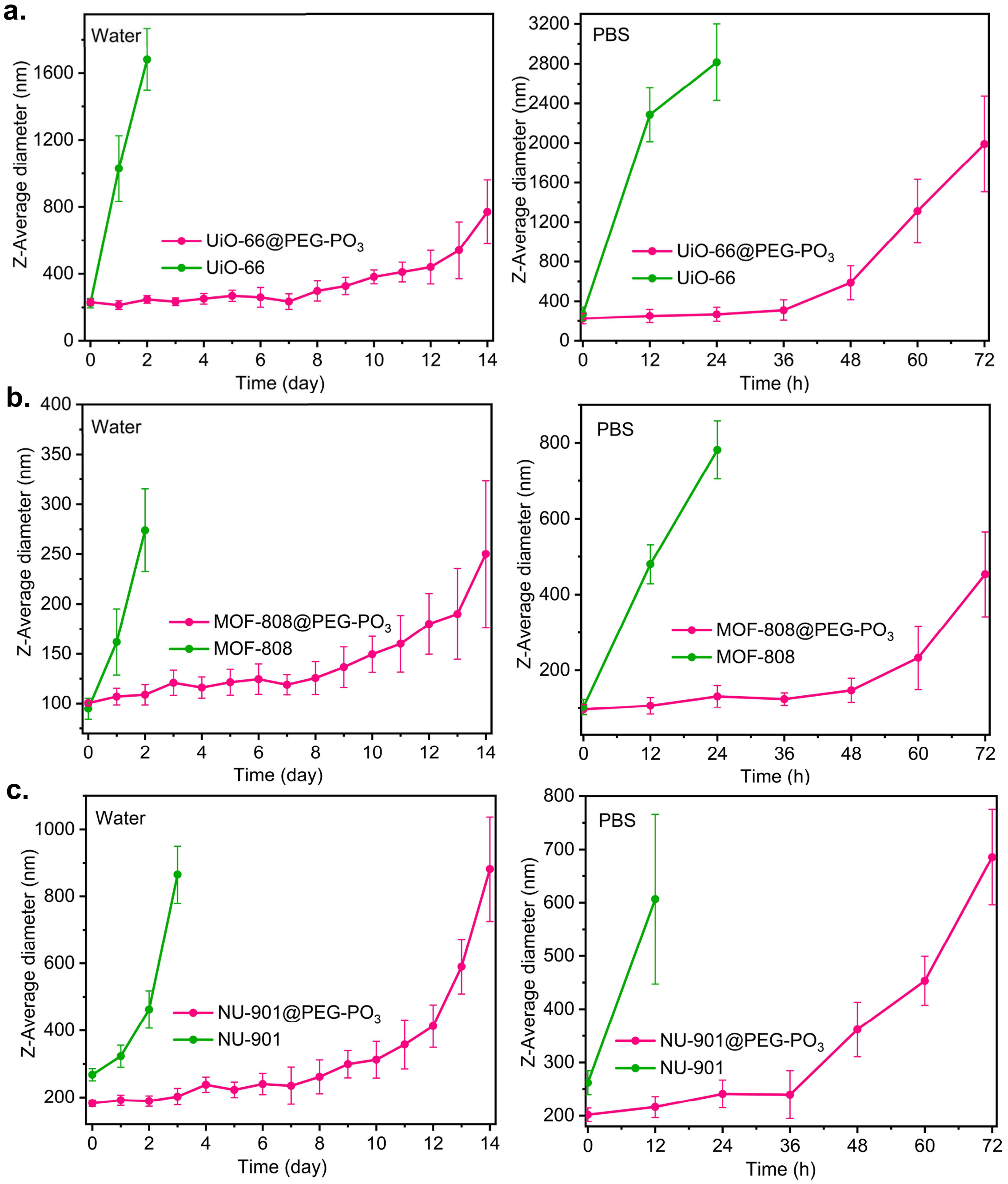

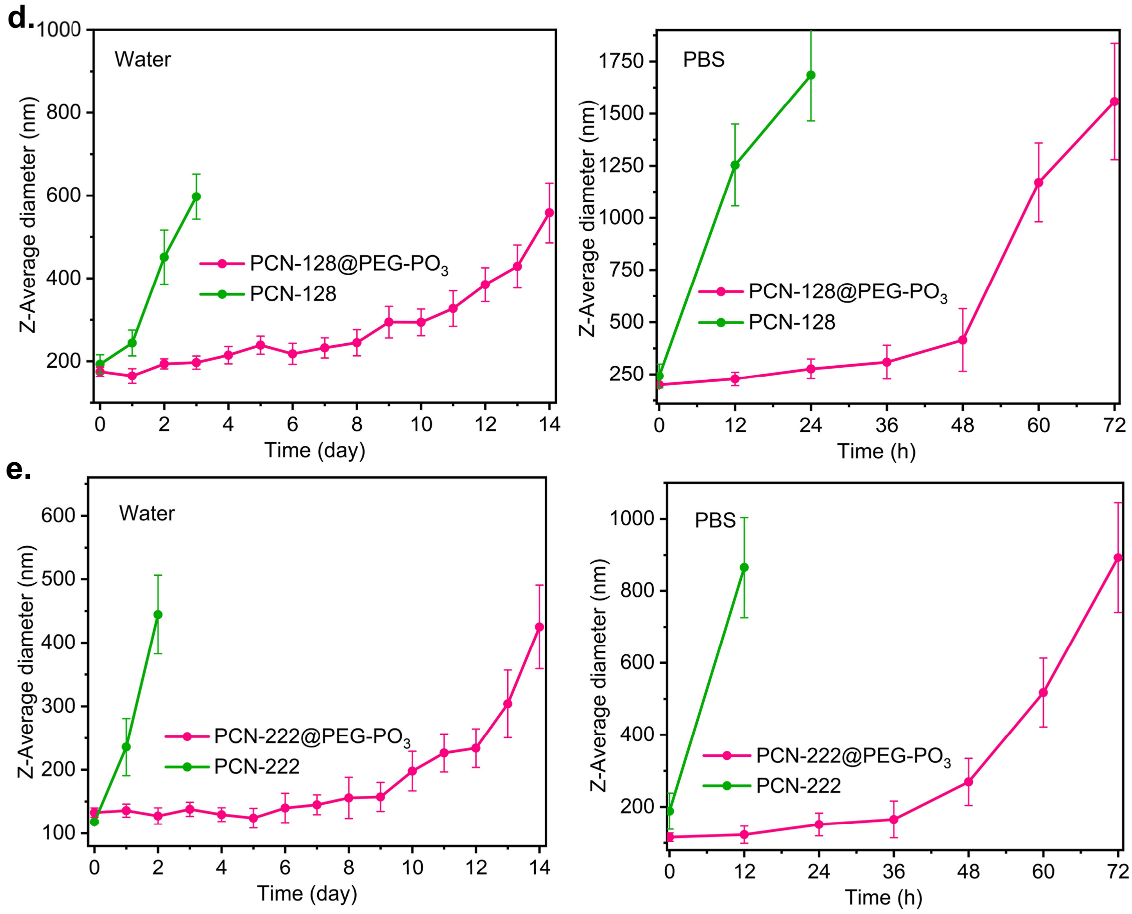

Figure S43. Long-term dispersity of bare MOFs (green line) and MOF@PEG-PO_3 suspended in H_2O or PBS (pH = 7.4). **a.** UiO-66, **b.** MOF-808, **c.** NU-901, **d.** PCN-128 and **e.** PCN-222 The sizes were evaluated by DLS (n=3). During the period of measurement, the samples were left on the tube roller.

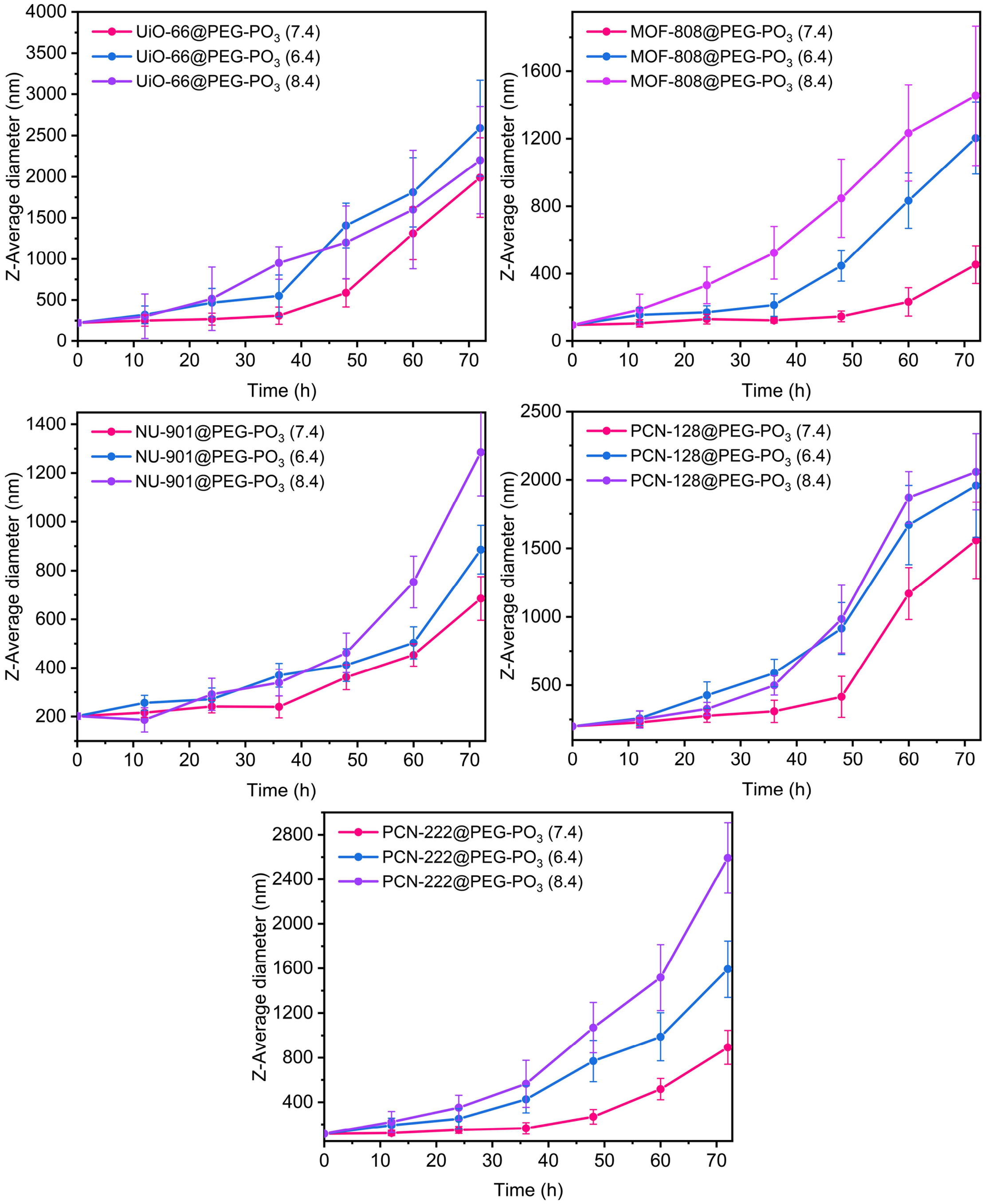

Figure S44. Long-term dispersity of MOF@PEG-PO$_3$ suspended in PBS with different pH values. Sizes were evaluated by DLS (n=3). During the period of measurement, the samples were left on the tube roller.

DOX release studies in PBS (pH = 7.4) and water

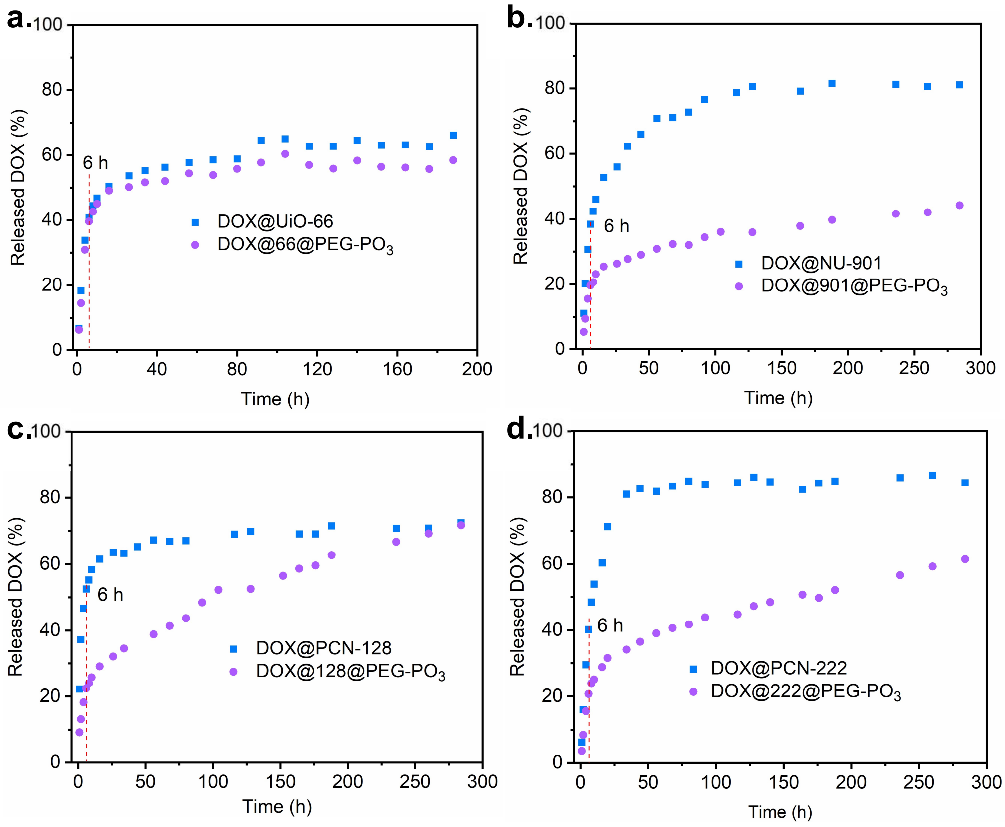

Figure S45. DOX-release profiles from **a.** bare and PEGylated UiO-66, **b.** bare and PEGylated NU-901, **c.** bare and PEGylated PCN-128, **d.** bare and PEGylated PCN-222, in PBS (pH 7.4).

S4. BET area calculation using BETSI[23]

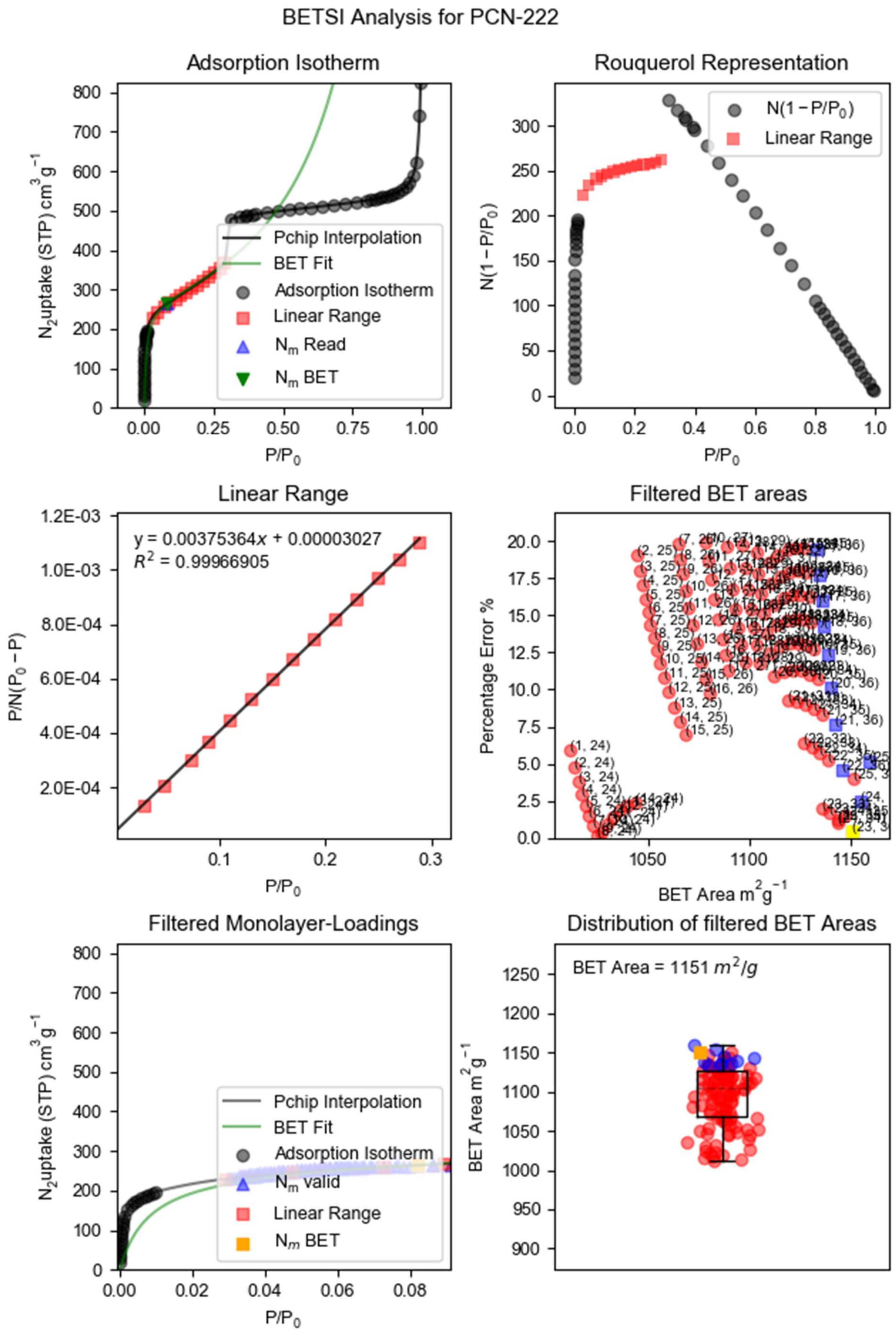

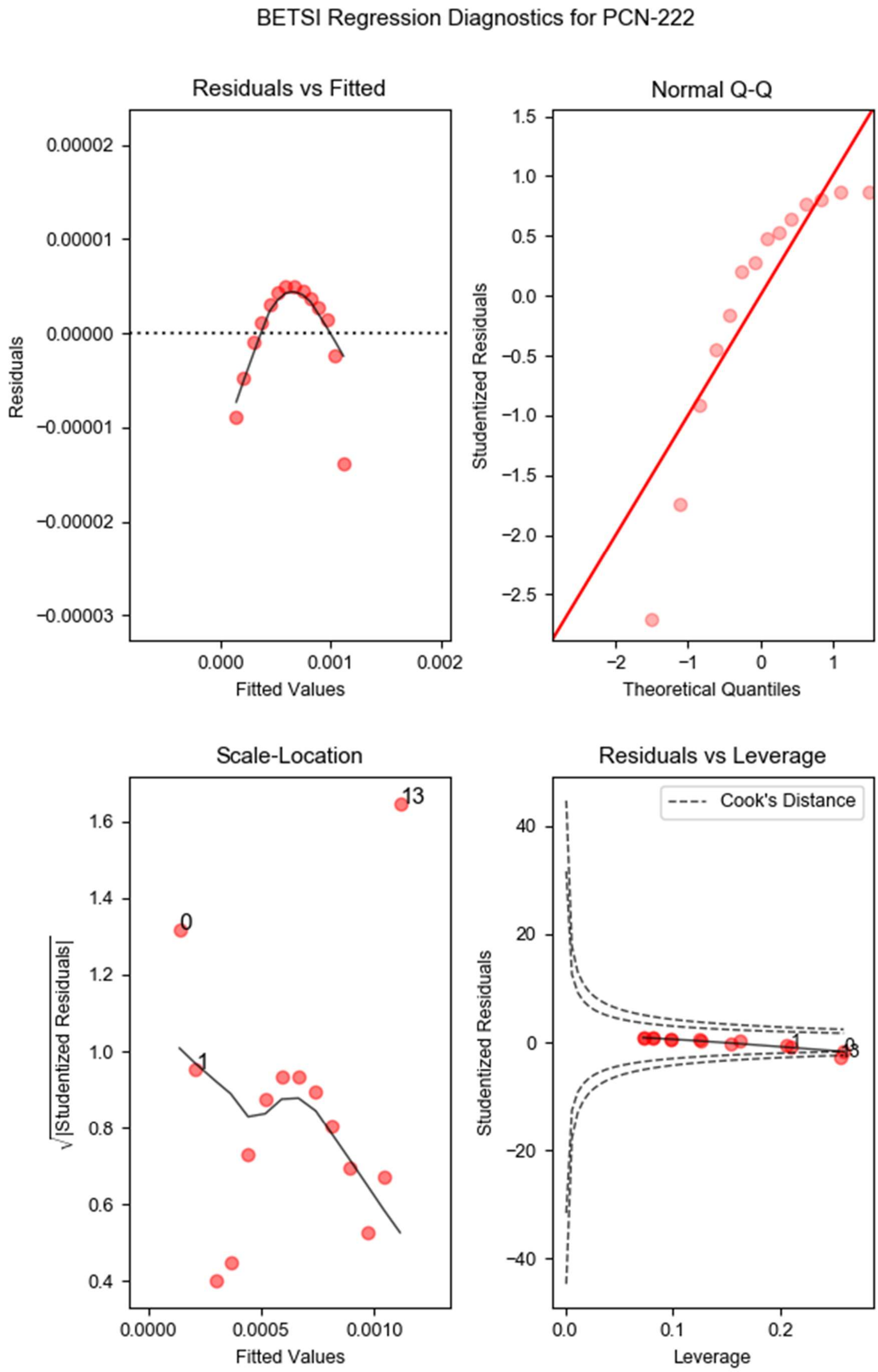
BETSI Regression Diagnostics for PCN-222
Residuals vs Fitted
Residuals
Fitted Values
Normal Q-Q
Studentized Residuals
Theoretical Quantiles
Scale-Location
√|Studentized Residuals|
Fitted Values
Residuals vs Leverage
Cook's Distance
Studentized Residuals
Leverage

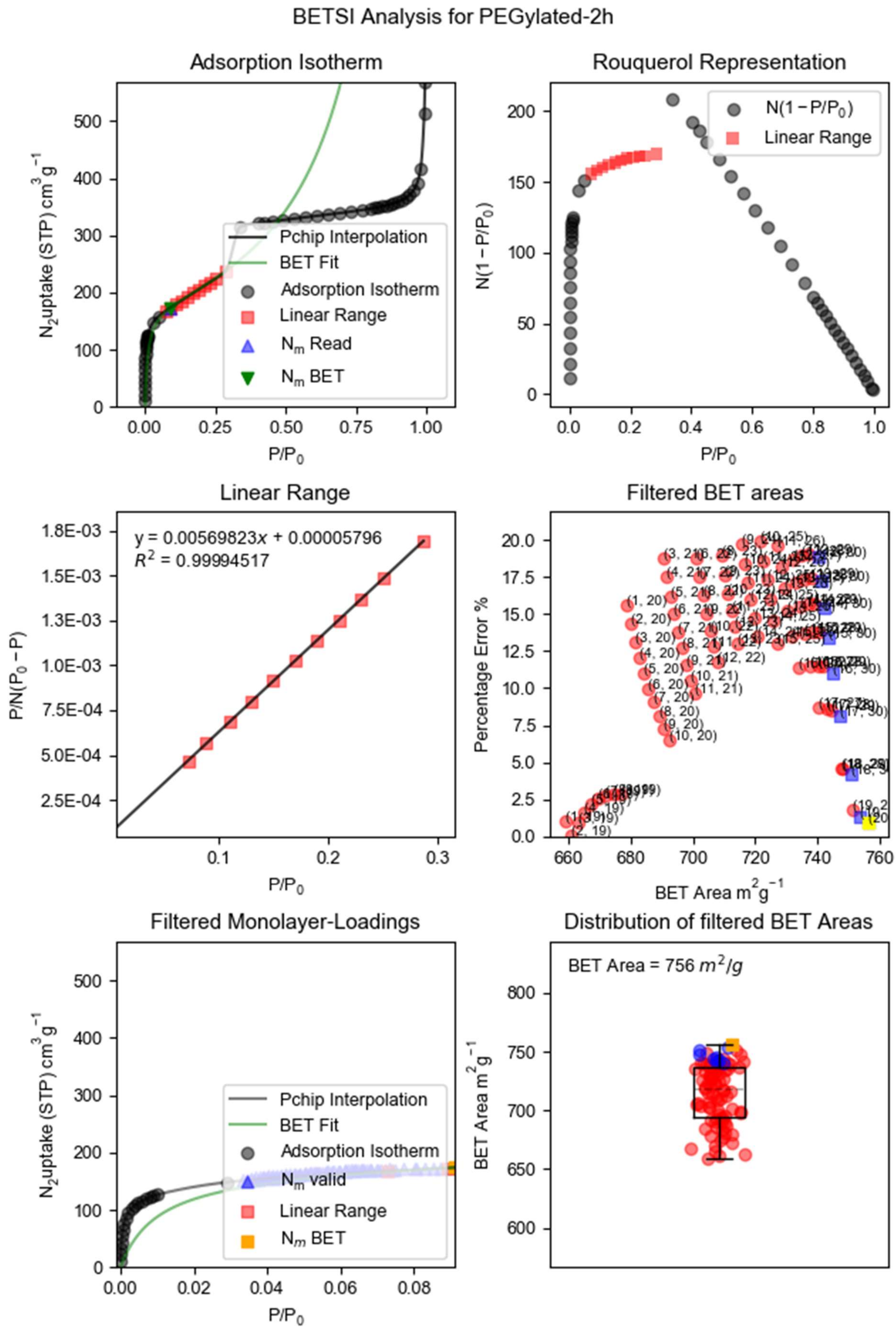
BETSI Analysis for PEGylated-2h
Adsorption Isotherm
Pchip Interpolation
BET Fit
Adsorption Isotherm
Linear Range
N$_m$ Read
N$_m$ BET
N$_2$uptake (STP) cm^{3}g^{-1}
P/P$_0$
Rouquerol Representation
N(1 − P/P$_0$)
Linear Range
P/P$_0$
Linear Range
y = 0.00569823x + 0.00005796
R^2 = 0.99994517
P/N(P$_0$ − P)
P/P$_0$
Filtered BET areas
Percentage Error %
BET Area m^{2}g^{-1}
Filtered Monolayer-Loadings
Pchip Interpolation
BET Fit
Adsorption Isotherm
N$_m$ valid
Linear Range
N$_m$ BET
N$_2$uptake (STP) cm^{3}g^{-1}
P/P$_0$
Distribution of filtered BET Areas
BET Area = 756 m^2/g
BET Area m^{2}g^{-1}

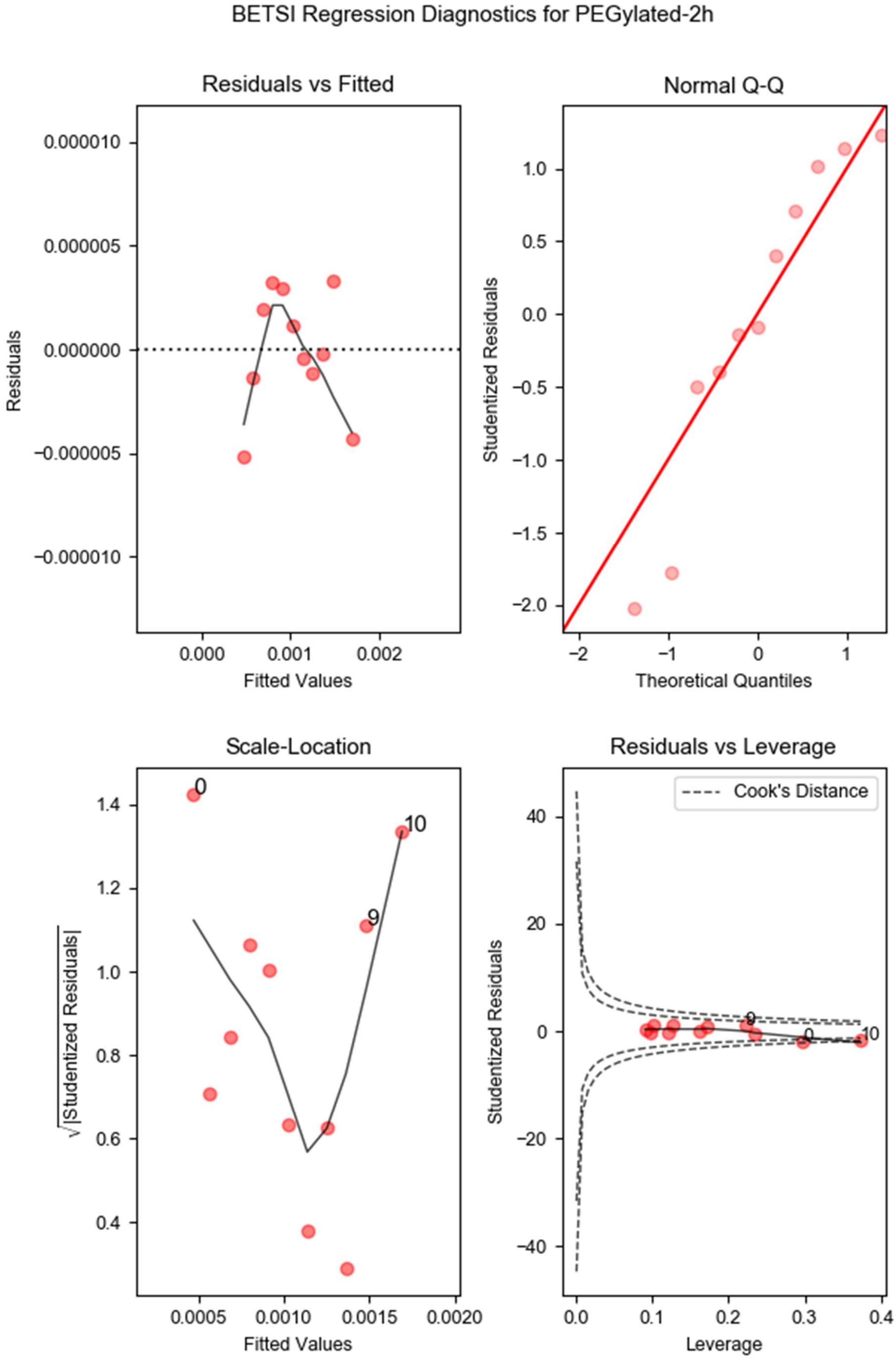
BETSI Regression Diagnostics for PEGylated-2h
Residuals vs Fitted
0.000010
0.000005
0.000000
−0.000005
−0.000010
Residuals
0.000
0.001
0.002
Fitted Values
Normal Q-Q
1.0
0.5
0.0
−0.5
−1.0
−1.5
−2.0
Studentized Residuals
−2
−1
0
1
Theoretical Quantiles
Scale-Location
1.4
1.2
1.0
0.8
0.6
0.4
√|Studentized Residuals|
0.0005
0.0010
0.0015
0.0020
Fitted Values
Residuals vs Leverage
Cook's Distance
40
20
0
−20
−40
Studentized Residuals
0.0
0.1
0.2
0.3
0.4
Leverage

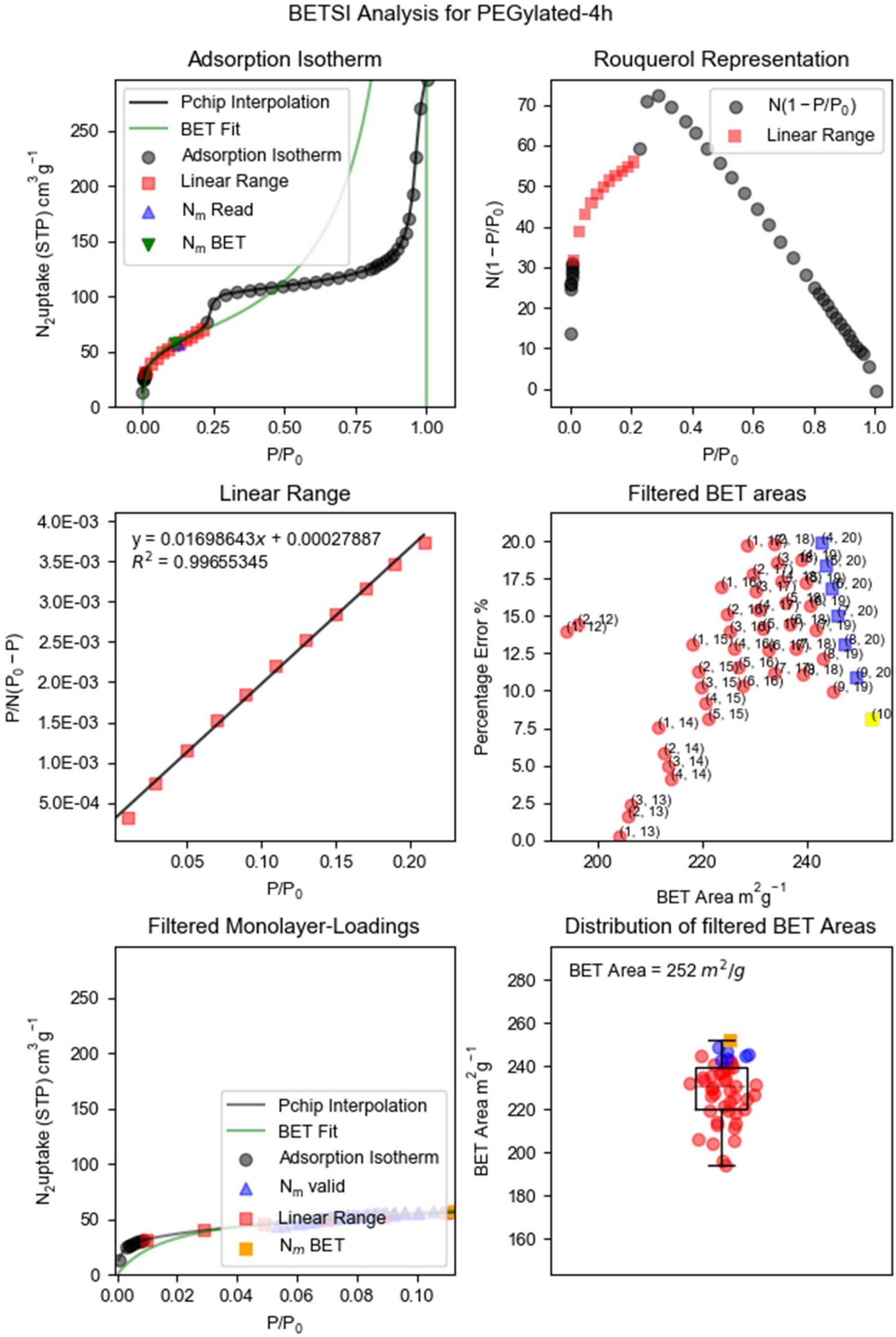
BETSI Analysis for PEGylated-4h
Adsorption Isotherm
Pchip Interpolation
BET Fit
Adsorption Isotherm
Linear Range
N$_m$ Read
N$_m$ BET
N$_2$uptake (STP) cm^3 g^{-1}
P/P$_0$
Rouquerol Representation
N(1 − P/P$_0$)
Linear Range
P/P$_0$
Linear Range
y = 0.01698643x + 0.00027887
R^2 = 0.99655345
P/N(P$_0$ − P)
P/P$_0$
Filtered BET areas
Percentage Error %
BET Area m^{2}g^{-1}
Filtered Monolayer-Loadings
Pchip Interpolation
BET Fit
Adsorption Isotherm
N$_m$ valid
Linear Range
N$_m$ BET
N$_2$uptake (STP) cm^3 g^{-1}
P/P$_0$
Distribution of filtered BET Areas
BET Area = 252 m^2/g
BET Area m^2 g^{-1}

BETSI Regression Diagnostics for PEGylated-4h

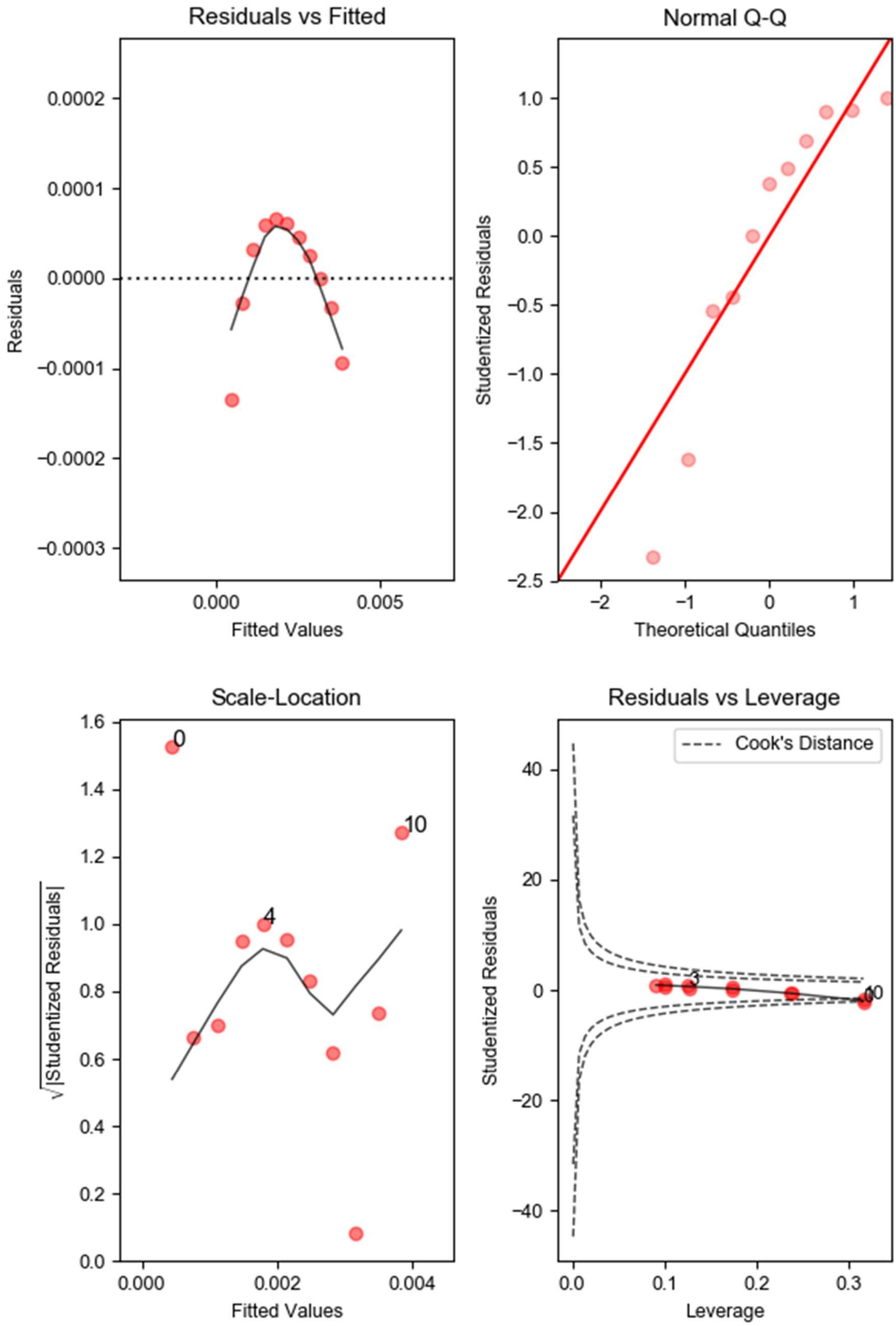

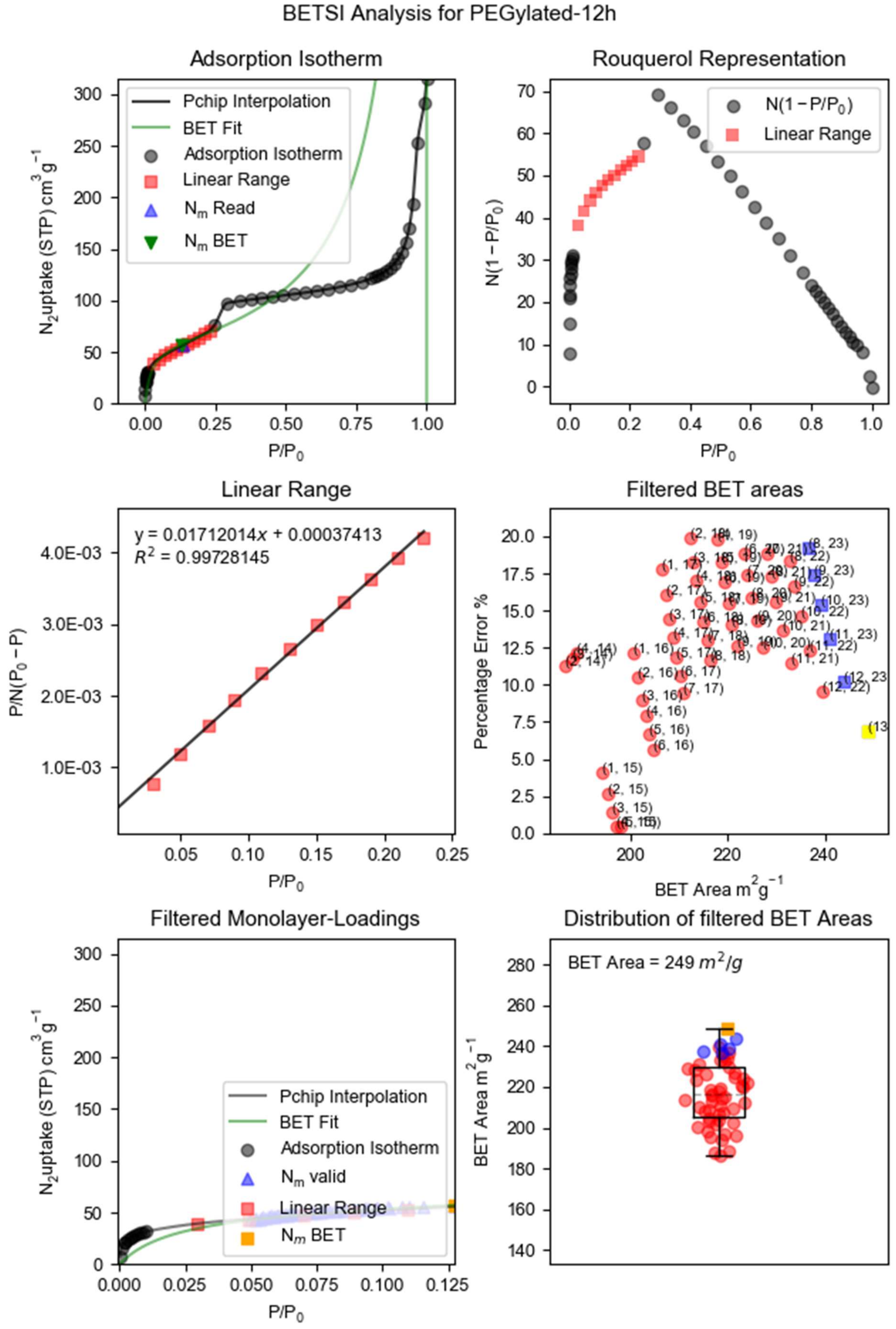

BETSI Analysis for PEGylated-12h
Adsorption Isotherm
Pchip Interpolation
BET Fit
Adsorption Isotherm
Linear Range
N_m Read
N_m BET
N_2 uptake (STP) cm^3 g^-1
P/P_0
Rouquerol Representation
N(1−P/P_0)
Linear Range
Linear Range
y = 0.01712014x + 0.00037413
R^2 = 0.99728145
P/N(P_0−P)
Filtered BET areas
Percentage Error %
BET Area m^2 g^-1
Filtered Monolayer-Loadings
Pchip Interpolation
BET Fit
Adsorption Isotherm
N_m valid
Linear Range
N_m BET
Distribution of filtered BET Areas
BET Area = 249 m^2/g
BET Area m^2 g^-1

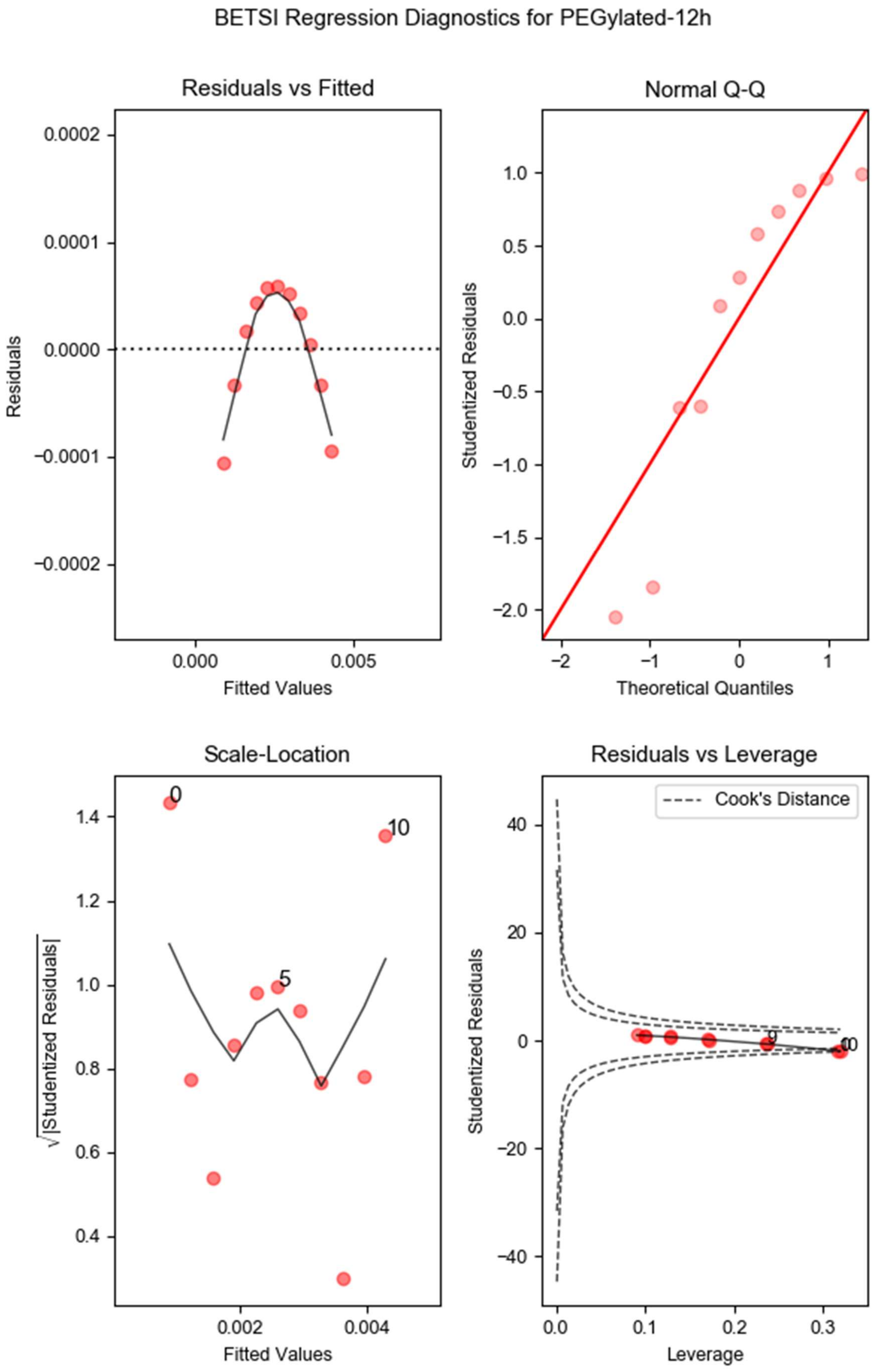
BETSI Regression Diagnostics for PEGylated-12h
Residuals vs Fitted
Residuals
Fitted Values
Normal Q-Q
Studentized Residuals
Theoretical Quantiles
Scale-Location
√|Studentized Residuals|
Fitted Values
Residuals vs Leverage
Cook's Distance
Studentized Residuals
Leverage

BETSI Analysis for PEGylated-16h

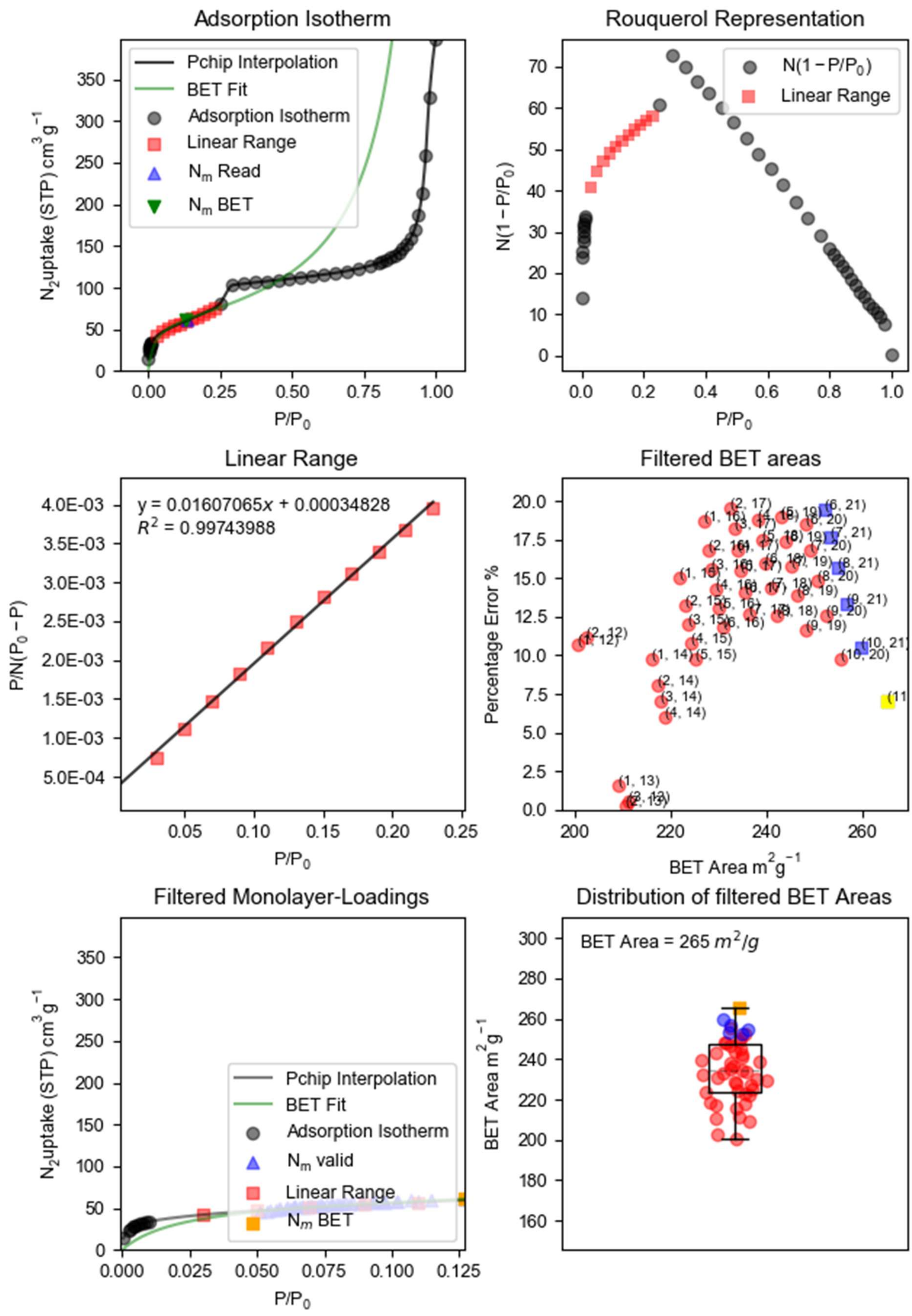

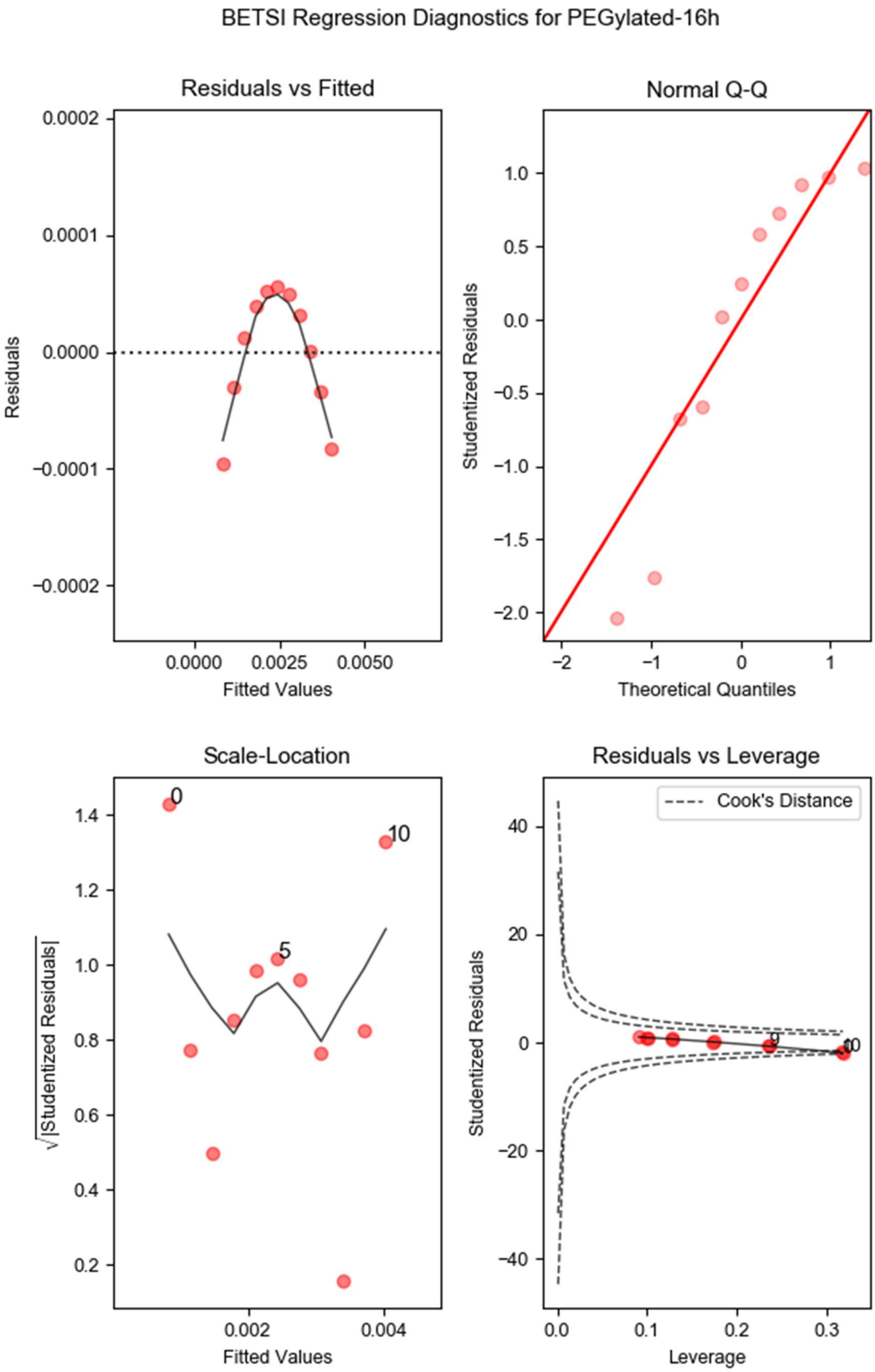
BETSI Regression Diagnostics for PEGylated-16h
Residuals vs Fitted
Residuals
Fitted Values
Normal Q-Q
Studentized Residuals
Theoretical Quantiles
Scale-Location
√|Studentized Residuals|
Fitted Values
Residuals vs Leverage
Cook's Distance
Studentized Residuals
Leverage

S5. MD and GCMC simulations

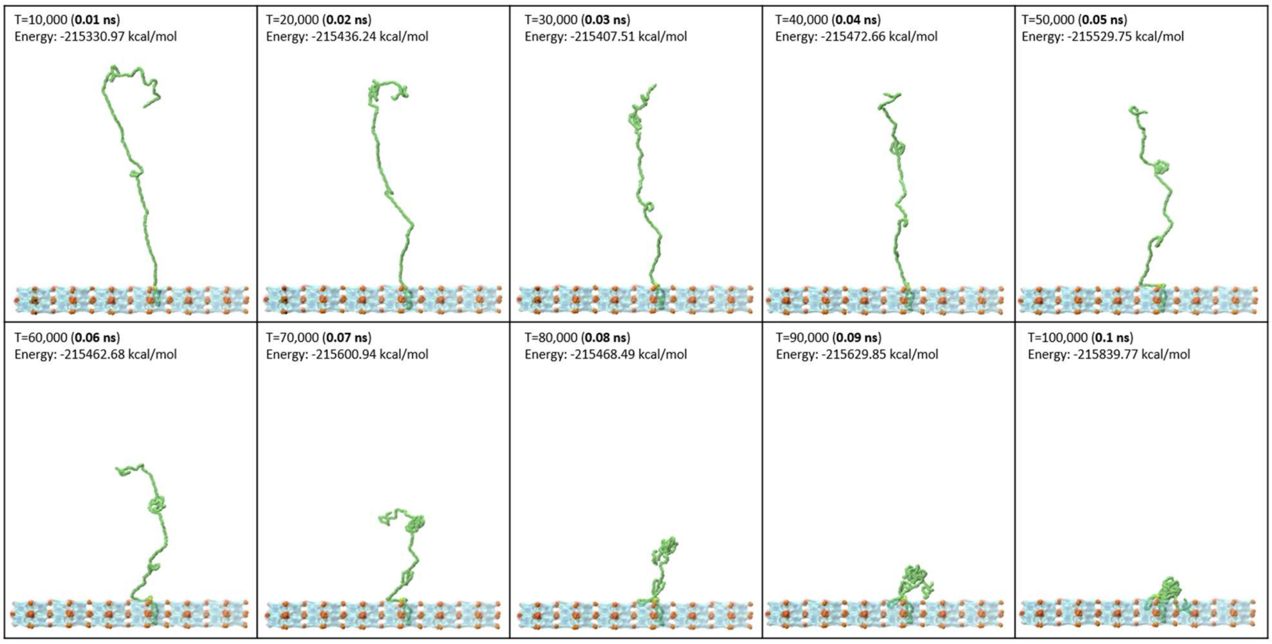

Figure S46. Position of the mPEG-PO_3 relative to the framework as a function of time. The total energy of the system (in kcal/mol) is also given at each time step. PEG atoms (minus the phosphate group) is in green; the phosphate group of the mPEG-PO_3 is in yellow; the framework atoms are in turquoise (translucent), and the Zr atoms of the framework are in brown.

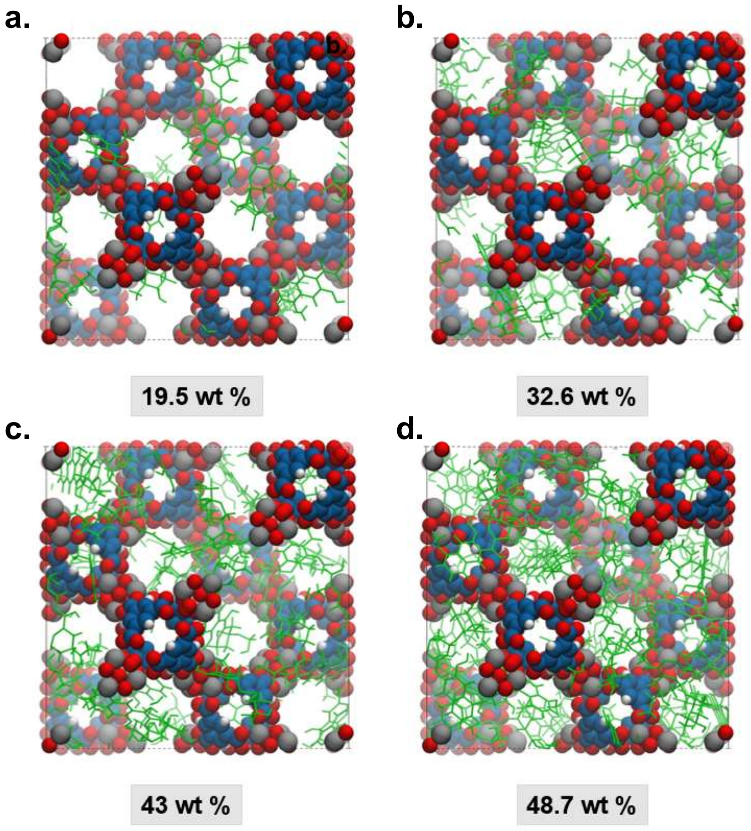

Figure S47. Snapshots of GCMC simulations showing DOX (in green) loading inside MOF-808. **a.**19.5 wt%, **b.** 32.6 wt%, **c.** 43 wt%, and **d.** 48.7 wt%. For the framework, C atoms are in blue, H atoms in white, O atoms in red, and Zr atoms in grey.

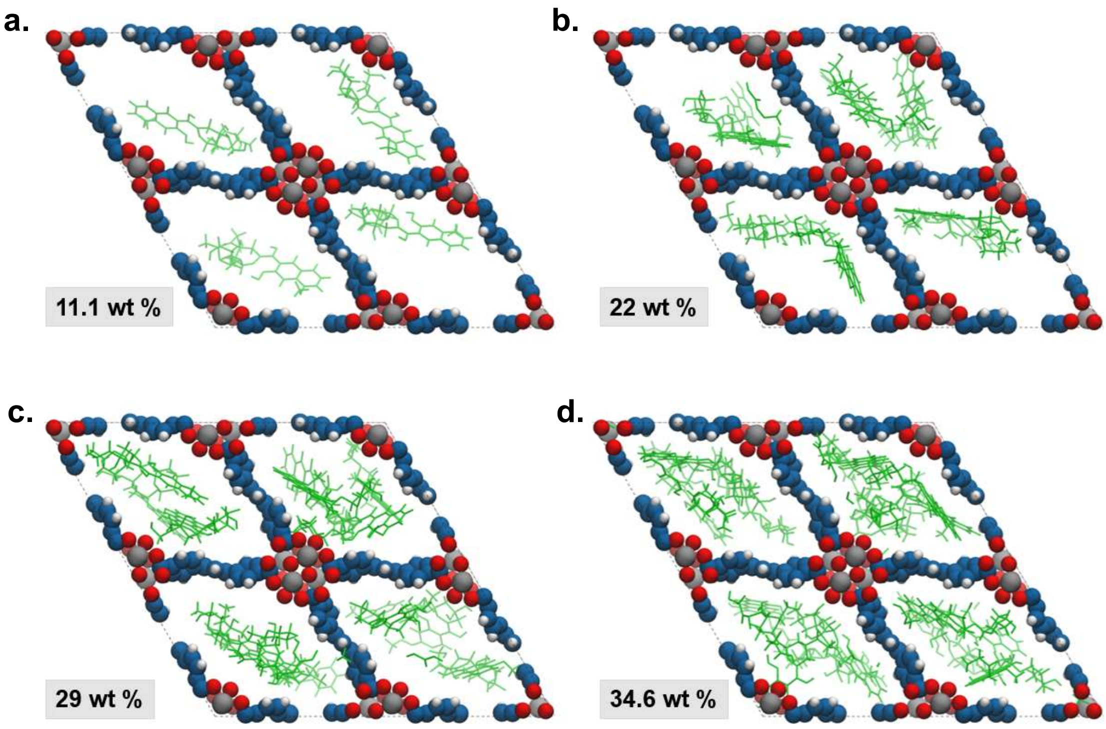

Figure S48. Snapshots of GCMC simulations showing DOX (in green) loading inside NU-901. **a.** 11.1 wt%, **b.** 22 wt%, **c.** 29 wt%, and **d.** 34.6 wt%. For the framework, C atoms are in blue, H atoms in white, O atoms in red, and Zr atoms in grey.

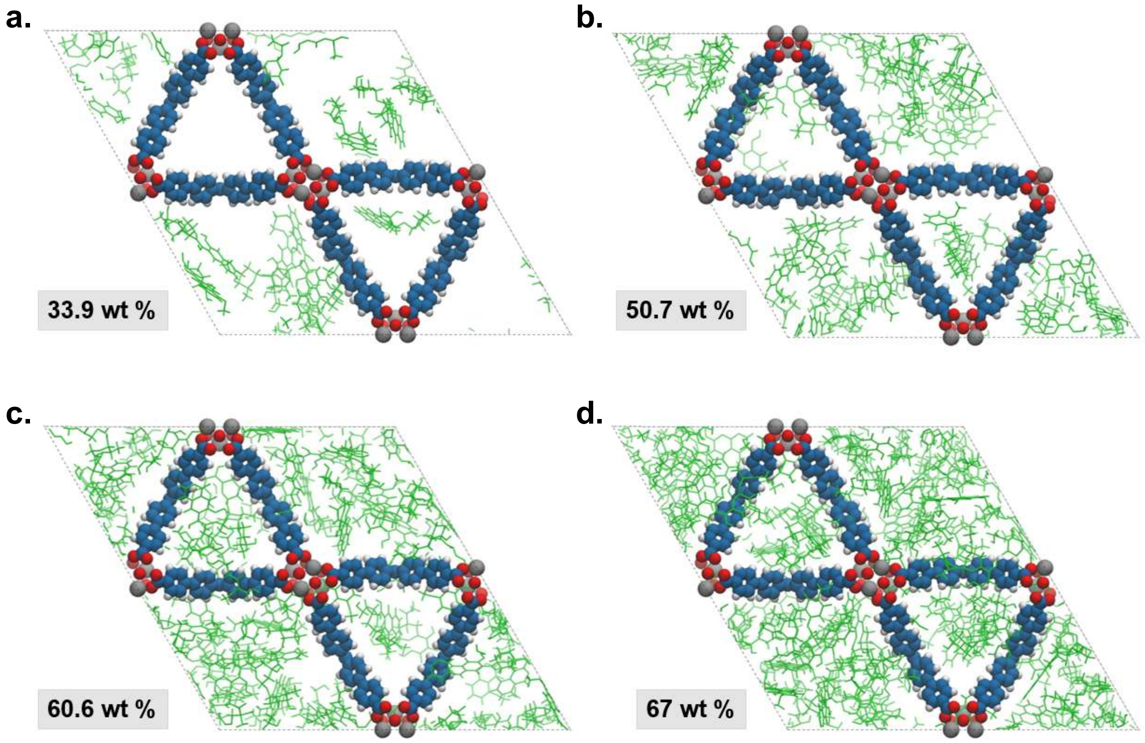

Figure S49. Snapshots of GCMC simulations showing DOX (in green) loading inside PCN-128. **a.** 33.9 wt%, **b.** 50.7 wt%, **c.** 60.6 wt%, and **d.** 67 wt%. For the framework, C atoms are in blue, H atoms in white, O atoms in red, and Zr atoms in grey.

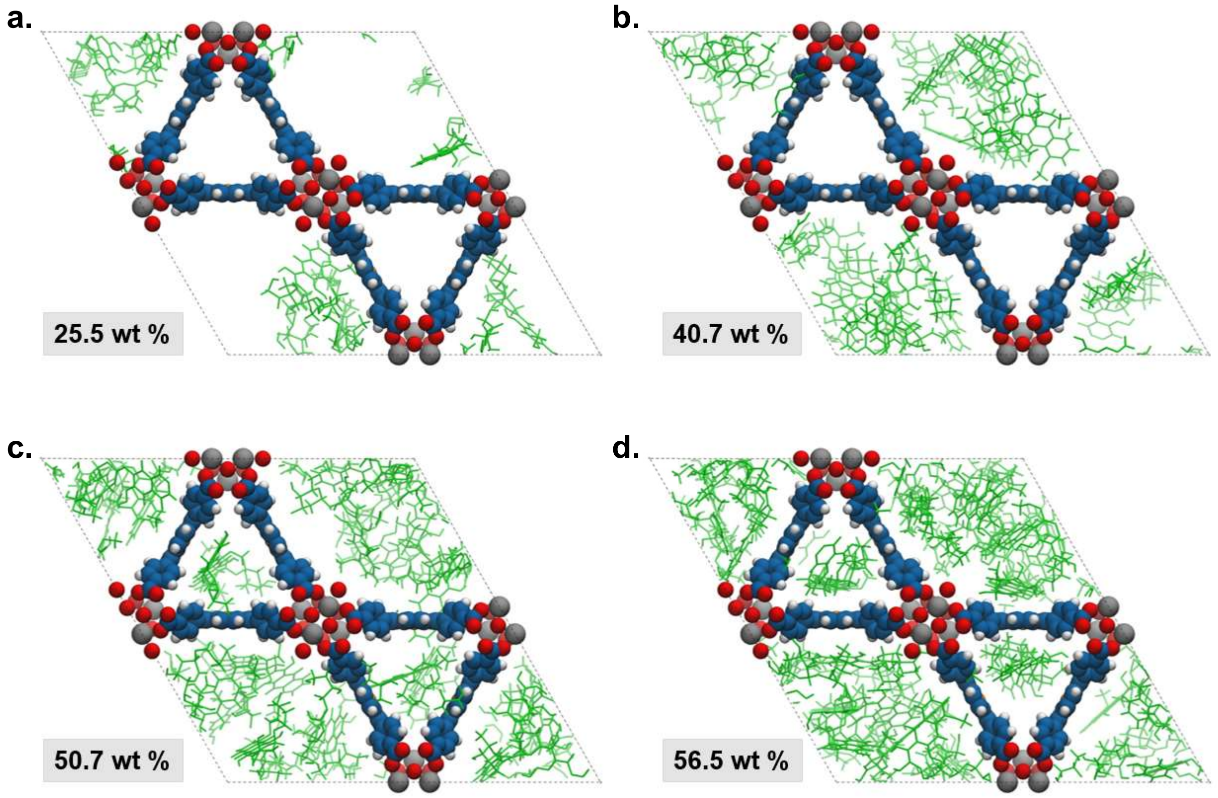

Figure S50. Snapshots of GCMC simulations showing DOX (in green) loading inside PCN-222. **a.** 25.5 wt%, **b.** 40.7 wt%, **c.** 50.7 wt%, and **d.** 56.5 wt%. For the framework, C atoms are in blue, H atoms in white, N atoms in orange, O atoms in red, and Zr atoms in grey.

S6. *In Vitro* evaluation of nanoMOFs

Gating strategy for flow cytometric analysis of nanoMOFs association. Representative dot-plots of HeLa cells were plotted forward versus side scatter using side scatter on a linear scale, with a large gate drawn to exclude debris from the following analysis. Single stain compensations were used to identify the appropriate emission channel of each nanoMOF. Then the single-cell population was plotted against APC channel and Violet 2 channel to identify the percentage of cells associated with nanoMOF at each time point.

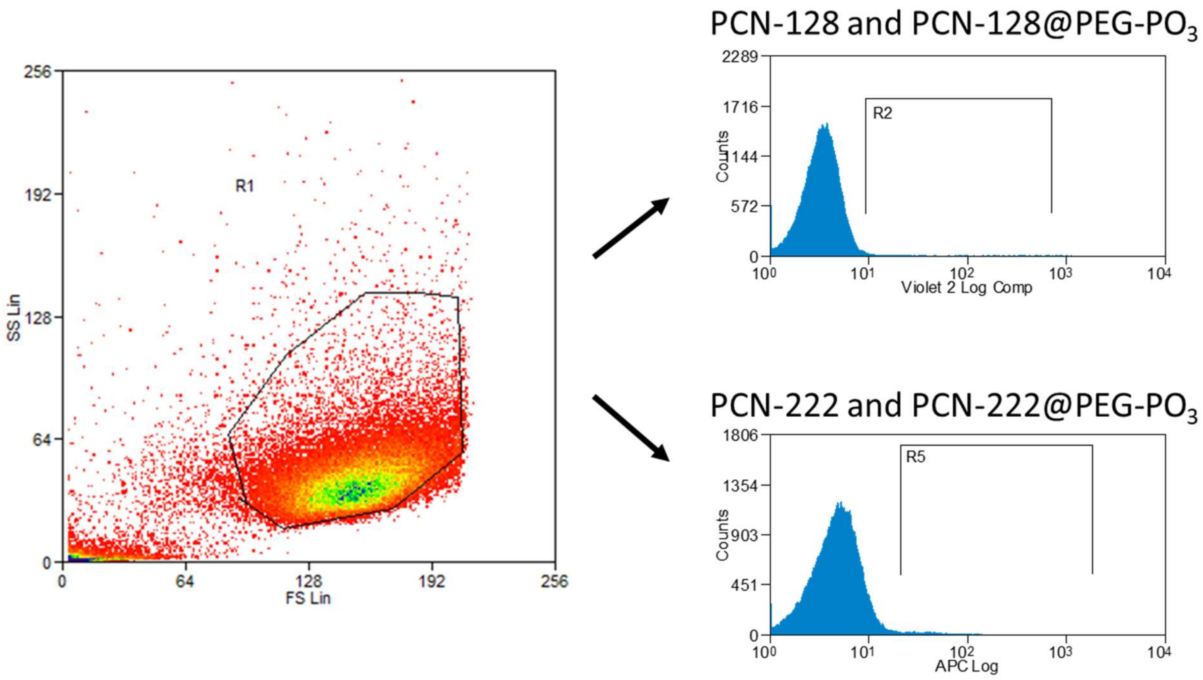

Figure S51. Example analysis of PCN-128, PCN-128@PEG-PO$_3$, PCN-222 and PCN-222@PEG-PO$_3$ using flow cytometry.

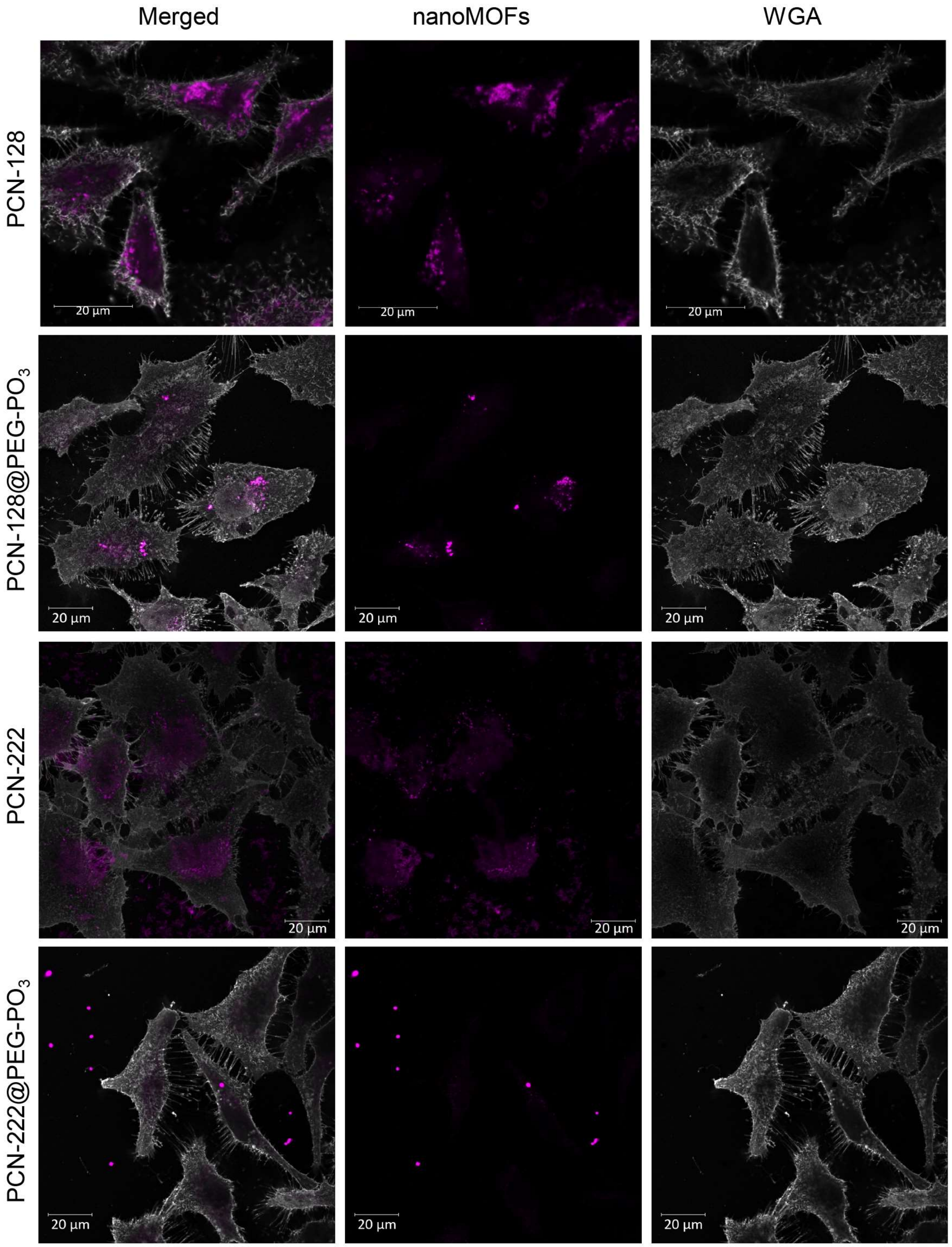

Figure S52. **2D confocal images of PCN-128, PCN-128@PEG-PO$_3$, PCN-222 and PCN-222@PEG-PO$_3$.** HeLa cells were treated with 100 µg/mL for 24 h before they were fixed and stained with wheat germ agglutinin (WGA, colored in white), nanoMOFs in violet with merged images of both channels on the left panel.

Table S1. Comparison of the post-synthetic methods.

MOF	Polymer	Catalyst	Solvent	Temperature
UiO-66	PEG-propargyl	CuI	DCM	r.t.[24]
MIL-100	Acryl-PEG or acryl-HA-PEG	Iron powder	H_2O	r.t.[25]
UiO-66, 67 ,68, PCN-222, 223, 224, MIL-101	Phosphate-terminal oligonucleotides	--	H_2O	r.t.[26]
Zr-MOF	Phosphate-terminal DNA aptamer	--	DMF/H_2O	r.t.[27]
PCN-222, NU-1000	Phosphate-terminal DNA	--	H_2O	r.t.[28]
UiO-66, UiO-67, BUT-30	1,2-dioleoyl-sn-glycero-3-phosphate	--	$CHCl_3$	r.t.[29]
UiO-66, PCN-222	*n*-octadecylphosphonic acid	--	EtOH	r.t.[30]

Table S2. ICP-OES analysis of MOF@PEG-PO_3.

Sample	P (mg/L)	Zr (mg/L)	PEG loading (wt%)
PEGylation 2 h	0.046	5.114	27.8
PEGylation 4 h	0.058	4.972	33.3
PEGylation 12 h	0.071	5.979	33.6
PEGylation 16 h	0.057	4.926	32.9
UiO-66@PEG-PO_3	0.038	3.939	37.7
MOF-808@PEG-PO_3	0.044	4.605	38.5
NU-901@PEG-PO_3	0.042	3.700	30.6
PCN-128@PEG-PO_3	0.057	3.241	34.1

Table S2 shows the mass ratio between mPEG-PO_3 and MOF. The ideal N_2 uptake is calculated based on the assumption that the PEGylation only occurs at the external surface, which is equal to (mass ratio) × actual N_2 uptake of parent MOFs.

Table S3. N_2 uptake at 77 K.

Sample	Actual N_2 uptake at P/P_0 = 0.8 (cm^3g^{-1}, STP)	Ideal N_2 uptake at P/P_0 = 0.8 (cm^3g^{-1}, STP)	Actual uptake/Ideal uptake (%)
PCN-222	526	--	--
PEGylation 2 h	348	380	91.6
PEGylation 4 h	124	351	35.3
PEGylation 12 h	121	349	34.7
PEGylation 16 h	129	353	36.5

Table S4. Experimental, simulated drug loading (max) and the related BET surface area.

Sample	Experimental loading (wt%)	Simulated loading (wt%)	BET area (m^2g^{-1})
DOX@UiO-66	17.6	0	873
DOX@MOF-808	14.4	48.7	771
DOX@NU-901	22.2	34.6	987
DOX@PCN-128	18.1	67.0	541
DOX@PCN-222	23.2	56.5	855
DOX@66@PEG-PO_3	15.1	--	292
DOX@808@PEG-PO_3	13.4	--	265
DOX@901@PEG-PO_3	20.2	--	198
DOX@128@PEG-PO_3	13.6	--	69
DOX@222@PEG-PO_3	15.5	--	145

Table S5. Atom numbers and their corresponding OPLS-AA atom types, along with the Lennard-Jones parameters and charges for the different atoms of the DOX molecule (see below figure).

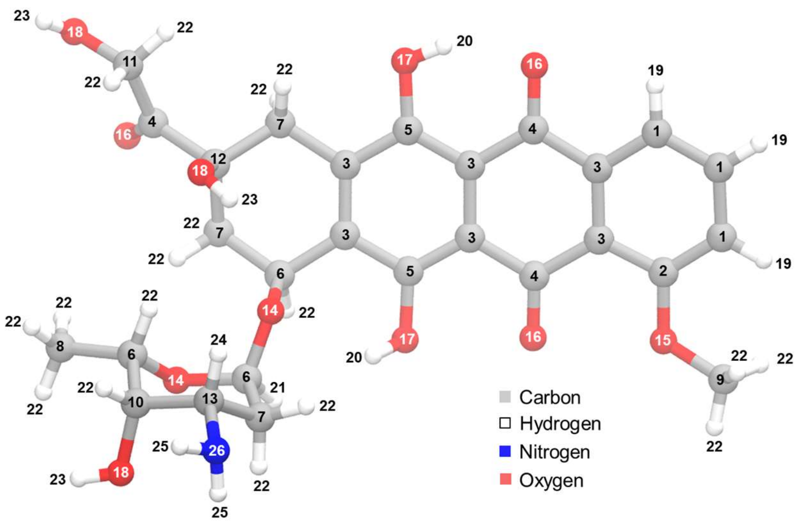

Number	Atom type	ε k_b^{-1} (K)	σ (Å)	q (e)
1	CA	35.22	3.55	-0.115
2	CA	35.22	3.55	0.17
3	CA	35.22	3.55	0
4	C_2	52.83	3.75	0.47
5	CA	35.22	3.55	0.15
6	CT	33.21	3.5	0.17
7	CT	33.21	3.5	-0.12
8	CT	33.21	3.5	-0.18
9	CT	33.21	3.5	-0.065
10	CT	33.21	3.5	0.205
11	CT	33.21	3.5	-0.12
12	CT	33.21	3.5	-0.265
13	CT	33.21	3.5	0.12

14	OS	70.45	2.9	-0.3
15	OS	70.45	2.9	-0.285
16	O_2	105.67	2.96	-0.47
17	OH	85.54	3.07	-0.585
18	OH	85.54	3.12	-0.683
19	HA	15.09	2.42	0.115
20	HO	0	0	0.435
21	HC	15.09	2.5	0.03
22	HC	15.09	2.5	0.06
23	HO	0	0	0.418
24	HC	7.548	2.5	0.06
25	H	0	0	0.36
26	NT	85.54	3.3	-0.9

Table S6. Lennard-Jones parameters for the atoms in the framework**.** Column 1 lists the atom type; columns 2 and 3 list the Lennard-Jones parameters for the corresponding atom types, σ in Å and ε/K_b in K, taken from the DREIDING force field, and for Zr, which is not present in the DREIDING force field, from the UFF (marked with an asterisk).

Atom	LJ Parameters	
	**σ (Å)**	**ε/k$_B$ (K)**
C	3.473	47.888
H	2.846	7.654
N	3.263	38.975
O	3.033	48.190
Zr*	2.783	34.745

S7. References

1. Le Ouay, B.; Watanabe, C.; Mochizuki, S.; Takayanagi, M.; Nagaoka, M.; Kitao, T.; Uemura, T., Selective sorting of polymers with different terminal groups using metal-organic frameworks. *Nat. Commun.* **2018,** *9*, 3635.
2. Hasse, H.; Kany, H. P.; Tintinger, R.; Maurer, G., Osmotic Virial Coefficients of Aqueous Poly(ethylene glycol) from Laser-Light Scattering and Isopiestic Measurements. *Macromolecules* **1995,** *28*, 3540-3552.
3. Feng, D.; Gu, Z. Y.; Li, J. R.; Jiang, H. L.; Wei, Z.; Zhou, H. C., Zirconium-metalloporphyrin PCN-222: mesoporous metal-organic frameworks with ultrahigh stability as biomimetic catalysts. *Angew. Chem. Int. Ed.* **2012,** *51*, 10307-10310.
4. Wilmer, C. E.; Kim, K. C.; Snurr, R. Q., An Extended Charge Equilibration Method. *J. Phys. Chem. Lett.* **2012,** *3*, 2506-2511.
5. Plimpton, S., Fast Parallel Algorithms for Short-Range Molecular Dynamics. *J. Comput. Phys.* **1995,** *117*, 1-19.
6. Rappe, A. K.; Casewit, C. J.; Colwell, K. S.; Goddard, W. A.; Skiff, W. M., UFF, a full periodic table force field for molecular mechanics and molecular dynamics simulations. *J. Am. Chem. Soc.* **1992,** *114*, 10024-10035.
7. Evans, D. J.; Holian, B. L., The Nose–Hoover thermostat. *J. Chem. Phys.* **1985,** *83*, 4069-4074.
8. Humphrey, W.; Dalke, A.; Schulten, K., VMD: Visual molecular dynamics. *J. Mol. Graph.* **1996,** *14*, 33-38.
9. Markopoulou, P.; Panagiotou, N.; Li, A.; Bueno-Perez, R.; Madden, D.; Buchanan, S.; Fairen-Jimenez, D.; Shiels, P. G.; Forgan, R. S., Identifying Differing Intracellular Cargo Release Mechanisms by Monitoring In Vitro Drug Delivery from MOFs in Real Time. *Cell Rep. Phys. Sci.* **2020,** *1*, 100254.
10. Jorgensen, W. L.; Maxwell, D. S.; Tirado-Rives, J., Development and Testing of the OPLS All-Atom Force Field on Conformational Energetics and Properties of Organic Liquids. *J. Am. Chem. Soc.* **1996,** *118*, 11225-11236.
11. Dubbeldam, D.; Calero, S.; Ellis, D. E.; Snurr, R. Q., RASPA: molecular simulation software for adsorption and diffusion in flexible nanoporous materials. *Mol. Simul.* **2015,** *42*, 81-101.
12. Moghadam, P. Z.; Li, A.; Wiggin, S. B.; Tao, A.; Maloney, A. G. P.; Wood, P. A.; Ward, S. C.; Fairen-Jimenez, D., Development of a Cambridge Structural Database Subset: A Collection of Metal–Organic Frameworks for Past, Present, and Future. *Chem. Mater.* **2017,** *29*, 2618-2625.
13. Mayo, S. L.; Olafson, B. D.; Goddard, W. A., DREIDING: a generic force field for molecular simulations. *J. Phys. Chem.* **1990,** *94*, 8897-8909.
14. Wei, Z.; Gu, Z. Y.; Arvapally, R. K.; Chen, Y. P.; McDougald, R. N., Jr.; Ivy, J. F.; Yakovenko, A. A.; Feng, D.; Omary, M. A.; Zhou, H. C., Rigidifying fluorescent linkers by metal-organic framework formation for fluorescence blue shift and quantum yield enhancement. *J. Am. Chem. Soc.* **2014,** *136*, 8269-8276.
15. Noh, H.; Kung, C.-W.; Islamoglu, T.; Peters, A. W.; Liao, Y.; Li, P.; Garibay, S. J.; Zhang, X.; DeStefano, M. R.; Hupp, J. T.; Farha, O. K., Room Temperature Synthesis of an 8-Connected Zr-Based Metal–Organic Framework for Top-Down Nanoparticle Encapsulation. *Chem. Mater.* **2018,** *30*, 2193-2197.
16. Boyer, J. C.; Manseau, M. P.; Murray, J. I.; van Veggel, F. C., Surface modification of upconverting $NaYF_4$ nanoparticles with PEG-phosphate ligands for NIR (800 nm) biolabeling within the biological window. *Langmuir* **2010,** *26*, 1157-1164.
17. Naszalyi Nagy, L.; Mihaly, J.; Polyak, A.; Debreczeni, B.; Csaszar, B.; Szigyarto, I. C.; Wacha, A.; Czegeny, Z.; Jakab, E.; Klebert, S.; Drotar, E.; Dabasi, G.; Bota, A.; Balogh, L.; Kiss, E., Inherently fluorescent and porous zirconia colloids: preparation, characterization and drug adsorption studies. *J. Mater. Chem. B* **2015,** *3*, 7529-7537.
18. Uemura, T.; Yanai, N.; Watanabe, S.; Tanaka, H.; Numaguchi, R.; Miyahara, M. T.; Ohta, Y.; Nagaoka, M.; Kitagawa, S., Unveiling thermal transitions of polymers in subnanometre pores. *Nat. Commun.* **2010,** *1*, 83.
19. Xie, Z.; Wang, B.; Yang, Z.; Yang, X.; Yu, X.; Xing, G.; Zhang, Y.; Chen, L., Stable 2D Heteroporous Covalent Organic Frameworks for Efficient Ionic Conduction. *Angew. Chem. Int. Ed.* **2019,** *58*, 15742-15746.

20. Zhao, G.; Tong, L.; Cao, P.; Nitz, M.; Winnik, M. A., Functional PEG-PAMAM-tetraphosphonate capped $NaLnF_4$ nanoparticles and their colloidal stability in phosphate buffer. *Langmuir* **2014,** *30*, 6980-6989.
21. Li, S.; Amat, D.; Peng, Z.; Vanni, S.; Raskin, S.; De Angulo, G.; Othman, A. M.; Graham, R. M.; Leblanc, R. M., Transferrin conjugated nontoxic carbon dots for doxorubicin delivery to target pediatric brain tumor cells. *Nanoscale* **2016,** *8*, 16662-16669.
22. Li, S. Y.; Zhao, L. P.; Zheng, R. R.; Fan, G. L.; Liu, L. S.; Zhou, X.; Chen, X. T.; Qiu, X. Z.; Yu, X. Y.; Cheng, H., Tumor Microenvironment Adaptable Nanoplatform for O_2 Self-Sufficient Chemo/Photodynamic Combination Therapy. *Part. Part. Syst. Charact.* **2020,** *37*, 1900496.
23. Osterrieth, J.; Rampersad, J.; Madden, D. G.; etc. How Reproducible Are Surface Areas Calculated from the BET Equation? May 26, 2021. *ChemRxiv*. DOI: 10.26434/chemrxiv.14291644.v2 (accessed 2021−07−15).
24. Abanades Lazaro, I.; Haddad, S.; Sacca, S.; Orellana-Tavra, C.; Fairen-Jimenez, D.; Forgan, R. S., Selective Surface PEGylation of UiO-66 Nanoparticles for Enhanced Stability, Cell Uptake, and pH-Responsive Drug Delivery. *Chem* **2017,** *2*, 561-578.
25. Gimenez-Marques, M.; Bellido, E.; Berthelot, T.; Simon-Yarza, T.; Hidalgo, T.; Simon-Vazquez, R.; Gonzalez-Fernandez, A.; Avila, J.; Asensio, M. C.; Gref, R.; Couvreur, P.; Serre, C.; Horcajada, P., GraftFast Surface Engineering to Improve MOF Nanoparticles Furtiveness. *Small* **2018,** *14*, 1801900.
26. Wang, S.; McGuirk, C. M.; Ross, M. B.; Wang, S.; Chen, P.; Xing, H.; Liu, Y.; Mirkin, C. A., General and Direct Method for Preparing Oligonucleotide-Functionalized Metal-Organic Framework Nanoparticles. *J. Am. Chem. Soc.* **2017,** *139*, 9827-9830.
27. Liu, Y.; Hou, W.; Xia, L.; Cui, C.; Wan, S.; Jiang, Y.; Yang, Y.; Wu, Q.; Qiu, L.; Tan, W., ZrMOF nanoparticles as quenchers to conjugate DNA aptamers for target-induced bioimaging and photodynamic therapy. *Chem. Sci.,* **2018,** *9*, 7505-7509.
28. Wang, S.; Chen, Y.; Wang, S.; Li, P.; Mirkin, C. A.; Farha, O. K., DNA-Functionalized Metal-Organic Framework Nanoparticles for Intracellular Delivery of Proteins. *J. Am. Chem. Soc.* **2019,** *141*, 2215-2219.
29. Wang, S.; Morris, W.; Liu, Y.; McGuirk, C. M.; Zhou, Y.; Hupp, J. T.; Farha, O. K.; Mirkin, C. A., Surface-Specific Functionalization of Nanoscale Metal-Organic Frameworks. *Angew. Chem. Int. Ed.* **2015,** *54*, 14738-14742.
30. Sun, Y.; Sun, Q.; Huang, H.; Aguila, B.; Niu, Z.; Perman, J. A.; Ma, S., A molecular-level superhydrophobic external surface to improve the stability of metal–organic frameworks. *J. Mater. Chem. A,* **2017,** *5*, 18770-18776.
